# Supplementary material for: The burden of non-disabled frailty and its associated factors among older adults in Bangladesh
Source: PLoS One. 2023 Nov 28;18(11):e0294889. doi: 10.1371/journal.pone.0294889 (PMC10684086; doi:10.1371/journal.pone.0294889)
Supplement: S1 Table — (DOCX) [file pone.0294889.s001.docx]

**Supplementary table 1:** Factors associated with non-disabled frailty and disability among the participants (N=1045)

| Characteristics | | Disabled | |  | Non-disabled frail | |  |
| --- | --- | --- | --- | --- | --- | --- | --- |
|  | | RRR^1^ | 95% CI | *P* | RRR | 95% CI | *P* |
| Age (years) | |  |  |  |  |  |  |
|  | 60 – 69 | *Ref* |  |  | *Ref* |  |  |
|  | 70-79 | 2.56 | 1.70-3.84 | <0.001 | 1.08 | 0.72-1.61 | 0.717 |
|  | ≥ 80 | 10.00 | 4.41-22.70 | <0.001 | 3.23 | 1.33-7.83 | 0.010 |
| Formal schooling | |  |  |  |  |  |  |
|  | No formal schooling | *Ref* |  |  | *Ref* |  |  |
|  | Having formal schooling | 0.75 | 0.53-1.07 | 0.116 | 0.84 | 0.62-1.14 | 0.275 |
| Family size | |  |  |  |  |  |  |
|  | ≤4 | *Ref* |  |  | *Ref* |  |  |
|  | >4 | 1.25 | 0.86-1.83 | 0.250 | 1.39 | 0.99-1.94 | 0.053 |
| Residence | |  |  |  |  |  |  |
|  | Urban | *Ref* |  |  | *Ref* |  |  |
|  | Rural | 0.61 | 0.39-0.97 | 0.035 | 0.69 | 0.46-1.02 | 0.061 |
| Problems with memory or concentration | |  |  |  |  |  |  |
|  | No problem | *Ref* |  |  | *Ref* |  |  |
|  | Low memory or concentration | 2.73 | 1.89-3.94 | <0.001 | 1.56 | 1.11-2.20 | 0.010 |
| Feeling of loneliness | |  |  |  |  |  |  |
|  | No | *Ref* |  |  | *Ref* |  |  |
|  | Yes | 1.47 | 1.03-2.09 | 0.036 | 1.45 | 1.06-1.98 | 0.019 |
| Family members non-responsive | |  |  |  |  |  |  |
|  | No | *Ref* |  |  | *Ref* |  |  |
|  | Yes | 1.09 | 0.74-1.60 | 0.673 | 1.47 | 1.06-2.04 | 0.021 |

^1^RRR=Relative Risk Ratio; Note: all variables were considered together in this adjusted model
