# Supplementary material for: The burden of non-disabled frailty and its associated factors among older adults in Bangladesh
Source: PLoS One. 2023 Nov 28;18(11):e0294889. doi: 10.1371/journal.pone.0294889 (PMC10684086; doi:10.1371/journal.pone.0294889)
Supplement: Attachment — Submitted filename: PONE-D-23-00513_R1_MMR.pdf [file pone.0294889.s004.pdf]

# PLOS ONE

# The burden of non-disabled frailty and its associated factors among older adults in Bangladesh

--Manuscript Draft--

| | |
|---|---|
| **Manuscript Number:** | PONE-D-23-00513R1 |
| **Article Type:** | Research Article |
| **Full Title:** | The burden of non-disabled frailty and its associated factors among older adults in Bangladesh |
| **Short Title:** | Frailty among older adults in Bangladesh |
| **Corresponding Author:** | Sabuj Kanti Mistry<br>Daffodil International University<br>Dhaka, BANGLADESH |
| **Keywords:** | non-disabled frailty; Older Adults; Bangladesh |
| **Abstract:** | Objective<br>The present study aims to measure the prevalence of non-disabled frailty and its associated factors among Bangladeshi older adults.<br><br>Methods<br>This cross-sectional study was conducted in September 2021 among 1,045 Bangladeshi older adults (≥60 years). Telephone interviews, using a semi-structured questionnaire, collected data on participants' characteristics and levels of frailty. The non-disabled frailty was measured using the 'Frail Non-Disabled (FiND)' questionnaire. A multinomial logistic regression model assessed the factors associated with frailty among the participants.<br><br>Results<br>Around a quarter of the participants (24.8%) were frail. The multinomial regression analysis showed that older participants (RRR= 3.23, 95% CI: 1.41-7.37), those living in a large family with ≥4 members (RRR= 1.39, 95% CI: 1.01-1.92), having memory or concentration problems (RRR= 1.56, 95% CI: 1.12-2.17), family members non-responsive to their needs (RRR= 1.47, 95% CI: 1.06-2.03) and feeling lonely (RRR= 1.45, 95% CI: 1.07-1.98) were more likely to be frail than their counterparts.<br><br>Conclusions<br>The present study's findings suggest providing long-term care tailored towards frail older adults. Specifically, those relatively older, living in large families, suffering from cognitive problems or loneliness, and not properly cared for by their family members should be targeted for relevant health promotion interventions. |
| **Order of Authors:** | Sabuj Kanti Mistry |
| | ARM Mehrab Ali |
| | Uday Narayan Yadav |
| | Saruna Ghimire |
| | Afsana Anwar |
| | Md. Nazmul Huda |
| | Fouzia Khanam |
| | Rashidul Alam Mahumud |
| | Ateeb Ahmad Parray |
| | Shovon Bhattacharjee |
| | David Lim |
| | Mark Fort Harris |

Powered by Editorial Manager® and ProduXion Manager® from Aries Systems Corporation

| Response to Reviewers: | Please see the attachment 'Review response_R1' |
| --- | --- |
| Additional Information: | |
| Question | Response |
| **Financial Disclosure**<br><br>Enter a financial disclosure statement that describes the sources of funding for the work included in this submission. Review the submission guidelines for detailed requirements. View published research articles from *PLOS ONE* for specific examples.<br><br>This statement is required for submission and **will appear in the published article** if the submission is accepted. Please make sure it is accurate.<br><br>**Unfunded studies**<br>Enter: *The author(s) received no specific funding for this work.*<br><br>**Funded studies**<br>Enter a statement with the following details:<br>• Initials of the authors who received each award<br>• Grant numbers awarded to each author<br>• The full name of each funder<br>• URL of each funder website<br>• Did the sponsors or funders play any role in the study design, data collection and analysis, decision to publish, or preparation of the manuscript?<br>• **NO** - Include this sentence at the end of your statement: *The funders had no role in study design, data collection and analysis, decision to publish, or preparation of the manuscript.*<br>• **YES** - Specify the role(s) played.<br><br>* typeset | The author(s) received no specific funding for this work. |
| **Competing Interests**<br><br>Use the instructions below to enter a competing interest statement for this submission. On behalf of all authors, disclose any competing interests that could be perceived to bias this work—acknowledging all financial support and any other relevant financial or non-financial competing interests. | The authors have declared that no competing interests exist. |

Powered by Editorial Manager® and ProduXion Manager® from Aries Systems Corporation

| | |
|---|---|
| This statement is required for submission and **will appear in the published article** if the submission is accepted. Please make sure it is accurate and that any funding sources listed in your Funding Information later in the submission form are also declared in your Financial Disclosure statement.<br><br>View published research articles from *PLOS ONE* for specific examples.<br><br>**NO authors have competing interests**<br><br>Enter: *The authors have declared that no competing interests exist.*<br><br>**Authors with competing interests**<br><br>Enter competing interest details beginning with this statement:<br><br>*I have read the journal's policy and the authors of this manuscript have the following competing interests: [insert competing interests here]*<br><br>* typeset | |
| **Ethics Statement**<br><br>Enter an ethics statement for this submission. This statement is required if the study involved:<br><br>• Human participants<br>• Human specimens or tissue<br>• Vertebrate animals or cephalopods<br>• Vertebrate embryos or tissues<br>• Field research<br><br>Write "N/A" if the submission does not require an ethics statement.<br><br>General guidance is provided below. Consult the submission guidelines for detailed instructions. **Make sure that all** | The study protocol was approved by the Institutional Review Board of the Institute of Health Economics, the University of Dhaka, Bangladesh (Ref: IHE/2020/1037). |

| | |
|---|---|
| **information entered here is included in the Methods section of the manuscript.**<br><br>**Format for specific study types**<br><br>**Human Subject Research (involving human participants and/or tissue)**<br>• Give the name of the institutional review board or ethics committee that approved the study<br>• Include the approval number and/or a statement indicating approval of this research<br>• Indicate the form of consent obtained (written/oral) or the reason that consent was not obtained (e.g. the data were analyzed anonymously)<br><br>**Animal Research (involving vertebrate animals, embryos or tissues)**<br>• Provide the name of the Institutional Animal Care and Use Committee (IACUC) or other relevant ethics board that reviewed the study protocol, and indicate whether they approved this research or granted a formal waiver of ethical approval<br>• Include an approval number if one was obtained<br>• If the study involved *non-human primates*, add *additional details* about animal welfare and steps taken to ameliorate suffering<br>• If anesthesia, euthanasia, or any kind of animal sacrifice is part of the study, include briefly which substances and/or methods were applied<br><br>**Field Research**<br><br>Include the following details if this study involves the collection of plant, animal, or other materials from a natural setting:<br>• Field permit number<br>• Name of the institution or relevant body that granted permission | |
| **Data Availability**<br><br>Authors are required to make all data underlying the findings described fully available, without restriction, and from the time of publication. PLOS allows rare exceptions to address legal and ethical | Yes - all data are fully available without restriction |

| | |
|---|---|
| concerns. See the PLOS Data Policy and FAQ for detailed information.<br><br>A Data Availability Statement describing where the data can be found is required at submission. Your answers to this question constitute the Data Availability Statement and **will be published in the article**, if accepted.<br><br>**Important:** Stating 'data available on request from the author' is not sufficient. If your data are only available upon request, select 'No' for the first question and explain your exceptional situation in the text box.<br><br>Do the authors confirm that all data underlying the findings described in their manuscript are fully available without restriction? | |
| **Describe where the data may be found in full sentences. If you are copying our sample text, replace any instances of XXX with the appropriate details.**<br><br>• If the data are **held or will be held in a public repository**, include URLs, accession numbers or DOIs. If this information will only be available after acceptance, indicate this by ticking the box below. For example: *All XXX files are available from the XXX database (accession number(s) XXX, XXX.*).<br>• If the data are all contained **within the manuscript and/or Supporting Information files**, enter the following: *All relevant data are within the manuscript and its Supporting Information files.*<br>• If neither of these applies but you are able to provide **details of access elsewhere**, with or without limitations, please do so. For example:<br><br>*Data cannot be shared publicly because of [XXX]. Data are available from the XXX Institutional Data Access / Ethics Committee (contact via XXX) for researchers who meet the criteria for access to confidential data.* | All relevant data are within the manuscript and its Supporting Information files. |

| | |
|---|---|
| *The data underlying the results presented in the study are available from (include the name of the third party and contact information or URL).*<br>• This text is appropriate if the data are owned by a third party and authors do not have permission to share the data.<br><br>* typeset | |
| Additional data availability information: | |

Powered by Editorial Manager® and ProduXion Manager® from Aries Systems Corporation

Manuscript

Click here to access/download;Manuscript;Main document_R1_clean.docx

# The burden of non-disabled frailty and its associated factors among older adults in Bangladesh

Sabuj Kanti Mistry[1,2,3], ARM Mehrab Ali[1], Uday Narayan Yadav[2,4], Saruna Ghimire[5], Afsana Anwar[6], Md. Nazmul Huda[1,7], Fouzia Khanam[8], Rashidul Alam Mahumud[9], Ateeb Ahmad Parray[10], Shovon Bhattacharjee[11], David Lim[7,12], Mark Fort Harris[2]

[1] ARCED Foundation, 13/1 Pallabi, Mirpur-12, Dhaka 1216, Bangladesh

[2] School of Population Health, University of New South Wales, Sydney, Australia

[3] Department of Public Health, Daffodil International University, Dhaka 1207, Bangladesh

[4] National Centre for Epidemiology and Population Health, The Australian National University, Canberra, ACT, Australia

[5] Department of Sociology and Gerontology and Scripps Gerontology Center, Miami University, Oxford, OH, USA

[6] Health and Nutrition, Social Assistance and Rehabilitation for the Physically Vulnerable (SARPV), SARPV Complex, Cox's Bazar 4700, Bangladesh

[7] Translational Health Research Institute, Western Sydney University, Campbeltown, NSW 2560, Australia

[8] Department of Public Health, North South University, Dhaka, Bangladesh

[9] NHMRC Clinical Trials Centre, Faculty of Medicine and Health, The University of Sydney, Camperdown, NSW 2006, Australia

[10] Department of International Health, Johns Hopkins Bloomberg School of Public Health, Johns Hopkins University, Baltimore, MD 21218, United States

[11] Biosecurity Program, The Kirby Institute, University of New South Wales, Sydney, Australia.

[12] School of Health Sciences, Western Sydney University, Campbeltown, NSW 2560, Australia

* Correspondence to: Sabuj Kanti Mistry, ARCED Foundation, 13/1 Pallabi, Mirpur-12, Dhaka, Bangladesh, Mobile: +8801716411922, E-mail: smitra411@gmail.com

**Short title:** Frailty among older adults in Bangladesh

# Abstract

## Objective

The present study aims to measure the prevalence of non-disabled frailty and its associated factors among Bangladeshi older adults.

## Methods

This cross-sectional study was conducted in September 2021 among 1,045 Bangladeshi older adults (≥60 years). Telephone interviews, using a semi-structured questionnaire, collected data on participants' characteristics and levels of frailty. The non-disabled frailty was measured using the 'Frail Non-Disabled (FiND)' questionnaire. A multinomial logistic regression model assessed the factors associated with frailty among the participants.

## Results

Around a quarter of the participants (24.8%) were frail. The multinomial regression analysis showed that older participants (RRR= 3.23, 95% CI: 1.41-7.37), those living in a large family with ≥4 members (RRR= 1.39, 95% CI: 1.01-1.92), having memory or concentration problems (RRR= 1.56, 95% CI: 1.12-2.17), family members non-responsive to their needs (RRR= 1.47, 95% CI: 1.06-2.03) and feeling lonely (RRR= 1.45, 95% CI: 1.07-1.98) were more likely to be frail than their counterparts.

## Conclusions

The present study's findings suggest providing long-term care tailored towards frail older adults. Specifically, those relatively older, living in large families, suffering from cognitive problems or

loneliness, and not properly cared for by their family members should be targeted for relevant health promotion interventions.

**Keywords:** non-disabled frailty; older adults; Bangladesh

# Introduction

The world is rapidly transitioning into an ageing society. According to the World Health Organization (WHO), one in six individuals worldwide will be 60 years or older by 2030, and that proportion will quadruple by 2050 [1]. Low- and middle-income countries (LMICs) are projected to have two-thirds of their population aged 60 or older by 2050 [1]. In Bangladesh, reflecting this global demographic shift, the total number of older adults aged 60 years and above is expected to increase from 8 million to 44 million by 2050 [2]. This demographic change has resulted in an increased burden of conditions common in older age, including various non-communicable diseases (NCDs) and frailty [3]; the latter is now recognized as an emerging concern among older adults in South Asian countries, including Bangladesh [4].

[illegible]ilty is a condition characterized by low physical activity, cognitive performance, and nutritional status. Fried and colleagues [6] provided the operational definition of frailty, which states that frailty is a condition where three out of five phenotypic criteria are met: shrinkage (weight loss), exhaustion, weaknesses, low gait speed, and low physical activity. Frailty predicts many long-term health outcomes, disability and mortality [5] and interferes with one's ability to live independently [7]. Frailty is common in older age due to biological ser[illegible]ence that results in the deterioration of physical, cognitive, and psychological functions [8]. This condition manifests as a state of increased vulnerability and decreased resilience, resulting in decreased physical activity, diminished cognitive performance, and compromised nutritional status [7].

There is evidence indicating a high burden of frailty in LMICs. A systematic [illegible]iew and meta-analysis conducted with 56 studies in LMICs documented that the prevalence of frailty and

prefrailty among community-dwelling older adults was 17.4% and 49.3%, respectively [9]. The prevalence of frailty was found to be higher among older adults from South Asian countries. For instance, a Nepalese study reported that 65% of participants had frailty without disability [10]. In India, 26% of older adults were frail, while 63.6% were pre-frail, and only 10.4% were non-frail [11]. Studies conducted in Pakistan [12] and Sri Lanka [13] found that 55.4% and 15.2% of older adults were frail, with 44.6% and 48.5% being pre-frail, respectively. These studies [10, 11, 13] also identified several determinants of frailty among older adults, such as age, education, occupation, out-of-pocket health care cost, depression, and lack of physical activities and care from family members.

Limited evidence exists on the prevalence and determinants of frailty among older adults in Bangladesh. The only study on frailty among Bangladeshi older adults showed that 61.6% of the participants had moderate to severe levels of frailty [14]. However, this study was conducted in a small geographical area of the country and used the 30-indicator Frailty Index scale that does not measure non-disabled frailty. Considering the high burden of NCDs coupled with limited healthcare provision in Bangladesh, we anticipate a high prevalence of frailty. Therefore, further research is needed to investigate this topic in the Bangladeshi context. Accordingly, the present study aims to quantify the burden of frailty and identify associated factors among Bangladeshi older adults.

## Materials and methods

### Study design and participants

This cross-sectional study was conducted in September 2021. Previously, between 2016–2020, the ARCED Foundation conducted ten different community-based studies and established a registry using demographic information from these studies, which served as the sampling frame for the current study. Notably, this sampling frame included households from all eight administrative divisions of Bangladesh. Considering a 50% unknown prevalence with a 5% margin of error, 95% confidence level, and 90% power, a sample size of 1096 was calculated. However, only 1045 of 1096 approached participants responded to the study resulting in an overall response rate of approximately 95%. Based on the population distribution of older people by geography in Bangladesh, we adopted a probability proportionate to size (of the eight administrative divisions) approach to determine the size of older people in each division [17]. The inclusion criterion was the minimum age of 60 years of the participants. Exclusion criteria were suffering from any adverse mental conditions i.e., clinically diagnosed schizophrenia, bipolar mood disorder, dementia/cognitive impairment, and inability to communicate.

**Measures**

*Outcome measure*

The outcome of interest was non-disabled frailty, measured using the ‘Frail Non-Disabled (FiND) questionnaire’ [18]. The FiND questionnaire is designed to measure non-disabled frailty among the older population and was previously used among older adults in Nepal in a similar setting [5]. The FiND questionnaire contains five questions under two sections. The first two questions on mobility (i.e., items A and B) belong to the disability section, and the latter three on weight loss, exhaustion, and physical activity (i.e., items C, D and E) belong to the frailty section. Each five items are coded as 0 and 1, indicating negative and affirmative responses, respectively. Participants

are categorized into one of three categories, i.e., “disabled,” “frail,” and “robust.” If a participant responds affirmatively to at least one of the two disability items (i.e., item $A+B\geq 1$), they are classified as disabled. Frailty is when a participant responds negatively to both disability questions but affirmatively to at least one frailty item (i.e., $A+B=0$ and $C+D+E\geq 1$). A participant is classified as “robust” when the sum of five items is zero, i.e., indicating the absence of both frailty or disability.” A Cronbach α of 0.75 in the current study indicated the scale is reliable among our study population.

*Explanatory variables*

An extensive literature review guided the selection of explanatory variables [10-13, 15]. Explanatory variables considered in this study were administrative divisions (Barishal, Chattogram, Dhaka, Mymensingh, Khulna, Rajshahi, Rangpur, Sylhet), age (categorized as 60-69, 70-79, and ≥80), sex (male/female), marital status (married and without a partner; latter included widowed, separated and never married), formal schooling (with and without formal schooling), family size (≤4 or >4), family monthly income in Bangladeshi Taka (BDT) (<5,000, 5,000-10,000, >10,000), residence (urban/rural), current occupation (employed, unemployed or retired), living arrangement (living alone or with family), walking distance to the nearest health center (<30 min/≥30 min), memory or concentration problems (no problem/low memory or concentration), suffering from at least one non-communicable chronic conditions (yes/no), a perception that family members are non-responsive to their needs (yes/no), and feeling of loneliness (yes/no).

Self-reported information on the participant’s ever diagnosis of nine non-communicable chronic conditions (i.e., arthritis, hypertension, heart diseases, stroke, hypercholesterolemia, diabetes,

chronic respiratory diseases, chronic kidney disease, and cancer) was collected as dichotomized yes/no responses. To explore if participants had any memory or concentration problems, we asked the following question: ‘Do you have any memory (remembering things properly) or concentration (focusing/concentrating properly while doing any action) problem?’ Loneliness was measured using the 3-item UCLA Loneliness scale [19]. Each item on the scale is measured as a yes/no question. The three items included: how often do you feel: (i) lack companionship, (ii) left out, and (iii) isolated in the last two weeks? Each item in the scale is measured in terms of 3-item Likert responses: hardly ever (1 point), some of the time (2 points), and often (3 points). Participants were classified as lonely if they answered ‘some of the time’ or ‘often’ to any of the three items [19].

**Data collection tools and techniques**

Telephone interviews were conducted using a pre-tested semi-structured questionnaire, and data were collected electronically using SurveyCTO mobile app (https://www.surveycto.com/). Ten research assistants were recruited as surveyors based on their previous experiences administering health surveys on the electronic platform. The research assistants were trained extensively via Zoom meetings for three full days by the coauthors (SKM, AMA, UNY) before beginning the data collection.

The English version of the questionnaire was first translated into Bengali and then back-translated to English by two researchers (SKM and AMA) to ensure the content’s construct and face validity. The questionnaire was then piloted among a small sample (n=10) of older adults for cultural validation of contents and refinement of the questions. The piloted response, excluded from data

analyses, did not provide any corrections/suggestions and thus was used as is for the data collection. Each interview took around half an hour.

## Statistical analyses

Descriptive analyses, i.e., frequency and percentage, were performed to explore the distribution of the sociodemographic characteristics of the respondents. To explore the factors independently associated with non-disabled frailty, multinomial logistic regression models were run, adjusting for important confounders. Covariates for adjustment were identified using the backward elimination method based on the Akaike information criterion (AIC). The initial model was run with all potential covariates listed in Table 1, and only age, formal schooling, family size, residence, problems with memory or concentration, loneliness, and non-responsive family members were retained/adjusted in the final model. The final model was also tested for sensitivity using the bootstrapping approach by resampling observations with 10,000 replications. The Relative Risk Ratio (RRR) and associated 95% confidence interval (95% CI) for the "disabled" and "frail" categories are provided with reference to the "robust" category in Table 3. All analyses were performed using the statistical software package Stata (Version 14.0).

## Ethics approval

The study protocol was approved by the Institutional Review Board of the Institute of Health Economics, the University of Dhaka, Bangladesh (Ref: IHE/2020/1037). Verbal informed consent was sought from the participants before administering the survey.

## Patient and public involvement

Patients and/or the public were not involved in developing research questions, designing and conducting the study, and disseminating the results.

## Results

### Characteristics of the participants

The sociodemographic characteristics of the participants are presented in Table 1. The majority of the participants were aged 60-69 years (75.6%), males (59.3%), and married (76.5%). More than half of the participants (51.7%) had no formal schooling, 66.8% belonged to a large family of more than four members, 82.6% lived in rural areas, and 61.1% were unemployed or retired during the survey period. Furthermore, 35.3% had problems with memory or concentration, 57.2% suffered from at least one non-communicable chronic condition, 29.4% had their family members non-responsive to their care, and 45.7% felt lonely (Table 1).

**Table 1:** Characteristics of study participants (N=1045)

| Characteristics | N | % |
|---|---|---|
| Administrative division | | |
| Barishal | 146 | 14.0 |
| Chattogram | 98 | 9.4 |
| Dhaka | 172 | 16.5 |
| Mymensingh | 69 | 6.6 |
| Khulna | 198 | 19.0 |
| Rajshahi | 145 | 13.9 |
| Rangpur | 161 | 15.4 |
| Sylhet | 56 | 5.4 |

| | | |
|---|---|---|
| Residence | | |
| Urban | 182 | 17.4 |
| Rural | 863 | 82.6 |
| Age (in years) | | |
| 60 – 69 | 790 | 75.6 |
| 70-79 | 201 | 19.2 |
| ≥ 80 | 54 | 5.2 |
| Sex | | |
| Male | 620 | 59.3 |
| Female | 425 | 40.7 |
| Marital status | | |
| Married | 799 | 76.5 |
| [1]Without partner | 246 | 23.5 |
| Formal schooling | | |
| No formal schooling | 540 | 51.7 |
| Formally schooled | 505 | 48.3 |
| Family size | | |
| ≤4 | 347 | 33.2 |
| >4 | 698 | 66.8 |
| [2]Family monthly income (BDT) | | |
| <5000 | 121 | 11.6 |
| 5000-10000 | 469 | 44.9 |
| >10000 | 455 | 43.5 |

| | | |
|---|---|---|
| Current occupation | | |
| Employed | 407 | 39.0 |
| Unemployed/retired | 638 | 61.1 |
| Living arrangement | | |
| Living with family | 992 | 94.9 |
| Living alone | 53 | 5.1 |
| Walking distance to the nearest health centre | | |
| <30 minute | 581 | 55.6 |
| ≥30 minutes | 464 | 44.4 |
| Problems with memory or concentration | | |
| No problem | 676 | 64.7 |
| Low memory or concentration | 369 | 35.3 |
| Suffering from at least one non-communicable chronic condition | | |
| No | 447 | 42.8 |
| Yes | 598 | 57.2 |
| Family members non-responsive | | |
| No | 738 | 70.6 |
| Yes | 307 | 29.4 |
| Feeling of loneliness | | |
| No | 568 | 54.4 |
| Yes | 477 | 45.7 |

[1]Without partner group includes widowed, separated and never married. [2]One USD = 85.75 Bangladeshi Taka (BDT).

**Prevalence of non-disabled frailty**

Figure 1 shows the prevalence of non-disabled frailty, disability, and robustness by age and gender using the FiND questionnaire. We found that around one-fourth (24.8%) of the total participants (n=1045) had non-disabled frailty, while 19.7% were categorized as disabled and 55.5% as robust. No statistically significant differences were observed among participants in terms of age and sex.

**Factors associated with non-disabled frailty**

Table 2 shows the factors associated with non-disabled frailty and disability (with reference to the robust category) in the adjusted multinomial logistic regression model. Although findings for both non-disabled frailty and disability are presented in Table 2, given our research question, we interpret here the findings relevant to non-disabled frailty only. In the adjusted model, compared to the youngest age group (60-69 years), the oldest group (≥80 years) had more than three times higher risk of frailty (RRR= 3.23, 95% CI: 1.41-7.37). Likewise, those living in a larger family with four or more members (RRR= 1.39, 95% CI: 1.01-1.92) and those whose family members were non-responsive to their needs (RRR= 1.47, 95% CI: 1.06-2.03) had 39% and 47% higher risk of being frail, respectively. Participants with low memory and concentration problems had a 56% higher risk of being frail (RRR= 1.56, 95% CI: 1.12-2.17) than those without such problems. Moreover, participants reporting loneliness were significantly more likely to be frail than their counterparts (RRR= 1.45, 95% CI: 1.07-1.98). Sensitivity analysis suggested the results to be robust in quantifying these above associations (Supplementary Table 1).

**Table 2:** Factors associated with non-disabled frailty and disability among the participants (N=1045)

| Characteristics | Disability | | | Frailty | | |
|---|---|---|---|---|---|---|
| | RRR[1] | 95% CI | *P* | RRR | 95% CI | *P* |
| Age (years) | | | | | | |
| 60 – 69 | *Ref* | | | *Ref* | | |
| 70-79 | 2.56 | 1.72-3.80 | <0.001 | 1.08 | 0.72-1.62 | 0.718 |
| ≥ 80 | 10.00 | 4.64-21.56 | <0.001 | 3.23 | 1.41-7.37 | 0.005 |
| Formal schooling | | | | | | |
| No formal schooling | *Ref* | | | *Ref* | | |
| Having formal schooling | 0.75 | 0.53-1.06 | 0.109 | 0.84 | 0.62-1.14 | 0.270 |
| Family size | | | | | | |
| ≤4 | *Ref* | | | *Ref* | | |
| >4 | 1.25 | 0.87-1.80 | 0.235 | 1.39 | 1.01-1.92 | 0.047 |
| Residence | | | | | | |
| Urban | *Ref* | | | *Ref* | | |
| Rural | 0.61 | 0.39-0.96 | 0.032 | 0.69 | 0.46-1.01 | 0.059 |
| Problems with memory or concentration | | | | | | |
| No problem | *Ref* | | | *Ref* | | |
| Low memory or concentration | 2.73 | 1.91-3.91 | <0.001 | 1.56 | 1.12-2.17 | 0.002 |
| Feeling of loneliness | | | | | | |
| No | *Ref* | | | *Ref* | | |

| | | | | | | |
|---|---|---|---|---|---|---|
| Yes | 1.47 | 1.03-2.08 | 0.032 | 1.45 | 1.07-1.98 | 0.017 |
| Family members non-responsive to needs | | | | | | |
| No | *Ref* | | | *Ref* | | |
| Yes | 1.09 | 0.75-1.58 | 0.663 | 1.47 | 1.06-2.03 | 0.020 |

[1]RRR=Relative Risk Ratio; Note: The adjusted model contained all variables in Table 2.

## Discussion

With the aim to examine the prevalence and determinants of non-disabled frailty among Bangladeshi older people, the current study found a 24.8% prevalence of non-disabled frailty and factors such as age, family size, memory or concentration problems, non-responsive family members and loneliness associated with frailty. Our study contributes to the limited literature on frailty among South Asian older adults, specifically from Bangladesh.

Our reported 24.8% prevalence of frailty among older adults is similar to studies in India (26%) [11] and Italy (23.2%) [20]. A systematic review of studies on frailty among community-dwelling older adults reported a pooled prevalence of 10.7% [21]. In contrast, studies from two other South Asian countries, Nepal and Pakistan, have reported a higher prevalence of frailty [10, 12]. In a study from rural Nepal, 65% of participants were frail [10], while in the Pakistani study, 55.4% were frail [12]. The discrepancies in frailty prevalence between the current study and the existing literature may be attributable to several factors, including different measurement tools, heterogeneity in participants' ages, people's living standard, and the burden of NCDs.

In our study, older people aged ≥ 80 were more likely to be frail than their younger counterparts, which aligns with existing literature [22, 23]. Several biological and physiological changes accompanying the ageing process and a high burden of NCDs among older adults explain the increased likelihood of frailty among older people [24]. Like many other countries of the world (e.g., India, China and Indonesia), Bangladesh is also facing an epidemiological transition, resulting in a higher burden of NCDs and multimorbidity, particularly among the older population [25], which might have contributed to a higher level of frailty among the older ages. Additionally, frailty was found to be associated with cognitive impairment, such as having problems with memory and concentration, in our and previous studies [22, 23], which may also add to the burden among older adults.

Consistent with the existing literature [26], we found that older adults living in a large family and reporting their family members as non-responsive to their care had a higher likelihood of frailty. Several contextual factors together explain the observed findings between family and frailty. The social welfare systems for older people are non-existential in Bangladesh. Thus, at an older age, many Bangladeshi people financially, emotionally, and physically depend on adult children and receive care from them. The adult son, who is usually the sole provider for the family, has to meet the competing needs of all family members. In many Bangladeshi families that struggle to arrange the bread and butter, the demand for competing needs from many family members may result in sub-optimal care for the older family members [27]. Consequently, older adults may feel that the family members are non-responsive to their needs. The financial constraints could also be a barrier to good nutrition and access to health care for older parents [28]. All these issues can compromise

older people's quality of life and make them vulnerable to frailty and other adverse health outcomes [29, 30].

Like other studies [19, 31], this study also found an association between frailty and loneliness. Evidence suggests that high levels of loneliness result in declined mobility and increased difficulties with daily living activities [31]. Lonely people are more likely to be inactive [19], which increases the risk of physical frailty [19, 31]. We acknowledge that in Bangladesh, where multigenerational households are common,  living alone is not typical. Culturally, in Bangladesh, older people are attached to families, living with children and grandchildren [32]. Although living in a large multigenerational family offers social support, loneliness may be partly due to losing a spouse and friends, which is common in old age. Second, out-migration is very high in Bangladesh [33]. Even in a multigenerational family setting, a void is left by migrant children. Evidence suggests that the left-behind parents suffer the emotional cost of migration  [34]. For some, loneliness may also be created due to neglect from family members which was more evident in our study as about 30% reported their family members were not responsive to their needs. As discussed in the previous paragraph, the financial constraint and demand to meet competing needs of multiple family members may result in sub-optimal care for the older family members and thus a feeling of neglect [27]. Recently, change in family dynamics is also noted in Bangladesh where nuclear family is being more popular and in some cases, older adults live with paid maids, separated from their children [32]. Older people's limited social support from their children/family members and being separated from their children increase the likelihood of depression, loneliness, and cognitive decline [35], all of which can contribute to developing frailty.

## Implications for policy and practice

Due to the demographic transition resulting in a burgeoning number of older adults in Bangladesh [2], the prevalence of frailty is projected to increase. Health and social care services are meager for the older population in Bangladesh, and the existing family care is challenged. The Government of Bangladesh has introduced National Policy on Ageing (2007), the Parents Care Act (2013), and Old Age Allowance (OAA) program [36], but on a limited scale. There is a lack of a national policy or strategy focusing on old age health and social care issues in Bangladesh, which is critical to addressing frailty and disability. Due to the absence of healthcare insurance support and resulting high out-of-pocket costs, preventive services are underutilized [3]. Therefore, introducing health insurance could be crucial to access preventive services and ensure early diagnosis, treatment and delayed progression of frailty. Providing comprehensive supportive care, including multidisciplinary care services, individualized care, and long-term follow-up, would be particularly important to address frailty. Strengthening primary care services, including promoting physical activities and ensuring proper nutrition and health education, are also proven effective in managing frailty.

## Strengths and limitations of the study

This study's generalizability is one of its strengths, as it collected data from all eight divisions of Bangladesh. Other notable methodological strengths include the large sample size, using a standard tool to measure outcomes, and data collection by trained and experienced surveyors. However, the cross-sectional design limits the ability to establish a causal association. Due to the paucity of literature on frailty in Bangladesh, the current study intended to quantify the burden and serve as a bas[illegible]e. We recommend a longitudinal design in the future to better establish the

predictors for frailty in this population. Although we used a validated scale for measuring frailty, it was not explicitly validated among Bangladeshi older adults. Thus, future studies also have an opportunity to validate the FiND questionnaire in Bangladesh. Given the self-reported nature of the survey, we also anticipate measurement bias in our study. Finally, our study is limited to quantitative analysis, which helped us to understand the potential association. However, to better understand the underlying reasons for these associations, specifically the observed relationships between family and frailty, we recommend a future qualitative study.

## Conclusion

The present study highlighted that around one in four older adults had non-disabled frailty in Bangladesh. Specifically, those relatively older, living in large families, suffering from cognitive problems or loneliness, and not properly cared for by their family members were more vulnerable to frailty. The findings of the study have significant implications for policy and practice. The findings suggest the need for developing interventions targeting the at-risk groups to address the high burden of frailty. Providing long-term care and relevant health promotion services may effectively prevent frailty and associated long-term adverse health outcomes.

## Acknowledgements

We acknowledge the role of Sadia Sumaia Chowdhury, Programme Manager, ARCED Foundation and Md. Zahirul Islam, Project Associate, ARCED Foundation, for their support in data collection for the study.

## Contributors

**Sabuj Kanti Mistry**: Conceptualization, Methodology, Investigation, Software, Data curation, Formal analysis, Supervision, Writing- Original draft preparation. **ARM Mehrab Ali**: Conceptualization, Methodology, Investigation, Supervision, Writing- Original draft preparation. **Uday Narayan Yadav**: Conceptualization, Methodology, Writing- Original draft preparation. **Saruna Ghimire**: Writing- Original draft preparation. **Afsana Anwar**: Writing- Original draft preparation. **Md. Nazmul Huda**: Writing- Original draft preparation. **Fouzia Khanam**: Writing- Original draft preparation. **Rashidul Alam Mahumud**: Writing- Original draft preparation. **Ateeb Ahmad Parray**: Writing- Original draft preparation. **Shovon Bhattacharjee**: Writing- Original draft preparation. **David Lim**: Writing- Reviewing and Editing. **Mark Fort Harris**: Writing- Reviewing and Editing.

## Funding

This research did not receive any external funding.

## Competing interest

The authors declare that they have no competing interest to disclose.

## Availability of data and materials

The datasets generated and/or analyzed during the current study are available as supporting file to this manuscript (Supplementary file 2).

# References


1. World Health Organization. Ageing and health 2022 [18/12/2022]. Available from: https://www.who.int/news-room/fact-sheets/detail/ageing-and-health.
2. Kabir R, Khan HT, Kabir M, Rahman MTJESJ. Population ageing in Bangladesh and its implication on health care. 2013;9(33):34-47.
3. Mahumud RA, Sultana M, Kundu S, Rahman MA, Mistry SK, Kamara JK, et al. The burden of chronic diseases and patients' preference for healthcare services among adult patients suffering from chronic diseases in Bangladesh. Health Expectations. 2022.
4. Matthews NR, Porter GJ, Varghese M, Sapkota N, Khan MM, Lukose A, et al. Health and socioeconomic resource provision for older people in South Asian countries: Bangladesh, India, Nepal, Pakistan and Sri Lanka. 2020.
5. Yadav UN, Tamang MK, Thapa TB, Hosseinzadeh H, Harris MF, Yadav KK. Prevalence and determinants of frailty in the absence of disability among older population: a cross sectional study from rural communities in Nepal. BMC geriatrics. 2019;19(1):1-7.
6. Fried LP, Tangen CM, Walston J, Newman AB, Hirsch C, Gottdiener J, et al. Frailty in older adults: evidence for a phenotype. 2001;56(3):M146-M57.
7. Rogers NT, Marshall A, Roberts CH, Demakakos P, Steptoe A, Scholes SJPo. Physical activity and trajectories of frailty among older adults: Evidence from the English Longitudinal Study of Ageing. 2017;12(2):e0170878.
8. Khezrian M, Myint PK, McNeil C, Murray ADJG. A review of frailty syndrome and its physical, cognitive and emotional domains in the elderly. 2017;2(4):36.
9. Siriwardhana DD, Hardoon S, Rait G, Weerasinghe MC, Walters KR. Prevalence of frailty and prefrailty among community-dwelling older adults in low-income and middle-income countries: a systematic review and meta-analysis. BMJ open. 2018;8(3):e018195.
10. Yadav UN, Tamang MK, Thapa TB, Hosseinzadeh H, Harris MF, Yadav KKJBg. Prevalence and determinants of frailty in the absence of disability among older population: a cross sectional study from rural communities in Nepal. 2019;19(1):1-7.
11. Kashikar Y, Nagarkar AJIJoG. Prevalence and Determinants of Frailty in Older Adults in India. 2016;30(3).
12. Ali AA, Haq N, Chang K, Naqi S, Rafique M, Ismail M, et al. Impact of obesity on frailty in older population of Karachi, Pakistan. Advances in Medical, Dental and Health Sciences. 2019;2(4).
13. Siriwardhana DD, Weerasinghe MC, Rait G, Falcaro M, Scholes S, Walters KR. Prevalence of frailty in rural community-dwelling older adults in Kegalle district of Sri Lanka: a population-based cross-sectional study. BMJ open. 2019;9(1):e026314.
14. Rahman MM, Hamiduzzaman M, Akter M, Farhana Z, Hossain MK, Hasan MN, et al. Frailty indexed classification of Bangladeshi older adults' physio-psychosocial health and associated risk factors-a cross-sectional survey study. BMC geriatrics. 2021;21(1):1-10.
15. Mistry SK, Ali AM, Rahman MA, Yadav UN, Gupta B, Rahman MA, et al. Changes in tobacco use patterns during COVID-19 and their correlates among older adults in Bangladesh. International Journal of Environmental Research and Public Health. 2021;18(4):1779.
16. Mistry SK, Ali ARMM, Yadav UN, Huda MN, Ghimire S, Saha M, et al. Loneliness and its correlates among Bangladeshi older adults during the COVID-19 pandemic. Scientific Reports. 2022;12(1):15020.
17. BBS. Elderly Population in Bangladesh: Current features and future perspectives. Dhaka: Banglaedsh Bureau of Statistics, Ministry of Bangladesh, Government of Bangladesh, 2015.
18. Hughes ME, Waite LJ, Hawkley LC, Cacioppo JT. A short scale for measuring loneliness in large surveys: Results from two population-based studies. Research on aging. 2004;26(6):655-72.

19. Perissinotto CM, Cenzer IS, Covinsky KE. Loneliness in older persons: a predictor of functional decline and death. Archives of internal medicine. 2012;172(14):1078-84.
20. Battaggia A, Scalisi A, Novelletto BF, Fusello M, Michieli R, Cancian M. Prevalence of frailty in older people in Veneto (Italy). Journal of drug assessment. 2019;8(1):1-12.
21. Collard RM, Boter H, Schoevers RA, Oude Voshaar RC. Prevalence of frailty in community-dwelling older persons: a systematic review. Journal of the american geriatrics society. 2012;60(8):1487-92.
22. Curcio C-L, Henao G-M, Gomez F. Frailty among rural elderly adults. BMC geriatrics. 2014;14(1):1-9.
23. de Albuquerque Sousa ACP, Dias RC, Maciel ÁCC, Guerra RO. Frailty syndrome and associated factors in community-dwelling elderly in Northeast Brazil. Archives of gerontology and geriatrics. 2012;54(2):e95-e101.
24. Dziechciaz M, Filip R. Biological psychological and social determinants of old age: Bio-psycho-social aspects of human aging. Annals of Agricultural and Environmental Medicine. 2014;21(4).
25. Biswas T, Islam MS, Linton N, Rawal LB. Socio-economic inequality of chronic non-communicable diseases in Bangladesh. PloS one. 2016;11(11):e0167140.
26. Rahman MM, Hamiduzzaman M, Akter M, Farhana Z, Hossain MK, Hasan MN, et al. Frailty indexed classification of Bangladeshi older adults' physio-psychosocial health and associated risk factors-a cross-sectional survey study. BMC geriatrics. 2021;21(1):1-10.
27. Ali I, Azman A, Ahmed MK, Akhtar T, Deceukelier B. Assessment of the Needs of Rural Older People in Bangladesh. The International Journal of Community and Social Development. 2022;4(3):317-38.
28. Nortey ST, Aryeetey GC, Aikins M, Amendah D, Nonvignon J. Economic burden of family caregiving for elderly population in southern Ghana: the case of a peri-urban district. International journal for equity in health. 2017;16(1):1-9.
29. Kojima G, Liljas AEM, Iliffe S. Frailty syndrome: implications and challenges for health care policy. Risk management and healthcare policy. 2019:23-30.
30. Rosenberg T, Montgomery P, Hay V, Lattimer R. Using frailty and quality of life measures in clinical care of the elderly in Canada to predict death, nursing home transfer and hospitalisation-the frailty and ageing cohort study. BMJ open. 2019;9(11):e032712.
31. Perissinotto CM, Cenzer IS, Covinsky KEJAoim. Loneliness in older persons: a predictor of functional decline and death. 2012;172(14):1078-84.
32. Hossain MI, Akhtar T, Uddin MT. The elderly care services and their current situation in Bangladesh: An understanding from theoretical perspective. Journal of Medical Sciences. 2006;6(2):131-8.
33. Alam MZ, Mamun AA. Dynamics of internal migration in Bangladesh: Trends, patterns, determinants, and causes. Plos one. 2022;17(2):e0263878.
34. Kaur KJ, Sinha AK. Empty Nests: A Study on the Left-Behind Parents of Emigrated Children from Punjab (India). The Family Journal. 2023:10664807231157028.
35. Mehrabi F, Beland F. Frailty as a Moderator of the Relationship between Social Isolation and Health Outcomes in Community-Dwelling Older Adults. Int J Environ Res Public Health. 2021;18(4). Epub 2021/02/13. doi: 10.3390/ijerph18041675. PubMed PMID: 33572443; PubMed Central PMCID: PMCPMC7916171.
36. Begum A, Islam R. An Inclusive Approach to Care of the Elderly in Bangladesh. Dhaka: Bangladesh Institute of Development Studies, 2019.
37. Global Issues. Mental Health Strategic Plan for Bangladesh: An Overview 2022 [11/11/22]. Available from:

https://www.globalissues.org/news/2021/10/28/29200#:~:text=The%20National%20Mental%20Health%20Strategy,psychosocial%20wellbeing%20services%20and%20information.

## Figure title

**Figure 1:** Prevalence of non-disabled frailty by age and gender (n=1045)

## Supplementary materials

**Supplementary File 1:** Factors associated with non-disabled frailty and disability among the participants (N=1045)

**Supplementary File 2:** Dataset used for the analysis in the current study.

Click here to access/download;Figure;Figure 1_R1.docx

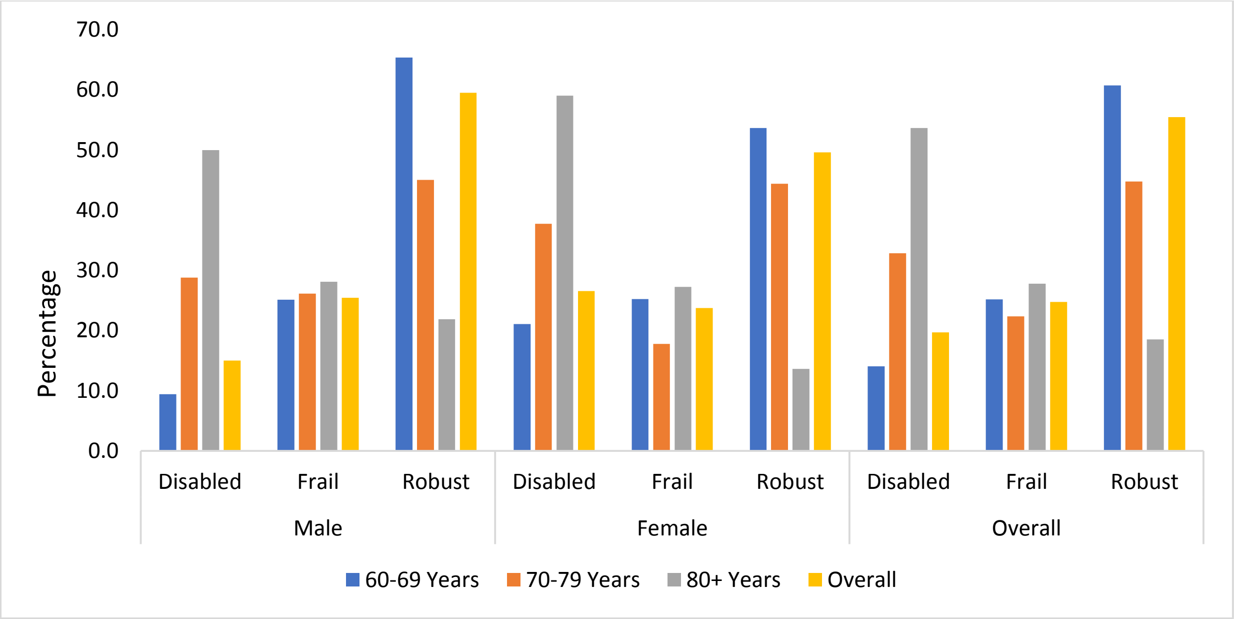


Figure 1: Prevalence of non-disabled frailty by age and gender (n=1045)

Click here to access/download
**Supporting Information**
Supplementary file 1_R1.docx

Click here to access/download
**Supporting Information**
Supplementary file 2_R1.dta

Revised Manuscript with Track Changes

# The burden of non-disabled frailty and its associated factors among older adults in Bangladesh

Sabuj Kanti Mistry[1,2,3], ARM Mehrab Ali[1], Uday Narayan Yadav[2,4], Saruna Ghimire[5], Afsana Anwar[6], Md. Nazmul Huda[1,7], Fouzia Khanam[8], Rashidul Alam Mahumud[9], Ateeb Ahmad Parray[10], Shovon Bhattacharjee[11], David Lim[7,12], Mark Fort Harris[2]

[1] ARCED Foundation, 13/1 Pallabi, Mirpur-12, Dhaka 1216, Bangladesh

[2] ~~Centre for Primary Health Care and Equity~~School of Population Health, University of New South Wales, Sydney, Australia

[3] Department of Public Health, Daffodil International University, Dhaka 1207, Bangladesh

[4] National Centre for Epidemiology and Population Health, The Australian National University, Canberra, ACT, Australia

[5] Department of Sociology and Gerontology and Scripps Gerontology Center, Miami University, Oxford, OH, USA

[6] Health and Nutrition, Social Assistance and Rehabilitation for the Physically Vulnerable (SARPV), SARPV Complex, ~~Cox's~~ Cox's Bazar 4700, Bangladesh

[7] Translational Health Research Institute, Western Sydney University, Campbeltown, NSW 2560, Australia

[8] Department of Public Health, North South University, Dhaka, Bangladesh

[9] NHMRC Clinical Trials Centre, Faculty of Medicine and Health, The University of Sydney, Camperdown, NSW 2006, Australia

[10] Department of International Health, Johns Hopkins Bloomberg School of Public Health, Johns Hopkins University, Baltimore, MD 21218, United States

[11] Biosecurity Program, The Kirby Institute, University of New South Wales, Sydney, Australia.

[12] School of Health Sciences, Western Sydney University, Campbeltown, NSW 2560, Australia

* Correspondence to: Sabuj Kanti Mistry, ARCED Foundation, 13/1 Pallabi, Mirpur-12, Dhaka, Bangladesh, Mobile: +8801716411922, E-mail: smitra411@gmail.com

**Short title:** Frailty among older adults in Bangladesh

# Abstract

**Objective**

The present study aim present study s to measured the prevalence of non-disabled frailty and its associated factors among Bangladeshi older adults.

**Methods**

This cross-sectional study was conducted in September 2021 among 1,045 Bangladeshi older adults (≥60 years). Telephone interviews, were conducted using a semi-structured questionnaire, to collected data on participants' participants' characteristics and levels of frailty. The non-disabled frailty was measured using the ''Frail Non-Disabled (FiND)'' questionnaire. A multinomial logistic regression model assessed the factors associated with frailty among the participants.

**Results**

One in fourAround onea- quarter of the participants (24.8%) had were frail a non-disabled frailty. The prevalence of frailty was relatively higher among males than females (25.5% versus 23.8%), although the difference was statistically not significant (0.527). The multinomial Rregression analysis revealed showed that older participants who belonged to the oldest age group i.e., aged 80 years and above(≥ 80 years) (RRR= 3.23, 95% CI: 1.41-7.37), those who belonged towere from aliving in a large family with family of ≥4 people members (RRR= 1.39, 95% CI: 1.01-1.92), who hadhaving memory and or concentration problems (RRR= 1.56, 95% CI: 1.12-2.17), who had family members being non-responsive to their needs (RRR= 1.47, 95% CI: 1.06-2.03) and who

~~reported a feeling of~~feeling lonely~~iness~~ (RRR= 1.45, 95% CI: 1.07-1.98) ~~were found to had a higher risk of being~~~~had higher odds~~were more likely to be frail ~~of frailty compared~~than ~~to~~ their counterparts.

**Conclusions**

The ~~findings of the present study~~present study's findings suggest ~~providing comprehensive supportive care package for the older adults and strengthening primary care services including promotion of exercise, ensuring proper nutrition and health education.~~ providing long-term care tailored towards frail older adults. Specifically, ~~for this vulnerable population, particularly emphasizing~~ those ~~who are~~ relatively ~~aged~~older, ~~belong~~living in ~~to a~~ large famil~~y~~ies, suffering from cognitive problems or loneliness, and ~~who are~~ not properly cared for by their family members should be targeted for relevant health promotion interventions.

**Keywords:** non-disabled frailty; older adults; Bangladesh

## Introduction

The world is rapidly transitioning into an ageing society. According to the World Health Organization (WHO), ~~By 2030,~~ one in six individuals worldwide will be 60 years or older by 2030, and that proportion will quadruple by 2050~~,~~ ~~according to the World Health Organization (WHO)~~ ~~According to the World Health Organization (WHO), one in six individuals worldwide will be 60 years or older by 2030, and that proportion will quadruple by 2050~~ [1]. Low- and middle-income countries (LMICs) are projected to have two-thirds of their population aged 60 or older by 2050 ~~In particular, it is projected that two-thirds of the population in low- and middle-income countries (LMICs) will be 60 years or older by 2050~~ [1]. In Bangladesh, reflecting this global demographic shift, the total number of older adults aged 60 years and above is expected to increase from 8 million to 44 million by 2050~~Consistent with the global demographic shift, the total number of older adults aged 60 years and above will increase from 8 million to 44 million by 2050 in Bangladesh~~ [2]. This demographic change has resulted in ~~also led to~~ an increased burden of conditions common in older age, including various non-communicable diseases (NCDs) and frailty ~~The recent demographic shift has also resulted in an increased burden of non-communicable diseases (NCDs) worldwide including in Bangladesh~~ [3]; the latter~~, .~~ ~~including frailty, which~~ is now recognized as an emerging concern among older adults in South Asian countries, including Bangladesh ~~Consequently, frailty is increasingly recognized as an emerging concern among older adults in South Asian countries, including Bangladesh~~ [4].

Frailty is a condition characterized by low physical activity, cognitive performance, and nutritional status. ~~It increases the risk of disability progression and mortality among older adultsFrailty is a condition associated with low physical activity, cognitive performance and nutritional status that~~

**Formatted:** Font: 12 pt

increases the risk of disability progression and mortality among older adults [5]. Fried and colleagues The operational definition of frailty was provided by Fried and colleagues [6], provided the operational definition of frailty, which states that frailty is a condition where three out of five phenotypic criteria are met: shrinkage (weight loss), exhaustion, weaknesses, low gait speed, and low physical activity. Frailty predicts many long-term health outcomes, disability and mortality [5] and interferes with one's Frailty is associated with predicting long-term health outcomes and the ability to live independently while maintaining a high quality of life stating that frailty is a condition meeting three out of five phenotypic criteria named: shrinkage (weight loss), exhaustion, weaknesses, low gait speed, and low physical activity. A person's frailty can predict long-term health outcomes and the ability to live independently and maintain a high quality of life [7]. Frailty is a common occurrencecommon in older age due to biological senescencethe deterioration of multiple systems, that encompassresults in the deterioration ofing physical, cognitive, and psychological functions [8]. As individuals grow older, these systems naturally experience decline and become more vulnerable, leading to the development of frailty. This condition manifests as a state of increased vulnerability and decreased resilience, resulting in decreased physical activity, diminished cognitive performance, and compromised nutritional status [7]. The cumulative impact of these deteriorations contributes to the prevalence of frailty among older adults. Given the deterioration of function associated with multiple systems, including physical, cognitive, and psychological functions, frailty in old age is common [8].

There is evidence indicating a high burden of frailty in LMICs. A systematic review and meta-analysis conducted with 56 studies in LMICs documented that the prevalence of frailty and

**Formatted:** Font: 12 pt

prefrailty among community-–dwelling older adults was 17.4% and 49.3%, respectivelyEvidence indicates a high burden of frailty in LMICs. A systematic review and meta-analysis conducted using 56 studies carried out in LMICs documented that the prevalence of frailty and prefrailty was 17.4% and was 49.3%, respectively among the community dwelling older adults [9]. The prevalence of frailty was found to be higher among older adults from South Asian countries. For instance, a Nepalese study reported that 65% of participants had frailty without disabilityThe prevalence of frailty was also reported to be higher among the older adults from the South Asian countries. For example, a Nepalese study showed that 65% of participants had frailty without disability [10]. In India, 26% of older adults were frail, while 63.6% were pre-frail, and only 10.4% were non-frail Evidence from India documented that 26% of older adults were frail; 63.6% were pre-frail, and only 10.4% were non-frail [11]. Studies conducted in Pakistan [12] and Sri Lanka [13] found that 55.4% and 15.2% of the older adults were frail, with 44.6% and 48.5% being pre-frail, respectively. These studies [10, 11, 13] also identified several determinants of frailty among the older adults, such as age, education, occupation, out-of-pocket health care cost, depression, and lack of physical activities, suffering from depression, and lack of care from family members.

However, there is Llimited -evidence on the prevalence and determinants of frailty among older adults in Bangladeshexists on the prevalence and determinants of frailty among older adults in Bangladesh. The only reported study on frailty among Bangladeshi older adults showed that 61.6% of the participants had moderate to severe levels of frailty assessed using the 30-indicator Frailty Index [14]. However, this study was conducted in a small geographical area of the country and used the 30-indicator Frailty Indexa scale that does not measure non-disabled frailtyYet, that study was limited to a small geographical area of the country and used a scale that does not measure non-

**Formatted:** Font: 12 pt

**Formatted:** Highlight

**Formatted:** Font: 12 pt

disabled frailty. Considering the high burden of -NCDs coupled with limited healthcare provision in Bangladesh, we anticipate a high prevalence of frailty.Given the high burden of non-communicable diseases (NCDs) coupled with limited healthcare provision in Bangladesh, we anticipate frailty to be highly prevalent. Therefore, further research is needed to investigate this topic in the Bangladeshi context. Accordingly, the present study aims to quantify the burden of frailty and identify associated factors among Bangladeshi older adults.Thus, there is a need to study this topic further in the Bangladeshi context. Thus, the present study aims to quantify the burden of frailty and identify its associated factors among Bangladeshi older adults.

Formatted: Font: 12 pt

Formatted: Font: 12 pt

# Materials and methods

## Study design and participants

This cross-sectional study was conducted in September 2021. Previously, between 2016–2020, the ARCED Foundation conducted ten different community-based studies and established a registry using demographic information from these studies, which served as the sampling frame for the current study. Notably, The sampling frame was drawn from a pre-established registry, developed by merging the contact information of households participating infrom ten different community-based studies conducted by the ARCED Foundation to explore different social issues during 2016-2020. Tthis sampling frame - that coveredincluded households from all eight administrative divisions of Bangladesh and was previously used [15] [15, 16]. Considering a 50% unknown prevalence with a 5% margin of error, at the 95% confidence level, and 90% power of the testpower, , and 95% response rate, a sample size of 1096 was requiredcalculated. However, only 1045 of 1096 the approached 1096 eligible participants responded to the study with resulting

in an overall response rate of approximately 95%. Based on the population distribution of older people by geography in Bangladesh, we adopted a probability proportionate to size (of the eight administrative divisions) approach to ~~select~~ determine the size of older people in each division [17]. The inclusion criterion was the minimum age of 60 years of the participants. Exclusion criteria were suffering from any adverse mental conditions i.e., clinically diagnosed schizophrenia, bipolar mood disorder, dementia/cognitive impairment, and inability to communicate.

## Measures

*Outcome measure*

The ~~study's primary~~ outcome of ~~this study~~interest was non-disabled frailty, measured using the ~~'~~'Frail Non-Disabled (FiND) ~~questionnaire'~~ questionnaire' [18]. The FiND questionnaire is~~,~~ designed to measure non-disabled frailty among the ~~the~~ older population ~~and community dwellers~~ and ~~has been~~was previously used among ~~people~~ older adults in Nepal in~~of from~~ a similar setting~~s~~ ~~in Nepal~~ [5]. The FiND questionnaire contains five ~~sections~~questions under two sections. The first two questions on mobility (i.e., items A and B) belong to the disability section, and the latter three on weight loss, exhaustion, and physical activity (i.e., items C, D and E) belong to the frailty section. ~~: sections A and B measure mobility~~ and ~~disability, section C measure weight loss, section D measure exhaustion, and section E measure the level of physical activity.~~ Each five items are coded as 0 and 1, indicating negative and affirmative responses, respectively. Participants ~~were~~ are categorized into one of three categories, i.e., "disabled," "frail," and "robust." If a participant responds affirmatively to at least one of the two disability items (i.e., item $A + B \geq 1$), they are classified as disabled. Frailty is when a participant responds negatively to both disability questions but affirmatively to at least one frailty item (i.e., $A + B = 0$ and $C + D + E \geq 1$). A participant is

classified as ~~as “disabled” if item A + B ≥ 1, “frail” if A + B = 0 and C + D + E ≥ 1, and “~~“robust” when the sum of five items is zero, i.e., indicating the ~~i.e.,~~ absence of both frailty or disability~~”~~ .”~~if A + B + C + D + E = 0.~~ A Cronbach α of 0.75 in the current study indicated the scale is reliable among our study population.

*Explanatory variables*

An extensive literature review guided the selection of explanatory variables [10-13, 15]. Explanatory variables considered in this study were administrative divisions (Barishal, Chattogram, Dhaka, Mymensingh, Khulna, Rajshahi, Rangpur, Sylhet), age (categorized as 60-69, 70-79, and ≥80), sex (male/female), marital status (married and~~/~~ without a partner; latter included ~~i.e.,~~ widowed, separated and never married), formal schooling (with and without formal schooling), family size (≤4 or >4), family monthly income in Bangladeshi Taka (BDT) (<5,000, 5,000-10,000, >10,000), residence (urban/rural), current occupation (employed,~~/~~ unemployed or retired), living arrangement (living alone or with family), walking distance to the nearest health center (<30 min/≥30 min), memory or concentration problems (no problem/low memory or concentration), suffering from at least one non-communicable chronic conditions (yes/no), a perception that family members are non-responsive to their needs (yes/no), and feeling of loneliness (yes/no).

Self-reported information on the participant’s ~~suffering~~ ever diagnosis of ~~from any~~nine non-communicable chronic conditions (i.e., arthritis, hypertension, heart diseases, stroke, hypercholesterolemia, diabetes, chronic respiratory diseases, chronic kidney disease, ~~or~~ and cancer) was collected as dichotomized ~~. This was categorized as~~ yes/no responses.

To explore if participants had any memory or concentration problems, we asked the following question: ~~"~~‘‘Do you have any memory (remembering things properly) or concentration (~~could~~focusing/concentrating ~~concentrate~~ properly while doing any action) problem?~~"~~’’

Loneliness was measured using the 3-item UCLA Loneliness scale [19]. Each item on the scale is measured as a yes/no question. The three items included: how often do you feel: (i) lack ~~of~~ companionship, (ii) left out, and (iii) isolated in the last two weeks? Each item in the scale is measured in terms of 3-item Likert responses: hardly ever (1 point), some of the time (2 points), and often (3 points). Participants were classified as lonely if they answered ~~"~~‘‘some of the ~~time'~~ time’ or ~~"~~‘‘often~~"~~’ to any of the three items [19].

**Data collection tools and techniques**

Telephone interviews ~~using~~ were conducted using a pre-tested semi-structured questionnaire, and data were ~~collected the information~~collected~~ion was accomplished~~ electronically using SurveyCTO mobile app (https://www.surveycto.com/). ~~Trained~~ Ten research assistants were recruited as surveyors based on their previous experiences administering health surveys on the electronic platform. The research assistants were trained extensively via Zoom meetings for three full days by the coauthors (SKM, AMA, UNY) before beginning the data collection ~~through Zoom meetings~~.

The English version of the questionnaire was first translated into Bengali and then ~~back~~ back-translated to English by two researchers (SKM and AMA) to ensure the ~~content's~~ content’s

construct and face validity. The questionnaire was then piloted among a small sample (n=10) of older people adults for cultural validation of contents and refinement of the questions. The piloted response, excluded from data analyses, who were not sampled to refine the questions and for cultural validation in the final version. The piloted Bengali version of the tool did not receive provide any corrections/suggestions and thus was used as is for the data collection. from the participants on the contents. The interviews were conducted by trained surveyors using this questionnaire and eEach interview took around half an hour.

**Statistical analyses**

The distribution of the variables was assessed through descriptive analysesDescriptive analyseis, i.e., frequency and percentage, waswere performed to explore the distribution of the sociodemographic characteristics of the respondents. To explore the factors independently associated with non-disabled frailty, We used a multinomial logistic regression models were run, adjusting for important confounders model to explore the factors independently associated with non-disabled frailty. Covariates for adjustment were identified using the backward elimination method based on the Akaike information criterion (AIC). The initial model was run withn with including all potential covariates in the regression modellisted in Table 1, and only age, formal schooling, family size, residence, problems with memory or concentration, loneliness, and non-responsive family members were retained/adjusted in the final model. . Then, the variables for the final model were selected using backward elimination with the Akaike information criterion (AIC), variables for the final model were selected. The final model was executed including these final sets of variables.and executed. The final model was also tested for sensitivity using the bootstrapping approach by resampling observations with 10,000 replications. The Relative Risk

Ratio (RRR) and associated 95% confidence interval (95% CI) for the “disabled” and “frail” categories are provided with reference to the “robust” category in Table 3. ~~The Relative Risk Ratio (RRR) and associated 95% confidence interval (95% CI) are reported.~~ All analyses were performed using the statistical software package Stata (Version 14.0).

**Ethics approval**

The study protocol was approved by the Institutional Review Board of the Institute of Health Economics, the University of Dhaka, Bangladesh (Ref: IHE/2020/1037). ~~All procedures performed in studies involving human participants were in accordance with the ethical standards of the institutional and/or national research committee and with the 1964 Helsinki Declaration and its later amendments or comparable ethical standards.~~ Verbal informed consent was sought from the participants before administering the survey.

**Patient and public involvement**

Patients and/or the public were not involved in developing~~development of~~ research questions, ~~study~~ designing and ~~,~~ conducting ~~s~~the study ~~and result dissemination~~, and disseminating the results.

## Results

**Characteristics of the participants**

The sociodemographic characteristics of the participants are presented in Table 1. The majority of the participants were aged 60-69 years (75.6%), males (59.3%), and married (76.5%). More than half of the participants (51.7%) had no formal schooling, 66.8% belonged to a large family of more than four members, 82.6% lived in rural areas, and 61.1% were unemployed or retired during the

survey period. Furthermore, 35.3% had problems with memory or concentration, 57.2% suffered from at least one non-communicable chronic condition, 29.4% had their family members non-responsive to their care, and 45.7% felt lonely (Table 1). The sociodemographic characteristics of the participants are presented in Table 1. Table 1 summarizes the characteristics of 1045 study participants, where 19.0% were from the Khulna Division, The majority of the participants 75.6% were aged 60-69 years (75.6%), 59.3% were males (59.3%), and 76.5% were married (76.5%). Over More than half of the participants (51.7%) had no formal schooling, 44.9% had a monthly family income range of 5000 – 10000 BDT, 66.8% had belonged to a large family size withof more than four members, 82.6% lived in rural areas, and 61.1% were either unemployed or retired during the survey period. Around 95% of the study participants lived with their family membersAlso, 35.3% suffered had problems with memory or concentration, and 57.2% suffered were suffering from at least one non-communicable chronic condition, . Nearly one-third of the participants (29.4%) reported thathad their family members were not responsive to their care, and . In addition, approximately half of the participants (45.7%) felt were feeling lonelyloneliness.

Formatted: Highlight

**Table 1:** Characteristics of study participants (N=1045)

| Characteristics | N | % |
|---|---|---|
| Administrative division | | |
| Barishal | 146 | 14.0 |
| Chattogram | 98 | 9.4 |
| Dhaka | 172 | 16.5 |
| Mymensingh | 69 | 6.6 |
| Khulna | 198 | 19.0 |

| | | |
|---|---|---|
| Rajshahi | 145 | 13.9 |
| Rangpur | 161 | 15.4 |
| Sylhet | 56 | 5.4 |
| Residence | | |
| Urban | 182 | 17.4 |
| Rural | 863 | 82.6 |
| Age (in years) | | |
| 60 – 69 | 790 | 75.6 |
| 70-79 | 201 | 19.2 |
| ≥ 80 | 54 | 5.2 |
| Sex | | |
| Male | 620 | 59.3 |
| Female | 425 | 40.7 |
| Marital status | | |
| Married | 799 | 76.5 |
| [1]Without partner | 246 | 23.5 |
| Formal schooling | | |
| No formal schooling | 540 | 51.7 |
| Formally schooled | 505 | 48.3 |
| Family size | | |
| ≤4 | 347 | 33.2 |
| >4 | 698 | 66.8 |
| [2]Family monthly income (BDT) | | |

| | | |
|---|---|---|
| <5000 | 121 | 11.6 |
| 5000-10000 | 469 | 44.9 |
| >10000 | 455 | 43.5 |
| Current occupation | | |
| Employed | 407 | 39.0 |
| Unemployed/retired | 638 | 61.1 |
| Living arrangement | | |
| Living with family | 992 | 94.9 |
| Living alone | 53 | 5.1 |
| Walking distance to the nearest health centre | | |
| <30 minute | 581 | 55.6 |
| ≥30 minutes | 464 | 44.4 |
| Problems with memory or concentration | | |
| No problem | 676 | 64.7 |
| Low memory or concentration | 369 | 35.3 |
| Suffering from at least one non-communicable chronic condition | | |
| No | 447 | 42.8 |
| Yes | 598 | 57.2 |
| Family members non-responsive | | |
| No | 738 | 70.6 |
| Yes | 307 | 29.4 |
| Feeling of loneliness | | |
| No | 568 | 54.4 |

| | | |
|---|---|---|
| Yes | 477 | 45.7 |

[1]Without partner group includes~~s those~~ widowed, separated and never married. [2]One USD = 85.75 Bangladeshi Taka (BDT).

**Prevalence of non-disabled frailty**

Figure 1 shows the prevalence of non-disabled frailty, disability, and robustness by age and gender using the FiND questionnaire. We found that around one-fourth (24.8%) of the total participants (n=1045) had ~~a~~ non-disabled frailty, while 19.7% were ~~considered~~ categorized as disabled~~,~~ and 55.5% ~~were classified~~ as robust. ~~Notably, the prevalence of frailty was relatively higher among males than females (25.5% versus 23.8%), although the difference was statistically non-significant.~~ No statistically ~~real~~ significant differences ~~was~~ were observed among participants ~~from different~~in terms of age ~~groups~~and sex.

**Factors associated with non-disabled frailty**

Table 2 shows the factors ~~independently~~ associated with non-disabled frailty and disability (with reference to the robust category) in the adjusted multinomial logistic regression model. Although findings for both non-disabled frailty and disability are presented in Table 2, given our research question, ~~However,~~ we interpret here the ~~focused on~~findings relevant to non-disabled frailty only. ~~while interpreting the result in line with the objective of the present research. In the regression analysis it was revealed that,~~In the adjusted model, ~~-~~compared to the youngest age group (60-69 years), the oldest group (≥80 years) had more than three times higher risk of frailty (RRR= 3.23, 95% CI: 1.41-7.37). Likewise, those living in a larger family with four or more ~~people~~ members (RRR= 1.39, 95% CI: 1.01-1.92) and those whose ~~who perceiving had~~ family members were ~~to be~~

non-responsive to their needs (RRR= 1.47, 95% CI: 1.06-2.03) had 39% and 47% higher risk of being frail, respectively. Participants with low memory and concentration problems had a 56% higher risk of being frail (RRR= 1.56, 95% CI: 1.12-2.17) than those without such problems. Moreover, participants ~~who reported a feeling of~~reporting loneliness ~~had~~ were ~~a~~ significantly ~~higher~~ more likely to be frail ~~risk odds of non-disabled frailty compared to~~than ~~to those who did not feel so~~their counterparts (RRR= 1.45, 95% CI: 1.07-1.98). Sensitivity analysis ~~testing shows~~ suggested the results to be robust~~ness of results in relati on to the~~in quantifying these above associations (Supplementary ~~table~~ Table 1).

**Table 2:** Factors associated with non-disabled frailty and disability among the participants (N=1045)

| Characteristics | Disability ~~led~~ | | | ~~Non-disabled~~ F~~f~~railty | | |
|---|---|---|---|---|---|---|
| | RRR[1] | 95% CI | *P* | RRR | 95% CI | *P* |
| Age (years) | | | | | | |
| 60 – 69 | *Ref* | | | *Ref* | | |
| 70-79 | 2.56 | 1.72-3.80 | <0.001 | 1.08 | 0.72-1.62 | 0.718 |
| ≥ 80 | 10.00 | 4.64-21.56 | <0.001 | 3.23 | 1.41-7.37 | 0.005 |
| Formal schooling | | | | | | |
| No formal schooling | *Ref* | | | *Ref* | | |
| Having formal schooling | 0.75 | 0.53-1.06 | 0.109 | 0.84 | 0.62-1.14 | 0.270 |
| Family size | | | | | | |
| ≤4 | *Ref* | | | *Ref* | | |
| >4 | 1.25 | 0.87-1.80 | 0.235 | 1.39 | 1.01-1.92 | 0.047 |
| Residence | | | | | | |
| Urban | *Ref* | | | *Ref* | | |
| Rural | 0.61 | 0.39-0.96 | 0.032 | 0.69 | 0.46-1.01 | 0.059 |
| Problems with memory or concentration | | | | | | |
| No problem | *Ref* | | | *Ref* | | |
| Low memory or concentration | 2.73 | 1.91-3.91 | <0.001 | 1.56 | 1.12-2.17 | 0.002 |
| Feeling of loneliness | | | | | | |

| | | | | | | |
|---|---|---|---|---|---|---|
| No | *Ref* | | | *Ref* | | |
| Yes | 1.47 | 1.03-2.08 | 0.032 | 1.45 | 1.07-1.98 | 0.017 |
| Family members non-responsive to needs | | | | | | |
| No | *Ref* | | | *Ref* | | |
| Yes | 1.09 | 0.75-1.58 | 0.663 | 1.47 | 1.06-2.03 | 0.020 |

[1]RRR=Relative Risk Ratio; Note: The adjusted model contained all variables in Table 2.~~were considered together in this adjusted model~~

## Discussion

~~The current study~~With the aim~~ed~~ to examine ~~This study was the first to assess~~ the prevalence and determinants of non-disabled frailty among Bangladeshi older people, the current study found a 24.8%. ~~among the country's representative population. The estimated~~ prevalence of non-disabled frailty ~~in the current study was 24.8%. The multinomial regression analysis found that older~~and factors such as age, family size, memory or concentration problems, ~~adults aged ≥80 years, those having a family of ≥4 members, who had memory or concentration problems, had~~non-responsive family members ~~non-responsive to their needs and felt~~and loneliness associated with frailty. ~~lonely were more likely to report frailty.~~ Our study contributes to the ~~existing paucity of~~limited literature on frailty among ~~older adults residing in s~~South Asian older adults, specifically from Bangladesh~~countries~~.

Our reported 24.8% ~~The current study found 24.8% of~~ prevalence of frailty~~-~~ among older ~~people~~adults ~~, which~~ is similar to studies in India (26%) [11] and Italy (23.2%) [20]. A systematic

review~~s~~ ~~conducted~~ of studies on frailty among community-dwelling older adults ~~also~~ reported a pooled ~~frailty~~ prevalence of 10.7% [21]. In contrast, studies from two other South Asian countries, Nepal and Pakistan, have reported a higher prevalence of frailty [10, 12]. In a study from rural Nepal, 65% of participants were frail [10], while in the Pakistani study, 55.4% were frail [12]. The discrepancies in frailty prevalence between the current study and the existing literature may be attributable to ~~methodological~~ several factors, including different measurement tools, ~~sampling differences,~~ heterogeneity ~~of~~in participants' ages, ~~the living standard of people~~people's living standard, and the burden of NCDs. ~~, and the use of different scales.~~ ~~such as participants' age, poverty, the living standard of people, the burden of NCDs, and the use of different scales.~~

In our study, older people aged ≥ 80 were more likely to be ~~the odds of~~ frail than their younger counterparts~~ty were~~ ~~was~~ ~~higher~~ ~~among older people aged ≥ 80,~~ ~~in the higher age group,~~ which ~~is in line~~aligns with ~~several~~ existing ~~other studies~~literature [22, 23]. Several biological and physiological changes accompanying the ageing process~~ied by ageing, coupled with a high burden of NCDs among oldest adults,~~ and a high burden of NCDs among older adults explain the ~~findings of having higher frailty among these population group~~increased likelihood of frailty among older people [24]. Like many other countries of the world (e.g., India, China and Indonesia), Bangladesh is also facing an epidemiological transition, resulting in a higher burden of NCDs and ~~, including~~ multimorbidity, particularly among the olde~~r~~~~st~~ ~~adults~~ population [25], which might have contributed to a higher level of frailty ~~among them~~among the older ages. Additionally, frailty was found to be associated with cognitive impairment, such as having problems with memory and concentration, in our and previous studies [22, 23]~~Cognitive impairment, such as having problems~~

**Formatted:** Strikethrough

in memory and concentration, which may also add to the burden among older adults was found to be associated with frailty which is consistent with other studies [22, 23].

Consistent with the existing literature [26], Wwe found that older adults living in a large family and reportinged having their family members as non-responsive to their care had a higher chance likelihood of frailty. Several contextual factors together explain the observed findings between family and frailty. The social welfare systems for older people are non-existential in Bangladesh. Thus, at an older age, many Bangladeshi people financially, emotionally, and physically depend on adult children and receive care from them. The adult son, who is usually the sole provider for the family, has to meet the competing needs of all family members. In many Bangladeshi families that struggle to arrange the bread and butter, the demand for competing needs from many family members may result in sub-optimal care for the older family members [27]. Consequently, older adults may feel that the family members are non-responsive to their needs. The financial constraints could also be a barrier to good nutrition and access to health care for older parents The reasons for a higher chance of frailty among older people are that These two things are interlinked as families with many family members often face financial burdens. They struggle for and family members are more concentrated on arranging the bread and butter for the entire family, which resultings in a lack of care for the older family members, making them vulnerable to frailty. Moreover, On the other hand, having older people in the family also imposes puts an extra financial burden for their treatment and care, which necessitates putting extra additional effort to earn the required money for the family [28]. All these issues can contribute to compromising older people’s quality of life , which can resultmpromise older people’s quality of life and make them vulnerable

to ~~in limited care for the older population with an increased prevalence of~~ frailty~~, increasing their risk of falls, disability and premature death~~and other adverse health outcomes [29, 30].

Like other studies [19, 31], this study also found ~~that the prevalence of~~an association between frailty ~~is associated with~~and loneliness. Evidence suggests that high levels of loneliness result~~s~~ in declined mobility and increased difficulties with daily living activities [31]~~ and increased difficulties with daily living activities~~. Lonely people are more likely to be inactive [19], ~~and such inactivity~~which increases the risk of physical frailty [19, 31]. We acknowledge that in Bangladesh, where multigenerational households are common, ~~Due to cultural variations when compared to other countries,~~ living alone ~~among~~ is~~older persons in Bangladesh may~~ not ~~be~~ typical. ~~In Bangladesh, family and intergenerational care play a vital role for older adults.~~ Culturally, in Bangladesh, older people are attached to families, living with children and grandchildren [32]. Although living in a large multigenerational family offers social support, loneliness may be partly due to losing a spouse and friends, which is common in old age. Second, out-migration is very high in Bangladesh [33]. Even in a multigenerational family setting, a void is left by migrant children. Evidence suggests that the left-behind parents suffer the emotional cost of migration [34]. For some, loneliness may also be created due to neglect from family members which was more evident in our study as about 30% reported their family members were not responsive to their needs. As discussed in the previous paragraph, the financial constraint and demand to meet competing needs of multiple family members may result in sub-optimal care for the older family members and thus a feeling of neglect [27]. Recently, change in family dynamics is also noted in Bangladesh where nuclear family is being more popular ~~At an older age, After a certain period of life, many Bangladeshi people depending financially, emotionally, and physically depend on adult~~

children and receive ing care from them. In such a situation, they do not generally receive appropriate care and treatment from their family members due to older people's financial reliance on their children's wealth and compliance. there is culturally expected and appropriate. Although nowadays, it is seen thatIand in some cases,n some instances, many older adults are living with paid maids, beinglive with paid maids, separated from their children in Bangladesh [32]. However, this is contradictory with traditional family values and not well accepted in low and middle-income families [32]. According to previous literature, Older people''s limitedlimited social support from their children/ or family members and being separated from their children was related toincrease the likelihood of increasing depression, loneliness, and cognitive decline [35], all of which could can contribute to developing frailty.

**Implications for policy and practice**

Due to the demographic transition resulting in a burgeoning number of older adults in Bangladesh [2], the prevalence of frailty is projected to increase. Health and social care services are meagre meager for the older population in Bangladesh, and the existing family care is challenged. Many health-related issues that are preventable by treatment and medicine cannot be ensured due to limited availability of appropriate health care for older people. Moreover, traditional informal support that family members provide to older persons has lessened due to poverty, demographic changes, and globalization. The Government of Bangladesh has introduced National Policy on Ageing (2007), the Parents Care Act (2013), and Old Age Allowance (OAA) program [36], but on a limited scale. Yet, There is a lack of a national policy or strategy particularly focusing on old age health and access to social care services in Bangladesh which is critical to addressfocusing on old age health and social care issues in Bangladesh, which is critical to addressing frailty and

disability. Due to the absence of There is no healthcare insurance support available in Bangladesh,and resulting in high out-of-pocket costs for the family, which often they cannot affordpreventive services are underutilized [3]. Therefore, the introduction ofintroducing health insurance could be crucial to ensure access preventive services and ensure early diagnosis, treatment and delayed progression of frailty.care for long term illnesses like frailty. Bangladesh has recently adopted Mental Health Strategy (2020-2030) [37]. However, its implementation remained challenging. It is also important to incorporate psychosocial assistance to the older population in this strategy to ensure mental health for this vulnerable population group which is also linked with frailty. It would be particularly important to provide comprehensive supportive care for the older adults to address frailtyProviding comprehensive supportive care, including multidisciplinary care services, individualized care, and long-term follow-up, would be particularly important to address frailty. that may include multidisciplinary care services, individualized care, long-term follow-up and promoting physical activities. Strengthening primary care services, including promoting physical activities and ensuring proper nutrition and health education, are also proven effective in managingpromotion of physical activities, ensuring proper nutrition and health education also proven to be effective in management of frailty.

**Strengths and limitations of the study**

This study's study’s generalizability is one of its strengths, as it collected data from all eight divisions of Bangladesh. Other notable methodological strengths include the large sample size, usingand the use of a standard tool to measure outcomes, and data collection by trained and experienced surveyors. However, the cross-sectional study design limits the ability to establish a causal association. Due to the paucity of information literature regarding on frailty in Bangladesh,

the current study intended to quantify the burden and serve as a baseline. We recommend we intended to study the condition's prevalence and associated factors as an initial step. Using a longitudinal design in the future to better establish, the predictors for frailty in this population can be uncovered. Although we used a validated scale for measuring frailty, it was not explicitly validated among Bangladeshi older adults. Thus, future studies also have an opportunity to validate the FiND questionnaire in Bangladesh. Given the self-reported nature of the survey, we also anticipate measurement bias in our study its prevalence may be exaggerated due to the self-reported nature of the survey. Finally, our study is limited to quantitative analysis, which helped us to understand the potential association. However, to better understand the underlying reasons for these associations, specifically the observed relationships between family and frailty, we recommend a future qualitative study, as we did not explore the qualitative aspects of older people's experience of frailty in Bangladesh..

## Conclusion

Overall, the findings of the study suggest analyzing and understanding frailty and its associated components is an essential first step toward that preventive goal. The present study highlighted that around one in every four older adults had a non-disabled frailty in Bangladesh. Specifically, those relatively older, living in large families, suffering from cognitive problems or loneliness, and not properly cared for by their family members were more vulnerable to frailty. This was more prevalent among those who were aged, belonged to a large family, had cognitive problems or loneliness and who received less care from their family members. The findings of the study have significant implications for policy and practice. The findings suggest the need for providing comprehensivedeveloping interventions targeting the at-risk groups supportive careto address the

high prevalenceburden of frailty. Providing long-term care and relevant health promotion services may effectively prevent frailty and associated long-term adverse health outcomes. among Bangladeshi older adults for the older adults including that includes long term follow up and promotion ofe preventive services. Strengthening primary care services including promotion or physical activities and health education could also be of value in this regard.In line with the findings of the present research, emphasis should be given to those who are relatively aged, belong to a large family, suffering from cognitive problems or loneliness and who are not properly cared by their family members.

**Formatted:** Highlight


## Acknowledgements

We acknowledge the role of Sadia Sumaia Chowdhury, Programme Manager, ARCED Foundation and Md. Zahirul Islam, Project Associate, ARCED Foundation, for their support in data collection for the study.


## Contributors

**Sabuj Kanti Mistry**: Conceptualization, Methodology, Investigation, Software, Data curation, Formal analysis, Supervision, Writing- Original draft preparation. **ARM Mehrab Ali**: Conceptualization, Methodology, Investigation, Supervision, Writing- Original draft preparation. **Uday Narayan Yadav**: Conceptualization, Methodology, Writing- Original draft preparation. **Saruna Ghimire**: Writing- Original draft preparation. **Afsana Anwar**: Writing- Original draft preparation. **Md. Nazmul Huda**: Writing- Original draft preparation. **Fouzia Khanam**: Writing- Original draft preparation. **Rashidul Alam Mahumud**: Writing- Original draft preparation. **Ateeb**

**Ahmad Parray**: Writing- Original draft preparation. **Shovon Bhattacharjee**: Writing- Original draft preparation. **David Lim**: Writing- Reviewing and Editing. **Mark Fort Harris**: Writing- Reviewing and Editing.


## Funding

This research did not receive any external funding.


## Competing interest

The authors declare that they have no competing interest to disclose.

## Availability of data and materials

The datasets generated and/or analyzed during the current study are available as supporting file to this manuscript (Supplementary file 2). not publicly available due the organizational policy of the institution undertaking the research but are available from the corresponding author on reasonable request.

# References


1. World Health Organization. Ageing and health 2022 [18/12/2022]. Available from: https://www.who.int/news-room/fact-sheets/detail/ageing-and-health.
2. Kabir R, Khan HT, Kabir M, Rahman MTJESJ. Population ageing in Bangladesh and its implication on health care. 2013;9(33):34-47.
3. Mahumud RA, Sultana M, Kundu S, Rahman MA, Mistry SK, Kamara JK, et al. The burden of chronic diseases and patients' preference for healthcare services among adult patients suffering from chronic diseases in Bangladesh. Health Expectations. 2022.
4. Matthews NR, Porter GJ, Varghese M, Sapkota N, Khan MM, Lukose A, et al. Health and socioeconomic resource provision for older people in South Asian countries: Bangladesh, India, Nepal, Pakistan and Sri Lanka. 2020.
5. Yadav UN, Tamang MK, Thapa TB, Hosseinzadeh H, Harris MF, Yadav KK. Prevalence and determinants of frailty in the absence of disability among older population: a cross sectional study from rural communities in Nepal. BMC geriatrics. 2019;19(1):1-7.
6. Fried LP, Tangen CM, Walston J, Newman AB, Hirsch C, Gottdiener J, et al. Frailty in older adults: evidence for a phenotype. 2001;56(3):M146-M57.
7. Rogers NT, Marshall A, Roberts CH, Demakakos P, Steptoe A, Scholes SJPo. Physical activity and trajectories of frailty among older adults: Evidence from the English Longitudinal Study of Ageing. 2017;12(2):e0170878.
8. Khezrian M, Myint PK, McNeil C, Murray ADJG. A review of frailty syndrome and its physical, cognitive and emotional domains in the elderly. 2017;2(4):36.
9. Siriwardhana DD, Hardoon S, Rait G, Weerasinghe MC, Walters KR. Prevalence of frailty and prefrailty among community-dwelling older adults in low-income and middle-income countries: a systematic review and meta-analysis. BMJ open. 2018;8(3):e018195.
10. Yadav UN, Tamang MK, Thapa TB, Hosseinzadeh H, Harris MF, Yadav KKJBg. Prevalence and determinants of frailty in the absence of disability among older population: a cross sectional study from rural communities in Nepal. 2019;19(1):1-7.
11. Kashikar Y, Nagarkar AJIJoG. Prevalence and Determinants of Frailty in Older Adults in India. 2016;30(3).
12. Ali AA, Haq N, Chang K, Naqi S, Rafique M, Ismail M, et al. Impact of obesity on frailty in older population of Karachi, Pakistan. Advances in Medical, Dental and Health Sciences. 2019;2(4).
13. Siriwardhana DD, Weerasinghe MC, Rait G, Falcaro M, Scholes S, Walters KR. Prevalence of frailty in rural community-dwelling older adults in Kegalle district of Sri Lanka: a population-based cross-sectional study. BMJ open. 2019;9(1):e026314.
14. Rahman MM, Hamiduzzaman M, Akter M, Farhana Z, Hossain MK, Hasan MN, et al. Frailty indexed classification of Bangladeshi older adults' physio-psychosocial health and associated risk factors-a cross-sectional survey study. BMC geriatrics. 2021;21(1):1-10.
15. Mistry SK, Ali AM, Rahman MA, Yadav UN, Gupta B, Rahman MA, et al. Changes in tobacco use patterns during COVID-19 and their correlates among older adults in Bangladesh. International Journal of Environmental Research and Public Health. 2021;18(4):1779.
16. Mistry SK, Ali ARMM, Yadav UN, Huda MN, Ghimire S, Saha M, et al. Loneliness and its correlates among Bangladeshi older adults during the COVID-19 pandemic. Scientific Reports. 2022;12(1):15020.
17. BBS. Elderly Population in Bangladesh: Current features and future perspectives. Dhaka: Banglaedsh Bureau of Statistics, Ministry of Bangladesh, Government of Bangladesh, 2015.
18. Hughes ME, Waite LJ, Hawkley LC, Cacioppo JT. A short scale for measuring loneliness in large surveys: Results from two population-based studies. Research on aging. 2004;26(6):655-72.

19. Perissinotto CM, Cenzer IS, Covinsky KE. Loneliness in older persons: a predictor of functional decline and death. Archives of internal medicine. 2012;172(14):1078-84.
20. Battaggia A, Scalisi A, Novelletto BF, Fusello M, Michieli R, Cancian M. Prevalence of frailty in older people in Veneto (Italy). Journal of drug assessment. 2019;8(1):1-12.
21. Collard RM, Boter H, Schoevers RA, Oude Voshaar RC. Prevalence of frailty in community-dwelling older persons: a systematic review. Journal of the american geriatrics society. 2012;60(8):1487-92.
22. Curcio C-L, Henao G-M, Gomez F. Frailty among rural elderly adults. BMC geriatrics. 2014;14(1):1-9.
23. de Albuquerque Sousa ACP, Dias RC, Maciel ÁCC, Guerra RO. Frailty syndrome and associated factors in community-dwelling elderly in Northeast Brazil. Archives of gerontology and geriatrics. 2012;54(2):e95-e101.
24. Dziechciaz M, Filip R. Biological psychological and social determinants of old age: Bio-psycho-social aspects of human aging. Annals of Agricultural and Environmental Medicine. 2014;21(4).
25. Biswas T, Islam MS, Linton N, Rawal LB. Socio-economic inequality of chronic non-communicable diseases in Bangladesh. PloS one. 2016;11(11):e0167140.
26. Rahman MM, Hamiduzzaman M, Akter M, Farhana Z, Hossain MK, Hasan MN, et al. Frailty indexed classification of Bangladeshi older adults' physio-psychosocial health and associated risk factors-a cross-sectional survey study. BMC geriatrics. 2021;21(1):1-10.
27. Ali I, Azman A, Ahmed MK, Akhtar T, Deceukelier B. Assessment of the Needs of Rural Older People in Bangladesh. The International Journal of Community and Social Development. 2022;4(3):317-38.
28. Nortey ST, Aryeetey GC, Aikins M, Amendah D, Nonvignon J. Economic burden of family caregiving for elderly population in southern Ghana: the case of a peri-urban district. International journal for equity in health. 2017;16(1):1-9.
29. Kojima G, Liljas AEM, Iliffe S. Frailty syndrome: implications and challenges for health care policy. Risk management and healthcare policy. 2019:23-30.
30. Rosenberg T, Montgomery P, Hay V, Lattimer R. Using frailty and quality of life measures in clinical care of the elderly in Canada to predict death, nursing home transfer and hospitalisation-the frailty and ageing cohort study. BMJ open. 2019;9(11):e032712.
31. Perissinotto CM, Cenzer IS, Covinsky KEJAoim. Loneliness in older persons: a predictor of functional decline and death. 2012;172(14):1078-84.
32. Hossain MI, Akhtar T, Uddin MT. The elderly care services and their current situation in Bangladesh: An understanding from theoretical perspective. Journal of Medical Sciences. 2006;6(2):131-8.
33. Alam MZ, Mamun AA. Dynamics of internal migration in Bangladesh: Trends, patterns, determinants, and causes. Plos one. 2022;17(2):e0263878.
34. Kaur KJ, Sinha AK. Empty Nests: A Study on the Left-Behind Parents of Emigrated Children from Punjab (India). The Family Journal. 2023:10664807231157028.
35. Mehrabi F, Beland F. Frailty as a Moderator of the Relationship between Social Isolation and Health Outcomes in Community-Dwelling Older Adults. Int J Environ Res Public Health. 2021;18(4). Epub 2021/02/13. doi: 10.3390/ijerph18041675. PubMed PMID: 33572443; PubMed Central PMCID: PMCPMC7916171.
36. Begum A, Islam R. An Inclusive Approach to Care of the Elderly in Bangladesh. Dhaka: Bangladesh Institute of Development Studies, 2019.
37. Global Issues. Mental Health Strategic Plan for Bangladesh: An Overview 2022 [11/11/22]. Available from:

https://www.globalissues.org/news/2021/10/28/29200#:~:text=The%20National%20Mental%20Health%20Strategy,psychosocial%20wellbeing%20services%20and%20information.

## Figure title

**Figure 1:** Prevalence of non-disabled frailty by age and gender (n=1045)

## Supplementary materials

**Supplementary File 1:** Factors associated with non-disabled frailty and disability among the participants (N=1045)

**Supplementary File 2:** Dataset used for the analysis in the current study.

# Response to Reviews – "The burden of non-disabled frailty and its associated factors among older adults in Bangladesh"

Thank you for giving us the opportunity to revise and resubmit the manuscript and for the constructive reviewers' comments. In the following table, we have included the reviewers' comments and outlined our responses to each comment. We have systematically incorporated the reviewers' comments and suggestions, which have enhanced the manuscript. We are confident that the revisions have improved the quality of our manuscript.

**Editor comments**

| Comment/Suggestion | Authors' Responses |
|---|---|
| 1. Please ensure that your manuscript meets PLOS ONE's style requirements, including those for file naming. The PLOS ONE style templates can be found at<br><br>https://journals.plos.org/plosone/s/file?id=wjVg/PLOSOne_formatting_sample_main_body.pdf and<br><br>https://journals.plos.org/plosone/s/file?id=ba62/PLOSOne_formatting_sample_title_authors_affiliations.pdf. | We confirm that our manuscript meets PLOS ONE's style requirements. |
| 2. In your Data Availability statement, you have not specified where the minimal data set underlying the results described in your manuscript can be found. PLOS defines a study's minimal data set as the underlying data used to reach the conclusions drawn in the manuscript and any additional data required to replicate the reported study findings in their entirety. All PLOS journals require that the minimal data set be made fully available. For more information about our data policy, please see http://journals.plos.org/plosone/s/data-availability.<br><br>Upon re-submitting your revised manuscript, please upload your study's minimal underlying data set as either Supporting Information files or to a stable, public repository and include the relevant URLs, DOIs, or accession numbers within your revised cover letter. For a list of acceptable repositories, please see http://journals.plos.org/plosone/s/data-availability#loc-recommended-repositories. Any potentially identifying patient information must be fully anonymized. | The dataset used for the analysis of the current study is uploaded as a 'Supporting Information file.' Please refer to Supplementary file 2. |

| Comment/Suggestion | Authors' Responses |
|---|---|
| Important: If there are ethical or legal restrictions to sharing your data publicly, please explain these restrictions in detail. Please see our guidelines for more information on what we consider unacceptable restrictions to publicly sharing data: http://journals.plos.org/plosone/s/data-availability#loc-unacceptable-data-access-restrictions. Note that it is not acceptable for the authors to be the sole named individuals responsible for ensuring data access.<br><br>We will update your Data Availability statement to reflect the information you provide in your cover letter. | |
| 3. We note that you have indicated that data from this study are available upon request. PLOS only allows data to be available upon request if there are legal or ethical restrictions on sharing data publicly. For more information on unacceptable data access restrictions, please see http://journals.plos.org/plosone/s/data-availability#loc-unacceptable-data-access-restrictions.<br><br>In your revised cover letter, please address the following prompts:<br><br>a) If there are ethical or legal restrictions on sharing a de-identified data set, please explain them in detail (e.g., data contain potentially sensitive information, data are owned by a third-party organization, etc.) and who has imposed them (e.g., an ethics committee). Please also provide contact information for a data access committee, ethics committee, or other institutional body to which data requests may be sent.<br><br>b) If there are no restrictions, please upload the minimal anonymized data set necessary to replicate your study findings as either Supporting Information files or to a stable, public repository and provide us with the relevant URLs, DOIs, or accession numbers. For a list of acceptable repositories, please see http://journals.plos.org/plosone/s/data-availability#loc-recommended-repositories. | The dataset used for the analysis of the current study will be uploaded as a 'Supporting Information file.' Please refer to Supplementary file 2. |

| Comment/Suggestion | Authors' Responses |
|---|---|
| We will update your Data Availability statement on your behalf to reflect the information you provide. | |
| 4. Please review your reference list to ensure that it is complete and correct. If you have cited papers that have been retracted, please include the rationale for doing so in the manuscript text, or remove these references and replace them with relevant current references. Any changes to the reference list should be mentioned in the rebuttal letter that accompanies your revised manuscript. If you need to cite a retracted article, indicate the article's retracted status in the References list and also include a citation and full reference for the retraction notice. | We checked all references for correctness and completeness and edited them when necessary. |

**Reviewer 1**

| Comment/Suggestion | Authors' Responses |
|---|---|
| The authors estimated both non-disabled frailty and disability using the FiND questionnaire but I am not clear on the reasons for the inclusion of the questions about disability or the inclusion in the results of disability and robustness. | Thank you for your queries. The FiND questionnaire determines the prevalence of frailty, disability and robustness. Thus excluding a few components, i.e., disability, is not recommended. While this research aimed to determine the prevalence and factors associated with frailty only, we could not exclude disability as it is a part of the original scale. However, we only focused on interpreting the factors associated with frailty in the regression analysis, which has been clarified on page 17, lines 309-311 in the Result section and reads as below:<br><br>*"Although findings for both non-disabled frailty and disability are presented in Table 2, given our research question, we interpret here the findings relevant to non-disabled frailty only."* |
| The authors give some prominence to the male/female difference in non-disabled frailty yet the difference is small and does not reach statistical | Thanks for noting this. We have now removed the narratives relative to the male/female difference in non- |

| Comment/Suggestion | Authors' Responses |
|---|---|
| significance. I would recommend not giving this finding such prominence as it would be difficult to recommend policy based on this. | disabled frailty from the text. Please see page 3 lines 49-50 and page 17 lines 301-303. |
| Minor issues: Conclusions in the abstract; should read "present" rather than "preset". | Thank you for catching the typo. We corrected it. Please see page 4 line 61. |
| Figure 1; "Disable" should read "Disabled".<br>Figure 1, the Y=axis needs label (presumably %?) | Once again, thank you for catching the typo. We corrected the typo and labeled the Y-axis. |

**Reviewer 2**

| Comment/Suggestion | Authors' Responses |
|---|---|
| This research on non-disabled frailty among older adults is very important for health and well-being of elderly populations and for that I think this is an important study.<br>I have a few comments/questions that I think should be addressed properly. | Thank you for recognizing the importance of our study. We also appreciate your valuable comments and have addressed them in the manuscript. Please see our response below to each comment. |
| **Abstract** | |
| 1. Results: Revision of the results section required for further clarity. Please check for grammatical issues also. | Thank you for your comment. We revised the results section for further clarity, and the entire manuscript has been proofread for grammar issues and typos. Please refer to page 3-4, lines 48-57 for our revision to the Result section of the abstract. |
| 2. Conclusion: Your conclusion should be based on the findings of the study (Please revise). What do you mean by "comprehensive support care package"? Typographical errors (tense, spelling, etc). | Thank you for your comment. We revised the conclusion section of the Abstract, which now reads as below on page 4, lines 61-67:<br><br>*'The present study's findings suggest providing long-term care tailored towards frail older adults. Specifically, those relatively older, living in large families, suffering from cognitive problems or loneliness, and not properly cared for by their family members should be targeted for relevant health promotion interventions.'* |
| **Introduction** | |
| 3. Frailty suddenly appeared in the last sentence of the first paragraph and the link between first and second paragraph not properly done. Revision required. | Thank you for your comment. We have developed a case on frailty in paragraph 1 and established a smoother transition from paragraph 1 to paragraph 2. The first and second paragraphs of Introduction on page 5-6, lines 72-112 have been revised for clarity. |

| Comment/Suggestion | Authors' Responses |
|---|---|
| 4. Last sentence of the second paragraph was hard to grasp on what you were trying to convey. | Thank you for the comment. We substantially revised the second paragraph for clarity. We also noted a few unnecessary sentences in the paragraphs, which have been removed. Please see our revision in the second paragraph of Introduction on page 5-6, lines 91-112. |
| 5. Last paragraph requires attention and significant revision. Sentences beginning with "Thus" appeared in almost everywhere. | Thank you for the comment. The vocabulary and framing of sentences have been improved in the last paragraph. Please see our revision in the last paragraph of Introduction on page 7-8, lines 133-147. |
| 6. There are typographical errors (tense, spelling, grammar) that could be fixed to improve the readability of the manuscript (Introduction, Methodology, Results and Discussion). The introduction part requires significant rearrangement. Rationale should be done properly based on the objective. | Thank you for the comment. The entire manuscript has been proofread for typographical errors, and substantial revisions have been made. We have made corrections wherever applicable. The introduction section has been revamped with appropriate juxtapositions at appropriate positions (See page 5-8, lines 72-147). |
| 7. Referencing should be done properly. | Thank you for the comment. The Introduction section is now fully referenced wherever applicable. |
| **Methods and materials** | |
| 8. Sampling framework hard to make any senses. Please revise | Thank you for your suggestion. We have revised the sampling frame, which now reads as below under the "Study design and participants" sub-section of the method. Which is now read as bellow on page 8, lines 152-159:<br><br>*"Previously, between 2016–2020, the ARCED Foundation conducted ten different community-based studies and established a registry using demographic information from these studies, which served as the sampling frame for the current study. Notably, this sampling frame included households from all eight administrative divisions of Bangladesh."* |
| 9. Sample size based on the "response rate" and overall "response rate "creating confusion. Please revise accordingly. | Thank you for your suggestion. We have revised the sample size statement, which now reads as below under the "Study design and participants" sub-section of the Method on page 8-9, lines 159-163: |

| Comment/Suggestion | Authors' Responses |
|---|---|
| | *"Considering a 50% unknown prevalence with a 5% margin of error, 95% confidence level, and 90% power, a sample size of 1096 was calculated. However, only 1045 of 1096 approached participants responded to the study resulting in an overall response rate of approximately 95%."* |
| 10. "The inclusion criterion was the minimum age of 60 years" of whom? Please check the whole manuscript for sentence making so that conveying messages make senses. | Thank you for your suggestion. We have revised the sentence to indicate the minimum age of participants (See page 9, line 166).<br><br>We also proofread the entire manuscript for grammar and sentence structure. |
| 11. First sentence of the outcome measure is hard to grasp. Please revise. | Thank you for your suggestion. We have revised the sentence which reads as below on page 9, lines 172-173:<br><br>*'The outcome of interest was non-disabled frailty, measured using the 'Frail Non-Disabled (FiND) questionnaire' [18]'.* |
| 12. Multiple grammatical errors in this section. | Thank you for bringing that to our attention. We proofread the entire manuscript for grammar and sentence structure. |
| 13. What is "robust"? | 'Robust' is a category identified through the FiND questionnaire, which represents the absence of 'frailty' or 'disability.' We revisited the outcome variable definition to indicate the meaning of robust explicitly. Please refer to our revision on page 9-10, lines 172-189. |
| 14. Explanatory variables- what do you mean by "without partner" in the "marital status (married/without a partner)" variable? | Thank you for bringing that to our attention. We revised the statement to indicate that the latter category, i.e., without a partner included widowed, separated and never married. Please see our revision on page 10, lines 195-196 in the revised manuscript. |
| 15. How many research assistants you had recruited for the study? Did you provide any training before the actual data collection? If yes, how long? | Thank you for your suggestion. We have revised the manuscript to indicate the number and training of research assistants. Our revision includes the following sentences under the sub-heading "Data collection tools and techniques" of Methods on page 11, lines 224-228. |

| Comment/Suggestion | Authors' Responses |
|---|---|
| | *"Ten research assistants were recruited as surveyors based on their previous experiences administering health surveys on the electronic platform. The research assistants were trained extensively via Zoom meetings for three full days by the coauthors (SKM, AMA, UNY) before beginning the data collection."* |
| 16. Data collection tools and techniques require significant revision. | Thank you for your suggestion. We revised the 'Data collection tools and techniques' section for clarity. Please see our changes on page 11-12, lines 222-238. |
| 17. Statistical analyses lack proper guidance what you have done in the paper. | Thank you for your suggestion. We revised the 'Statistical analyses' section for clarity. Please see our changes on page 12-13, lines 241-258. |
| 18. Ethics approval: "All procedures performed in studies…." Did not understand? Second sentence failed to convey messages properly. Please revise. | Thank you for your suggestion. We revised the 'Ethics approval' section for clarity. Please see our changes on page 13, lines 261-266. |
| **Results** | |
| 19. Characteristics of the participants-Please revise with most important information that aligns with your objectives. | Thank you for your suggestion. We revised the 'Characteristics of the participants' section for clarity. Please see our changes on page 13-14, lines 274-291. |
| 20. 19.7% were considered disabled (more than 200 people out of 1045!). This is huge number. Can you please share what types of disability you found in this study? | The prevalence of disability was measured using the FiND questionnaire, which defines disability as an affirmative answer to at least one of the following questions. Thus, disability is defined in terms of mobility of the participants. For your convenience link to the questionnaire is also provided below.<br><br>A. Have you any difficulties at walking 400 meters?<br>B. Have you any difficulties at climbing up a flight of stairs?<br><br>https://frailty.net/wp-content/uploads/FiND_Questionnaire_English.pdf |

| Comment/Suggestion | Authors' Responses |
|---|---|
| 21. "perceiving family members to be non-responsive to their needs (RRR= 1.47, 95% CI: 1.06-2.03)…." what do you mean by "perceiving family members"? | Thank you for your suggestion. The sentence was revised and now reads as below on page 17-18, lines 316-317.<br><br>'………………..and those whose family members were non-responsive to their needs (RRR= 1.47, 95% CI: 1.06-2.03) had……………..' |
| 22. What is "concentration"? Provide the definitions of "low memory" and "concentration". | Thank you for your suggestion. We added a statement defining this variable in the methods section under "Explanatory variables" on page 11, lines 210-212. |
| 23. Please revise this statement- "participants who reported a feeling of loneliness had a significantly higher risk of frailty compared to those who did not feel so". | Thank you for your suggestion. The sentence was revised and now reads as below on page 18, lines 320-322.<br><br>*'Moreover, participants reporting loneliness were significantly more likely to be frail than their counterparts (RRR= 1.45, 95% CI: 1.07-1.98).'* |
| **Discussion** | |
| 24. Did not understand the first paragraph of the discussion section. Please revise to convey the messages properly. | Thank you for your suggestion. We revised the first paragraph of the 'Discussion' section for clarity. This now reads as below on page 20, lines 332-341:<br><br>*'With the aim to examine the prevalence and determinants of non-disabled frailty among Bangladeshi older people, the current study found a 24.8% prevalence of non-disabled frailty and factors such as age, family size, memory or concentration problems, non-responsive family members and loneliness associated with frailty. Our study contributes to the limited literature on frailty among South Asian older adults, specifically from Bangladesh.'* |
| 25. "The discrepancies in frailty prevalence between the study and the literature may be attributable to methodological factors such as…" and after that you provided examples of determinants not the methodological factors. This | Thank you for bringing that to our attention. The sentence was revised and now reads as below on page 21, lines 348-354.<br><br>*'The discrepancies in frailty prevalence between the current study and the existing literature may be attributable to several factors, including different measurement tools, heterogeneity in participants'* |

| Comment/Suggestion | Authors' Responses |
|---|---|
| paragraph requires significant revision. | *ages, people's living standard, and the burden of NCDs.'* |
| 26. "In our study, the odds of frailty were higher in the higher age group ...." In the results, you mentioned about RRR. Here you are talking about odds? Why, revise accordingly. | Thank you for pointing that out. We have revised the line, which now reads as below on page 21, lines 356-358:<br><br>*"In our study, older people aged ≥ 80 were more likely to be frail than their younger counterparts, which aligns with existing literature [22, 23]."* |
| 27. "Like many other countries of the world, Bangladesh is also facing an epidemiological transition" What are the countries you are talking about? Rich, poor, developing, developed? | Thank you for pointing that out. We have specified the countries and added corresponding citations. The revised sentence on page 21, lines 362-365 now reads as below:<br><br>*"Like many other countries of the world (e.g., India, China and Indonesia), Bangladesh is also facing an epidemiological transition, resulting in a higher burden of NCDs and multimorbidity, particularly among the older population [25], which might have contributed to a higher level of frailty among the older ages."* |
| 28. "We found that older adults living in a large family and reported having their family members non-responsive to their care had a higher chance of frailty. These two things are interlinked...." How they are interlinked? Did you analyze that? Did you mention that in the results? | Thank you for pointing that out. We restructured the discussion to establish the interlink between the two variables. Please refer to the fourth discussion paragraph, page 22-23, lines 371-391, for our revision. |
| 29. Explanation of the fourth paragraph of the discussion section is not comprehensive and logical enough. Please provide scientific explanations with relevant references. | Thank you for pointing that out. We restructured the fourth paragraph with evidence from the literature. Please refer to the fourth discussion paragraph, page 22-23, lines 371-391, for our revision. |
| 30. "After a certain period of life, depending financially, emotionally, and physically on children and receiving care from there is culturally expected and appropriate." Don't understand what you are trying to convey here? | Thank you for pointing that out. We have revised the paragraph substantially. Please refer to page 23-24, lines 393-422 for our revision. |

| Comment/Suggestion | Authors' Responses |
|---|---|
| 31. Need more elaborations on limitations of the study. | We have added statements to the existing limitations. Please refer to page 25-26, lines 454-470 for our revision. |
| **Conclusion** | |
| 32. "Overall, the findings of the study suggest analyzing and understanding frailty and its associated components is an essential first step toward that preventive goal" What are the associated components you are trying to imply here? | Thank you for your suggestion. We revised the conclusion section for clarity. Please refer to page 26-27, lines 473-489 for our revision. |
| 33. Please revise the conclusion section for better understanding. | Thank you for your suggestion. We revised the conclusion section for clarity. Please refer to to page 26-27, lines 473-489 for our revision. |
