# Supplementary material for: The burden of non-disabled frailty and its associated factors among older adults in Bangladesh
Source: PLoS One. 2023 Nov 28;18(11):e0294889. doi: 10.1371/journal.pone.0294889 (PMC10684086; doi:10.1371/journal.pone.0294889)
Supplement: Attachment — Submitted filename: PONE-D-23-00513_R2_MMR.pdf [file pone.0294889.s006.pdf]

PLOS ONE

The burden of non-disabled frailty and its associated factors among older adults in Bangladesh

--Manuscript Draft--

**Manuscript Number:**	PONE-D-23-00513R2
**Article Type:**	Research Article
**Full Title:**	The burden of non-disabled frailty and its associated factors among older adults in Bangladesh
**Short Title:**	Frailty among older adults in Bangladesh
**Corresponding Author:**	Sabuj Kanti Mistry<br>Daffodil International University<br>Dhaka, BANGLADESH
**Keywords:**	non-disabled frailty; older adults; Bangladesh
**Abstract:**	Objective<br>The present study aims to measure the prevalence of non-disabled frailty and its associated factors among Bangladeshi older adults.<br><br>Methods<br>This cross-sectional study was conducted in September 2021 among 1,045 Bangladeshi older adults (≥60 years). Telephone interviews, using a semi-structured questionnaire, collected data on participants' characteristics and levels of frailty. The non-disabled frailty was measured using the 'Frail Non-Disabled (FiND)' questionnaire. A multinomial logistic regression model assessed the factors associated with frailty among the participants.<br><br>Results<br>Around a quarter of the participants (24.8%) were frail. The multinomial regression analysis showed that older participants aged ≥80 years (RRR= 3.23, 95% CI: 1.41-7.37) were more likely to be frail compared to participants aged 60-69 years. Likewise, the participants living in a large family with ≥4 members (RRR= 1.39, 95% CI: 1.01-1.92) were more likely to be frail compared to those living in smaller families. Also, participants having memory or concentration problems (RRR= 1.56, 95% CI: 1.12-2.17) were more likely to be frail compared to those who were not suffering from these problems. Moreover, participants whose family members were non-responsive to their day-to-day assistance (RRR= 1.47, 95% CI: 1.06-2.03) were more likely to be frail compared to those whose family members were responsive. Furthermore, participants who were feeling lonely (RRR= 1.45, 95% CI: 1.07-1.98) were more likely to be frail than their counterparts who were not feeling lonely.<br><br>Conclusions<br>The findings of the present study suggest developing tailored interventions to address the burden of frailty among the older populations in Bangladesh. In particular, providing long-term care and health promotion activities can be of value in preventing frailty and reducing adverse health outcomes among this vulnerable population group.
**Order of Authors:**	Sabuj Kanti Mistry
	ARM Mehrab Ali
	Uday Narayan Yadav
	Saruna Ghimire
	Afsana Anwar
	Md. Nazmul Huda
	Fouzia Khanam
	Rashidul Alam Mahumud

Powered by Editorial Manager® and ProduXion Manager® from Aries Systems Corporation

	Ateeb Ahmad Parray
	Shovon Bhattacharjee
	David Lim
	Mark Fort Harris
**Response to Reviewers:**	Review response is added as an attachement.
**Additional Information:**	
**Question**	**Response**
**Financial Disclosure**<br><br>Enter a financial disclosure statement that describes the sources of funding for the work included in this submission. Review the submission guidelines for detailed requirements. View published research articles from *PLOS ONE* for specific examples.<br><br>This statement is required for submission and **will appear in the published article** if the submission is accepted. Please make sure it is accurate.<br><br>**Unfunded studies**<br>Enter: *The author(s) received no specific funding for this work.*<br><br>**Funded studies**<br>Enter a statement with the following details:<br>• Initials of the authors who received each award<br>• Grant numbers awarded to each author<br>• The full name of each funder<br>• URL of each funder website<br>• Did the sponsors or funders play any role in the study design, data collection and analysis, decision to publish, or preparation of the manuscript?<br>• **NO** - Include this sentence at the end of your statement: *The funders had no role in study design, data collection and analysis, decision to publish, or preparation of the manuscript.*<br>• **YES** - Specify the role(s) played.<br><br>* typeset	The author(s) received no specific funding for this work.
**Competing Interests**<br><br>Use the instructions below to enter a competing interest statement for this submission. On behalf of all authors,	The authors have declared that no competing interests exist.

disclose any competing interests that could be perceived to bias this work—acknowledging all financial support and any other relevant financial or non-financial competing interests.<br><br>This statement is required for submission and **will appear in the published article** if the submission is accepted. Please make sure it is accurate and that any funding sources listed in your Funding Information later in the submission form are also declared in your Financial Disclosure statement.<br><br>View published research articles from *PLOS ONE* for specific examples.<br><br>**NO authors have competing interests**<br><br>Enter: *The authors have declared that no competing interests exist.*<br><br>**Authors with competing interests**<br><br>Enter competing interest details beginning with this statement:<br><br>*I have read the journal's policy and the authors of this manuscript have the following competing interests: [insert competing interests here]*<br><br>* typeset	
**Ethics Statement**<br><br>Enter an ethics statement for this submission. This statement is required if the study involved:<br><br>• Human participants<br>• Human specimens or tissue<br>• Vertebrate animals or cephalopods<br>• Vertebrate embryos or tissues<br>• Field research<br><br>Write "N/A" if the submission does not require an ethics statement.	The study protocol was approved by the Institutional Review Board of the Institute of Health Economics, the University of Dhaka, Bangladesh (Ref: IHE/2020/1037).

General guidance is provided below. Consult the submission guidelines for detailed instructions. **Make sure that all information entered here is included in the Methods section of the manuscript.**<br><br>**Format for specific study types**<br><br>**Human Subject Research (involving human participants and/or tissue)**<br>• Give the name of the institutional review board or ethics committee that approved the study<br>• Include the approval number and/or a statement indicating approval of this research<br>• Indicate the form of consent obtained (written/oral) or the reason that consent was not obtained (e.g. the data were analyzed anonymously)<br><br>**Animal Research (involving vertebrate animals, embryos or tissues)**<br>• Provide the name of the Institutional Animal Care and Use Committee (IACUC) or other relevant ethics board that reviewed the study protocol, and indicate whether they approved this research or granted a formal waiver of ethical approval<br>• Include an approval number if one was obtained<br>• If the study involved *non-human primates*, add *additional details* about animal welfare and steps taken to ameliorate suffering<br>• If anesthesia, euthanasia, or any kind of animal sacrifice is part of the study, include briefly which substances and/or methods were applied<br><br>**Field Research**<br><br>Include the following details if this study involves the collection of plant, animal, or other materials from a natural setting:<br>• Field permit number<br>• Name of the institution or relevant body that granted permission	
**Data Availability**<br><br>Authors are required to make all data	Yes - all data are fully available without restriction

underlying the findings described fully available, without restriction, and from the time of publication. PLOS allows rare exceptions to address legal and ethical concerns. See the PLOS Data Policy and FAQ for detailed information.<br><br>A Data Availability Statement describing where the data can be found is required at submission. Your answers to this question constitute the Data Availability Statement and **will be published in the article**, if accepted.<br><br>**Important:** Stating 'data available on request from the author' is not sufficient. If your data are only available upon request, select 'No' for the first question and explain your exceptional situation in the text box.<br><br>Do the authors confirm that all data underlying the findings described in their manuscript are fully available without restriction?	
**Describe where the data may be found in full sentences. If you are copying our sample text, replace any instances of XXX with the appropriate details.**<br><br>• If the data are **held or will be held in a public repository**, include URLs, accession numbers or DOIs. If this information will only be available after acceptance, indicate this by ticking the box below. For example: *All XXX files are available from the XXX database (accession number(s) XXX, XXX.*).<br>• If the data are all contained **within the manuscript and/or Supporting Information files**, enter the following: *All relevant data are within the manuscript and its Supporting Information files.*<br>• If neither of these applies but you are able to provide **details of access elsewhere**, with or without limitations, please do so. For example:<br><br>*Data cannot be shared publicly because of [XXX]. Data are available from the*	All relevant data are within the manuscript and its Supporting Information files.

XXX Institutional Data Access / Ethics Committee (contact via XXX) for researchers who meet the criteria for access to confidential data.<br><br>*The data underlying the results presented in the study are available from (include the name of the third party and contact information or URL).*<br>• This text is appropriate if the data are owned by a third party and authors do not have permission to share the data.<br><br>* typeset	
Additional data availability information:	

Powered by Editorial Manager® and ProduXion Manager® from Aries Systems Corporation

Manuscript
Click here to access/download;Manuscript;Main document_R2_clean.docx

The burden of non-disabled frailty and its associated factors among older adults in Bangladesh

Sabuj Kanti Mistry[1,2,3], ARM Mehrab Ali[1], Uday Narayan Yadav[2,4], Saruna Ghimire[5], Afsana Anwar[6], Md. Nazmul Huda[1,7], Fouzia Khanam[8], Rashidul Alam Mahumud[9], Ateeb Ahmad Parray[10], Shovon Bhattacharjee[11], David Lim[12], Mark Fort Harris[2]

[1] ARCED Foundation, 13/1 Pallabi, Mirpur-12, Dhaka 1216, Bangladesh

[2] School of Population Health, University of New South Wales, Sydney, Australia

[3] Department of Public Health, Daffodil International University, Dhaka 1207, Bangladesh

[4] National Centre for Epidemiology and Population Health, The Australian National University, Canberra, ACT, Australia

[5] Department of Sociology and Gerontology and Scripps Gerontology Center, Miami University, Oxford, OH, USA

[6] Health and Nutrition, Social Assistance and Rehabilitation for the Physically Vulnerable (SARPV), SARPV Complex, Cox's Bazar 4700, Bangladesh

[7] Translational Health Research Institute, Western Sydney University, Campbeltown, NSW 2560, Australia

[8] Department of Public Health, North South University, Dhaka, Bangladesh

[9] NHMRC Clinical Trials Centre, Faculty of Medicine and Health, The University of Sydney, Camperdown, NSW 2006, Australia

[10] Department of International Health, Johns Hopkins Bloomberg School of Public Health, Johns Hopkins University, Baltimore, MD 21218, United States

[11] Biosecurity Program, The Kirby Institute, University of New South Wales, Sydney, Australia.

[12] University of Technology Sydney, Ultimo, NSW 2148, Australia

* Correspondence to: Sabuj Kanti Mistry, ARCED Foundation, 13/1 Pallabi, Mirpur-12, Dhaka, Bangladesh, Mobile: +8801716411922, E-mail: smitra411@gmail.com

Short title: Frailty among older adults in Bangladesh

Abstract

Objective

The present study aims to measure the prevalence of non-disabled frailty and its associated factors among Bangladeshi older adults.

Methods

This cross-sectional study was conducted in September 2021 among 1,045 Bangladeshi older adults (≥60 years). Telephone interviews, using a semi-structured questionnaire, collected data on participants' characteristics and levels of frailty. The non-disabled frailty was measured using the 'Frail Non-Disabled (FiND)' questionnaire. A multinomial logistic regression model assessed the factors associated with frailty among the participants.

Results

Around a quarter of the participants (24.8%) were frail. The multinomial regression analysis showed that older participants aged ≥80 years (RRR= 3.23, 95% CI: 1.41-7.37) were more likely to be frail compared to participants aged 60-69 years. Likewise, the participants living in a large family with ≥4 members (RRR= 1.39, 95% CI: 1.01-1.92) were more likely to be frail compared to those living in smaller families. Also, participants having memory or concentration problems (RRR= 1.56, 95% CI: 1.12-2.17) were more likely to be frail compared to those who were not suffering from these problems. Moreover, participants whose family members were non-responsive to their day-to-day assistance (RRR= 1.47, 95% CI: 1.06-2.03) were more likely to be frail compared to those whose family members were responsive. Furthermore, participants who

were feeling lonely (RRR= 1.45, 95% CI: 1.07-1.98) were more likely to be frail than their counterparts who were not feeling lonely.

Conclusions

The findings of the present study suggest developing tailored interventions to address the burden of frailty among the older populations in Bangladesh. In particular, providing long-term care and health promotion activities can be of value in preventing frailty and reducing adverse health outcomes among this vulnerable population group.

Keywords: non-disabled frailty; older adults; Bangladesh

Introduction

The world is rapidly transitioning into an ageing society. According to the World Health Organization (WHO), one in six individuals worldwide will be 60 years or older by 2030, and that proportion will quadruple by 2050 [1]. Low- and middle-income countries (LMICs) are projected to have two-thirds of their population aged 60 or older by 2050 [1]. In Bangladesh, reflecting this global demographic shift, the total number of older adults aged 60 years and above is expected to increase from 8 million to 44 million by 2050 [2]. This demographic change has resulted in an increased burden of conditions common in older age, including various non-communicable diseases (NCDs) and frailty [3]; the latter is now recognized as an emerging concern among older adults in South Asian countries, including Bangladesh [4].

According to Fried and colleagues [5], frailty is a condition where three out of five phenotypic criteria of an individual are met: shrinkage (weight loss), exhaustion, weaknesses, low gait speed, and low physical activity. Frailty predicts many long-term health outcomes, disability and mortality [6] and interferes with one's ability to live independently [7]. Frailty is common in older age due to biological consequences that result in the deterioration of physical, cognitive, and psychological functions [8]. This condition manifests as a state of increased vulnerability and decreased resilience, resulting in decreased physical activity, diminished cognitive performance, and compromised nutritional status [7].

There is evidence indicating a high burden of frailty in LMICs. A systematic review and meta-analysis of 56 studies conducted in LMICs documented that the prevalence of frailty and prefrailty among older adults aged 60 years and above was 17.4% and 49.3% respectively [9]. The

prevalence of frailty was high among older adults from South Asian countries, ranging between 25% and 65%. For instance, a Nepalese study reported that 65% of participants had frailty without disability [10]. In India, 26% of older adults were frail, while 63.6% were pre-frail, and only 10.4% were non-frail [11]. Studies conducted in Pakistan [12] and Sri Lanka [13] found that 55.4% and 15.2% of older adults were frail, with 44.6% and 48.5% being pre-frail, respectively. These studies also identified several determinants of frailty among older adults, such as age, education, occupation, out-of-pocket health care cost, depression, and lack of physical activities and care from family members [10, 11, 13].

Limited evidence exists on the prevalence and determinants of frailty among older adults in Bangladesh. The only study on frailty among Bangladeshi older adults showed that 61.6% of the participants had moderate to severe levels of frailty [14]. However, this study was conducted in a small geographical area of the country and used the 30-indicator Frailty Index scale that does not measure non-disabled frailty. Considering the fact that there is high burden of NCDs and limited access to health service facilities in Bangladesh, we anticipate a high prevalence of frailty among older adults. Therefore, the present study was undertaken to estimate the prevalence of of non-disabled frailty and its determinants among Bangladeshi older adults.

Materials and methods

Study design and participants

This cross-sectional study was conducted during September and October 2021. Previously, between 2016–2020, the ARCED (Aureolin Research, Consultancy, and Expertise Development) Foundation conducted ten different community-based studies in Bangladesh and established a

registry using demographic information from these studies, which served as the sampling frame for the current study. Notably, this sampling frame included households from all eight administrative divisions of Bangladesh. Considering a 50% prevalence with a 5% margin of error, 95% confidence level, and 90% power, a sample size of 1096 was calculated. However, only 1045 of 1096 approached participants responded to the study resulting in an overall response rate of approximately 95%. Based on the population distribution of older people by geography in Bangladesh, we adopted a probability proportionate to size (of the eight administrative divisions) approach to determine the size of older people in each division [15]. The inclusion criterion was the minimum age of 60 years of the participants. Exclusion criteria were suffering from any adverse mental conditions i.e., clinically diagnosed schizophrenia, bipolar mood disorder, dementia/cognitive impairment, and inability to communicate.

Measures

Outcome measure

The outcome of interest was non-disabled frailty, measured using the 'Frail Non-Disabled (FiND) questionnaire' [16]. The FiND questionnaire is designed to measure non-disabled frailty among the older population and was previously used among older adults in Nepal in a similar setting [6]. The FiND questionnaire contains five questions under two sections. The first two questions on mobility belong to the disability section, and the latter three on weight loss, exhaustion, and physical activity belong to the frailty section. Each five items are coded as 0 and 1, indicating negative and affirmative responses, respectively. Participants are categorized into one of three categories, i.e., "disabled," "frail," and "robust." If a participant responds affirmatively to at least one of the two disability items, they are classified as disabled. Frailty is when a participant

responds negatively to both disability questions but affirmatively to at least one frailty item. A participant is classified as "robust" when the sum of five items is zero, i.e., indicating the absence of both frailty or disability." A Cronbach α of 0.75 in the current study indicated the scale is reliable among our study population.

Explanatory variables

A literature review guided the selection of explanatory variables [10-13, 17]. Explanatory variables considered in this study were administrative divisions (Barishal, Chattogram, Dhaka, Mymensingh, Khulna, Rajshahi, Rangpur, Sylhet), age (categorized as 60-69, 70-79, and ≥80), gender (male/female), marital status (married and without a partner; latter included widowed, separated and never married), formal schooling (with and without formal schooling), family size (≤4 or >4), family monthly income in Bangladeshi Taka (BDT) (<5,000, 5,000-10,000, >10,000), residence (urban/rural), current occupation (employed, unemployed or retired), living arrangement (living alone or with family), walking distance to the nearest health center (<30 min/≥30 min), memory or concentration problems (no problem/low memory or concentration), suffering from at least one chronic conditions (yes/no), a perception that family members are non-responsive to their day-to-day assistance (yes/no), and feeling of loneliness (yes/no).

Self-reported information on the participant's ever diagnosis of chronic conditions (i.e., arthritis, hypertension, heart diseases, stroke, hypercholesterolemia, diabetes, chronic respiratory diseases, chronic kidney disease, and cancer) was collected as dichotomized yes/no responses. To explore if participants had any memory or concentration problems, we asked the following question: 'Do you have any memory (remembering things properly) or concentration (focusing/concentrating

properly while doing any action) problem?' Loneliness was measured using the 3-item UCLA Loneliness scale [18]. Each item on the scale is measured as a yes/no question. The three items included: how often do you feel: (i) lack companionship, (ii) left out, and (iii) isolated in the last two weeks? Each item in the scale is measured in terms of 3-item Likert responses: hardly ever (1 point), some of the time (2 points), and often (3 points). Participants were classified as lonely if they answered 'some of the time' or 'often' to any of the three items [18].

Data collection tools and techniques

Telephone interviews were conducted using a pre-tested semi-structured questionnaire, and data were collected electronically using SurveyCTO mobile app (https://www.surveycto.com/). Ten research assistants were recruited as surveyors based on their previous experiences administering health surveys on the electronic platform. The research assistants were trained extensively via Zoom meetings for three full days by the investigators (SKM, AMA, UNY) before beginning the data collection.

The English version of the questionnaire was first translated into Bengali and then back-translated to English by two researchers (SKM and AMA) to ensure the content's construct and face validity. The questionnaire was then piloted among a small sample (n=10) of older adults for cultural validation of contents and refinement of the questions. The piloted questionnaire did not receive any corrections/suggestions and thus was used as it is for the data collection. Each interview took around half an hour.

Statistical analyses

Descriptive analyses (i.e., frequency and percentage) were performed to explore the distribution of the sociodemographic characteristics of the respondents. To explore the factors independently associated with non-disabled frailty, multinomial logistic regression models were run, adjusting for important confounders. Covariates for adjustment were identified using the backward elimination method based on the Akaike information criterion (AIC). The initial model was run with all potential covariates listed in Table 1, and based on the AIC, age, formal schooling, family size, residence, problems with memory or concentration, loneliness, and non-responsive family members were retained. The final model was executed including these variables. The final model was also tested for sensitivity using the bootstrapping approach by resampling observations with 10,000 replications. The Relative Risk Ratio (RRR) and associated 95% confidence interval (95% CI) for the "disabled" and "frail" categories are provided with reference to the "robust" category in Table 2. All analyses were performed using the statistical software package Stata (Version 14.0).

Ethics approval

The study was approved by the Institutional Review Board of the Institute of Health Economics, the University of Dhaka, Bangladesh (Ref: IHE/2020/1037). Verbal informed consent was sought from the participants before administering the survey. Participation in the survey was voluntary and participants did not receive any compensation for their time.

Patient and public involvement

Patients and/or the public were not involved in developing research questions, designing and conducting the study, and disseminating the results.

Results

Characteristics of the participants

The sociodemographic characteristics of the participants are presented in Table 1. The majority of the participants were aged 60-69 years (75.6%), males (59.3%), and married (76.5%). More than half of the participants (51.7%) had no formal schooling, 66.8% belonged to a large family of more than four members, 82.6% lived in rural areas, and 61.1% were unemployed or retired during the survey period. Furthermore, 35.3% had problems with memory or concentration, 57.2% suffered from at least one non-communicable chronic condition, 29.4% had their family members non-responsive to their day-to-day assistance, and 45.7% felt lonely (Table 1).

Table 1: Characteristics of study participants (N=1045)

Characteristics	N	%
Administrative division		
Barishal	146	14.0
Chattogram	98	9.4
Dhaka	172	16.5
Mymensingh	69	6.6
Khulna	198	19.0
Rajshahi	145	13.9
Rangpur	161	15.4
Sylhet	56	5.4
Residence		
Urban	182	17.4

Rural	863	82.6
Age (in years)		
60 – 69	790	75.6
70-79	201	19.2
≥ 80	54	5.2
Gender		
Male	620	59.3
Female	425	40.7
Marital status		
Married	799	76.5
[1]Without partner	246	23.5
Formal schooling		
No formal schooling	540	51.7
Formally schooled	505	48.3
Family size		
≤4	347	33.2
>4	698	66.8
[2]Family monthly income (BDT)		
<5000	121	11.6
5000-10000	469	44.9
>10000	455	43.5
Current occupation		
Employed	407	39.0

Unemployed/retired	638	61.1
Living arrangement		
Living with family	992	94.9
Living alone	53	5.1
Walking distance to the nearest health centre		
<30 minute	581	55.6
≥30 minutes	464	44.4
Problems with memory or concentration		
No problem	676	64.7
Low memory or concentration	369	35.3
Suffering from at least one chronic condition		
No	447	42.8
Yes	598	57.2
Family members non-responsive to their day-to-day assistance		
No	738	70.6
Yes	307	29.4
Feeling of loneliness		
No	568	54.4
Yes	477	45.7

[1]Without partner group includes widowed, separated and never married. [2]One USD = 85.75 Bangladeshi Taka (BDT).

Prevalence of non-disabled frailty

Figure 1 shows the prevalence of non-disabled frailty, disability, and robustness by age and gender using the FiND questionnaire. We found that around one-fourth (24.8%) of the total participants (n=1045) had non-disabled frailty, while 19.7% had a disability. No statistically significant differences were observed in the prevalence of frailty and disability among participants in terms of age and sex.

Factors associated with non-disabled frailty

Table 2 shows the factors associated with non-disabled frailty and disability (with reference to the robust category) in the adjusted multinomial logistic regression model. Although findings for both non-disabled frailty and disability are presented in Table 2, given our research question, we interpret here the findings relevant to non-disabled frailty only. In the adjusted model, compared to the youngest age group (60-69 years), the oldest group (≥80 years) had more than three times higher risk of frailty (RRR= 3.23, 95% CI: 1.41-7.37). Likewise, participants living in large families with four or more members (RRR= 1.39, 95% CI: 1.01-1.92) had 39% higher risk of being frail compared to those living in small families. Also, participants whose family members were non-responsive to their day-to-day assistance (RRR= 1.47, 95% CI: 1.06-2.03) had 47% higher risk of being frail than those who had family members responsive to their day-to-day assistance. Participants with low memory and concentration problems had a 56% higher risk of being frail (RRR= 1.56, 95% CI: 1.12-2.17) than those without such problems. Moreover, participants reporting loneliness were significantly more likely to be frail than their counterparts (RRR= 1.45, 95% CI: 1.07-1.98). Sensitivity analysis suggested the results to be robust in quantifying consistent associations (Supplementary Table 1).

Table 2: Factors associated with non-disabled frailty and disability among the participants (N=1045)

Characteristics	Disability			Frailty		
	RRR[1]	95% CI	*P*	RRR	95% CI	*P*
Age (years)						
60 – 69	*Ref*			*Ref*		
70-79	2.56	1.72-3.80	<0.001	1.08	0.72-1.62	0.718
≥ 80	10.00	4.64-21.56	<0.001	3.23	1.41-7.37	0.005
Formal schooling						
No formal schooling	*Ref*			*Ref*		
Having formal schooling	0.75	0.53-1.06	0.109	0.84	0.62-1.14	0.270
Family size						
≤4	*Ref*			*Ref*		
>4	1.25	0.87-1.80	0.235	1.39	1.01-1.92	0.047
Residence						
Urban	*Ref*			*Ref*		
Rural	0.61	0.39-0.96	0.032	0.69	0.46-1.01	0.059
Problems with memory or concentration						
No problem	*Ref*			*Ref*		
Low memory or concentration	2.73	1.91-3.91	<0.001	1.56	1.12-2.17	0.002
Feeling of loneliness						
No	*Ref*			*Ref*		

Yes	1.47	1.03-2.08	0.032	1.45	1.07-1.98	0.017
Family members non-responsive to their day-to-day assistance						
No	*Ref*			*Ref*		
Yes	1.09	0.75-1.58	0.663	1.47	1.06-2.03	0.020

[1]RRR=Relative Risk Ratio; Note: The adjusted model contained all variables in Table 2.

Discussion

The present study revealed that 24.8% of the participants had non-disabled frailty and factors such as age, family size, memory or concentration problems, non-responsive family members and loneliness were associated with frailty. Our study complement to the limited literature on frailty among South Asian older adults, specifically from Bangladesh.

Our reported 24.8% prevalence of frailty among older adults is similar to studies in India (26%) [11] and Italy (23.2%) [19]. Likewise, a systematic review of studies conducted in developed countries on frailty among community-dwelling older adults reported a pooled prevalence of 10.7% [20]. In contrast, studies from two other South Asian countries, Nepal and Pakistan, have reported a higher prevalence of frailty compared to that of our study [10, 12]. In a study from rural Nepal, 65% of participants were frail [10], while in the Pakistani study, 55.4% were frail [12]. The prevalence of frailty varies across studies, which may be attributable to several factors, including different measurement tools, heterogeneity in participants' ages, people's living standard, and the burden of NCDs.

In our study, older people aged ≥ 80 were more likely to be frail than those aged 60-69 years, which aligns with existing literature [21, 22]. Several biological and physiological changes accompanying the ageing process and a high burden of NCDs among older adults explain their increased likelihood of frailty [23]. Like many other countries of the world (e.g., India, China and Indonesia), Bangladesh is also facing an epidemiological transition, resulting in a higher burden of NCDs [24], which might have contributed to a higher level of frailty among its older population. Additionally, frailty was found to be associated with cognitive impairment, such as having problems with memory and concentration, in the present study and in some prior studies [21, 22], which may also add to the burden among older adults.

Consistent with the existing literature [25], we found that older adults who were living in large families and reporting their family members as non-responsive to their day-to-day assistance had a higher likelihood of frailty. Several contextual factors together explain the observed findings between family and frailty. Social safety net interventions such as insurance and pensions are largely unavailable for older people in Bangladesh. Thus, at an older age, many Bangladeshi people financially, emotionally, and physically depend on their adult family members and receive care from them. Evidence suggests that Bangladeshi large families often suffer a lot in satisfying the daily needs of their family members and older family members also receive suboptimal care [26]. Consequently, older adults may feel that the family members are non-responsive to their needs. The financial constraints could also be a barrier to good nutrition and access to health care for older parents [27]. All these issues can compromise older people’s quality of life and make them vulnerable to frailty and other adverse health outcomes [28, 29].

Like other studies [18, 30], this study also found an association between frailty and loneliness. Evidence suggests that high levels of loneliness result in declined mobility and increased difficulties with daily living activities [30]. Lonely people are more likely to be inactive [18], which increases the risk of physical frailty [18, 30]. We acknowledge that in Bangladesh, where multigenerational households are common, living alone is not typical. Culturally, in Bangladesh, older people are attached to families, living with children and grandchildren [31]. Although living in a large multigenerational family offers social support, loneliness may be partly due to losing a spouse and friends, which is common in old age. Secondly, migration is very high in Bangladesh [32]. Even in a multigenerational family setting, a void is left by migrant children. Evidence suggests that the left-behind parents suffer the emotional cost of migration [33]. For some, loneliness may also be created due to neglect from family members which was more evident in our study as about 30% reported their family members were not responsive to their needs. As discussed in the previous paragraph, the financial constraint and demand to meet competing needs of multiple family members may result in sub-optimal care for the older family members and thus a feeling of neglect [26]. Recently, change in family dynamics is also noted in Bangladesh where nuclear family is being more popular and in some cases, older adults live with paid maids, separated from their children [31]. Older people’s limited social support from their children/family members and being separated from their children increase the likelihood of depression, loneliness, and cognitive decline [34], all of which can contribute to developing frailty.

Implications for policy and practice

Due to the demographic transition resulting in a burgeoning number of older adults in Bangladesh [2], the prevalence of frailty is projected to increase. Health and social care services are meager

for the older population in Bangladesh, and the existing family care is challenged. The Government of Bangladesh has introduced National Policy on Ageing (2007), the Parents Care Act (2013), and Old Age Allowance (OAA) program [35], but on a limited scale. There is a lack of a national policy or strategy focusing on old age health and social care issues in Bangladesh, which is critical to addressing frailty and disability. Due to the absence of healthcare insurance support and resulting high out-of-pocket costs, preventive services are underutilized [3]. Therefore, introducing health insurance could be crucial to access preventive services and ensure early diagnosis, treatment and delayed progression of frailty. Providing comprehensive supportive care, including multidisciplinary care services, individualized care, and long-term follow-up to address the holistic needs of older adults, would be particularly important to address frailty. Strengthening primary care services, including promoting physical activities and ensuring proper nutrition and health education, are also proven effective in managing frailty.

Strengths and limitations of the study

This study's generalizability is one of its strengths, as it collected data from all eight divisions of Bangladesh. Other notable methodological strengths include the large sample size, using a standard tool to measure outcomes, and data collection by trained and experienced surveyors. However, the cross-sectional design limits the ability to establish a causal association. Due to the paucity of literature on frailty in Bangladesh, the current study intended to quantify the burden and serve as a baseline for future studies. We recommend a longitudinal design in the future to better establish the predictors for frailty in this population. Although we used a validated scale for measuring frailty, it was not explicitly validated among Bangladeshi older adults. Thus, future studies also have an opportunity to validate the FiND questionnaire in Bangladesh. Given the self-

reported nature of the survey, we also anticipate measurement bias in our study. Finally, our study is limited to quantitative analysis, which helped us to understand the potential association. However, to better understand the underlying reasons for these associations, specifically the observed relationships between family and frailty, we recommend a future qualitative study.

Conclusion

The present study found one in four older adults had non-disabled frailty in Bangladesh. Specifically, those relatively older, living in large families, suffering from cognitive problems or loneliness, and not properly cared for by their family members were more vulnerable to frailty. The findings suggest the need for developing tailored interventions including early screening of frailty at primary health care services to address emerging burden of frailty. Moreover, providing long-term care and relevant health promotion services such as engagement in physical activities, providing nutritional support, and delivering older people friendly services may effectively prevent frailty and reduce associated long-term adverse health outcomes.

Acknowledgements

We acknowledge the role of Sadia Sumaia Chowdhury, Programme Manager, ARCED Foundation and Md. Zahirul Islam, Project Associate, ARCED Foundation, for their support in data collection for the study.

Contributors

Sabuj Kanti Mistry: Conceptualization, Methodology, Investigation, Software, Data curation, Formal analysis, Supervision, Writing- Original draft preparation. **ARM Mehrab Ali**:

Conceptualization, Methodology, Investigation, Supervision, Writing- Original draft preparation. **Uday Narayan Yadav**: Conceptualization, Methodology, Writing- Original draft preparation. **Saruna Ghimire**: Writing- Original draft preparation. **Afsana Anwar**: Writing- Original draft preparation. **Md. Nazmul Huda**: Writing- Original draft preparation. **Fouzia Khanam**: Writing- Original draft preparation. **Rashidul Alam Mahumud**: Writing- Original draft preparation. **Ateeb Ahmad Parray**: Writing- Original draft preparation. **Shovon Bhattacharjee**: Writing- Original draft preparation. **David Lim**: Writing- Reviewing and Editing. **Mark Fort Harris**: Writing- Reviewing and Editing.

Funding

This research did not receive any external funding.

Competing interest

The authors declare that they have no competing interest to disclose.

Availability of data and materials

The datasets generated and/or analyzed during the current study are available as supporting file to this manuscript (Supplementary file 2).

References

1. World Health Organization. Ageing and health 2022 [18/12/2022]. Available from: https://www.who.int/news-room/fact-sheets/detail/ageing-and-health.
2. Kabir R, Khan HT, Kabir M, Rahman MTJESJ. Population ageing in Bangladesh and its implication on health care. 2013;9(33):34-47.
3. Mahumud RA, Sultana M, Kundu S, Rahman MA, Mistry SK, Kamara JK, et al. The burden of chronic diseases and patients' preference for healthcare services among adult patients suffering from chronic diseases in Bangladesh. Health Expectations. 2022.
4. Matthews NR, Porter GJ, Varghese M, Sapkota N, Khan MM, Lukose A, et al. Health and socioeconomic resource provision for older people in South Asian countries: Bangladesh, India, Nepal, Pakistan and Sri Lanka. 2020.
5. Fried LP, Tangen CM, Walston J, Newman AB, Hirsch C, Gottdiener J, et al. Frailty in older adults: evidence for a phenotype. 2001;56(3):M146-M57.
6. Yadav UN, Tamang MK, Thapa TB, Hosseinzadeh H, Harris MF, Yadav KK. Prevalence and determinants of frailty in the absence of disability among older population: a cross sectional study from rural communities in Nepal. BMC geriatrics. 2019;19(1):1-7.
7. Rogers NT, Marshall A, Roberts CH, Demakakos P, Steptoe A, Scholes SJPo. Physical activity and trajectories of frailty among older adults: Evidence from the English Longitudinal Study of Ageing. 2017;12(2):e0170878.
8. Khezrian M, Myint PK, McNeil C, Murray ADJG. A review of frailty syndrome and its physical, cognitive and emotional domains in the elderly. 2017;2(4):36.
9. Siriwardhana DD, Hardoon S, Rait G, Weerasinghe MC, Walters KR. Prevalence of frailty and prefrailty among community-dwelling older adults in low-income and middle-income countries: a systematic review and meta-analysis. BMJ open. 2018;8(3):e018195.
10. Yadav UN, Tamang MK, Thapa TB, Hosseinzadeh H, Harris MF, Yadav KKJBg. Prevalence and determinants of frailty in the absence of disability among older population: a cross sectional study from rural communities in Nepal. 2019;19(1):1-7.
11. Kashikar Y, Nagarkar AJIJoG. Prevalence and Determinants of Frailty in Older Adults in India. 2016;30(3).
12. Ali AA, Haq N, Chang K, Naqi S, Rafique M, Ismail M, et al. Impact of obesity on frailty in older population of Karachi, Pakistan. Advances in Medical, Dental and Health Sciences. 2019;2(4).
13. Siriwardhana DD, Weerasinghe MC, Rait G, Falcaro M, Scholes S, Walters KR. Prevalence of frailty in rural community-dwelling older adults in Kegalle district of Sri Lanka: a population-based cross-sectional study. BMJ open. 2019;9(1):e026314.
14. Rahman MM, Hamiduzzaman M, Akter M, Farhana Z, Hossain MK, Hasan MN, et al. Frailty indexed classification of Bangladeshi older adults' physio-psychosocial health and associated risk factors-a cross-sectional survey study. BMC geriatrics. 2021;21(1):1-10.
15. BBS. Elderly Population in Bangladesh: Current features and future perspectives. Dhaka: Banglaedsh Bureau of Statistics, Ministry of Bangladesh, Government of Bangladesh, 2015.
16. Hughes ME, Waite LJ, Hawkley LC, Cacioppo JT. A short scale for measuring loneliness in large surveys: Results from two population-based studies. Research on aging. 2004;26(6):655-72.
17. Mistry SK, Ali AM, Rahman MA, Yadav UN, Gupta B, Rahman MA, et al. Changes in tobacco use patterns during COVID-19 and their correlates among older adults in Bangladesh. International Journal of Environmental Research and Public Health. 2021;18(4):1779.
18. Perissinotto CM, Cenzer IS, Covinsky KE. Loneliness in older persons: a predictor of functional decline and death. Archives of internal medicine. 2012;172(14):1078-84.

19. Battaggia A, Scalisi A, Novelletto BF, Fusello M, Michieli R, Cancian M. Prevalence of frailty in older people in Veneto (Italy). Journal of drug assessment. 2019;8(1):1-12.
20. Collard RM, Boter H, Schoevers RA, Oude Voshaar RC. Prevalence of frailty in community-dwelling older persons: a systematic review. Journal of the american geriatrics society. 2012;60(8):1487-92.
21. Curcio C-L, Henao G-M, Gomez F. Frailty among rural elderly adults. BMC geriatrics. 2014;14(1):1-9.
22. de Albuquerque Sousa ACP, Dias RC, Maciel ÁCC, Guerra RO. Frailty syndrome and associated factors in community-dwelling elderly in Northeast Brazil. Archives of gerontology and geriatrics. 2012;54(2):e95-e101.
23. Dziechciaz M, Filip R. Biological psychological and social determinants of old age: Bio-psycho-social aspects of human aging. Annals of Agricultural and Environmental Medicine. 2014;21(4).
24. Biswas T, Islam MS, Linton N, Rawal LB. Socio-economic inequality of chronic non-communicable diseases in Bangladesh. PloS one. 2016;11(11):e0167140.
25. Rahman MM, Hamiduzzaman M, Akter M, Farhana Z, Hossain MK, Hasan MN, et al. Frailty indexed classification of Bangladeshi older adults' physio-psychosocial health and associated risk factors-a cross-sectional survey study. BMC geriatrics. 2021;21(1):1-10.
26. Ali I, Azman A, Ahmed MK, Akhtar T, Deceukelier B. Assessment of the Needs of Rural Older People in Bangladesh. The International Journal of Community and Social Development. 2022;4(3):317-38.
27. Nortey ST, Aryeetey GC, Aikins M, Amendah D, Nonvignon J. Economic burden of family caregiving for elderly population in southern Ghana: the case of a peri-urban district. International journal for equity in health. 2017;16(1):1-9.
28. Kojima G, Liljas AEM, Iliffe S. Frailty syndrome: implications and challenges for health care policy. Risk management and healthcare policy. 2019:23-30.
29. Rosenberg T, Montgomery P, Hay V, Lattimer R. Using frailty and quality of life measures in clinical care of the elderly in Canada to predict death, nursing home transfer and hospitalisation-the frailty and ageing cohort study. BMJ open. 2019;9(11):e032712.
30. Perissinotto CM, Cenzer IS, Covinsky KEJAoim. Loneliness in older persons: a predictor of functional decline and death. 2012;172(14):1078-84.
31. Hossain MI, Akhtar T, Uddin MT. The elderly care services and their current situation in Bangladesh: An understanding from theoretical perspective. Journal of Medical Sciences. 2006;6(2):131-8.
32. Alam MZ, Mamun AA. Dynamics of internal migration in Bangladesh: Trends, patterns, determinants, and causes. Plos one. 2022;17(2):e0263878.
33. Kaur KJ, Sinha AK. Empty Nests: A Study on the Left-Behind Parents of Emigrated Children from Punjab (India). The Family Journal. 2023:10664807231157028.
34. Mehrabi F, Beland F. Frailty as a Moderator of the Relationship between Social Isolation and Health Outcomes in Community-Dwelling Older Adults. Int J Environ Res Public Health. 2021;18(4). Epub 2021/02/13. doi: 10.3390/ijerph18041675. PubMed PMID: 33572443; PubMed Central PMCID: PMCPMC7916171.
35. Begum A, Islam R. An Inclusive Approach to Care of the Elderly in Bangladesh. Dhaka: Bangladesh Institute of Development Studies, 2019.

Figure title

Figure 1: Prevalence of non-disabled frailty by age and gender (n=1045)

Supplementary materials

Supplementary File 1: Factors associated with non-disabled frailty and disability among the participants (N=1045)

Supplementary File 2: Dataset used for the analysis in the current study.

 Click here to access/download;Figure;Figure 1_R2.docx

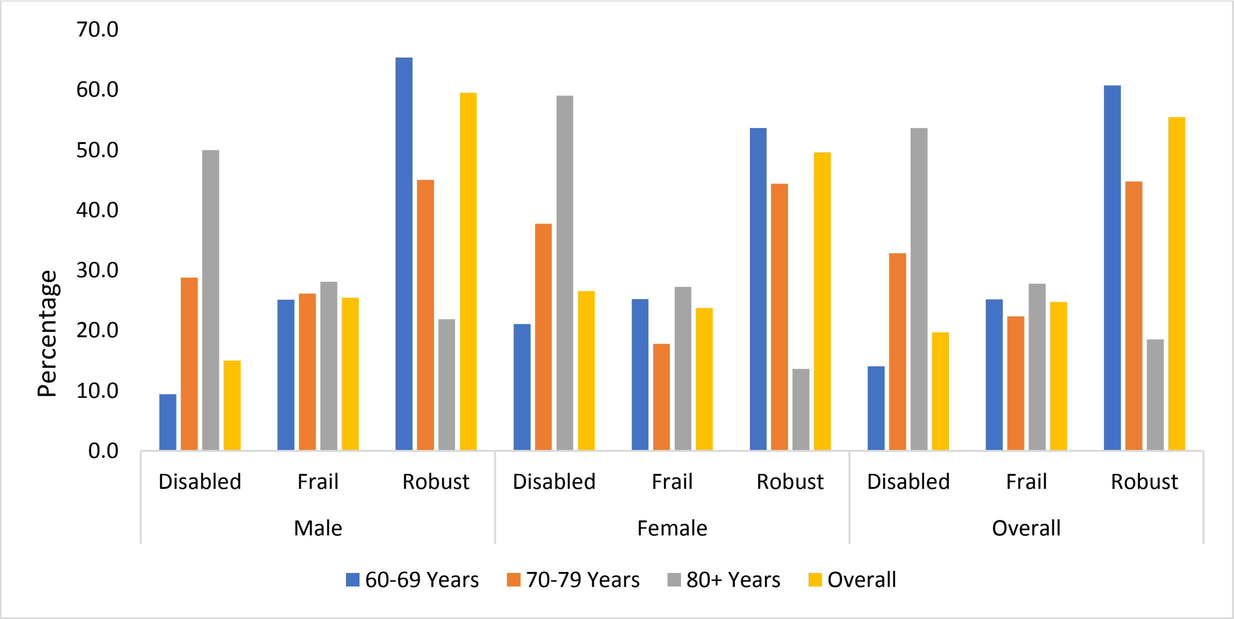

Figure 1: Prevalence of non-disabled frailty by age and gender (n=1045)

Click here to access/download
Supporting Information
Supplementary file 1_R2.docx

Click here to access/download
Supporting Information
Supplementary file 2_R2.dta

The burden of non-disabled frailty and its associated factors among older adults in Bangladesh

Sabuj Kanti Mistry[1,2,3], ARM Mehrab Ali[1], Uday Narayan Yadav[2,4], Saruna Ghimire[5], Afsana Anwar[6], Md. Nazmul Huda[1,7], Fouzia Khanam[8], Rashidul Alam Mahumud[9], Ateeb Ahmad Parray[10], Shovon Bhattacharjee[11], David Lim[~~7,~~12], Mark Fort Harris[2]

[1] ARCED Foundation, 13/1 Pallabi, Mirpur-12, Dhaka 1216, Bangladesh

[2] School of Population Health, University of New South Wales, Sydney, Australia

[3] Department of Public Health, Daffodil International University, Dhaka 1207, Bangladesh

[4] National Centre for Epidemiology and Population Health, The Australian National University, Canberra, ACT, Australia

[5] Department of Sociology and Gerontology and Scripps Gerontology Center, Miami University, Oxford, OH, USA

[6] Health and Nutrition, Social Assistance and Rehabilitation for the Physically Vulnerable (SARPV), SARPV Complex, Cox's Bazar 4700, Bangladesh

[7] Translational Health Research Institute, Western Sydney University, Campbeltown, NSW 2560, Australia

[8] Department of Public Health, North South University, Dhaka, Bangladesh

[9] NHMRC Clinical Trials Centre, Faculty of Medicine and Health, The University of Sydney, Camperdown, NSW 2006, Australia

[10] Department of International Health, Johns Hopkins Bloomberg School of Public Health, Johns Hopkins University, Baltimore, MD 21218, United States

[11] Biosecurity Program, The Kirby Institute, University of New South Wales, Sydney, Australia.

[12] SchoolUniversity of Technology Sydney, Ultimo, NSW 2148, of Health Sciences, Western Sydney University, Campbeltown, NSW 2560, Australia

* Correspondence to: Sabuj Kanti Mistry, ARCED Foundation, 13/1 Pallabi, Mirpur-12, Dhaka, Bangladesh, Mobile: +8801716411922, E-mail: smitra411@gmail.com

Short title: Frailty among older adults in Bangladesh

Abstract

Objective

The present study aims to measure the prevalence of non-disabled frailty and its associated factors among Bangladeshi older adults.

Methods

This cross-sectional study was conducted in September 2021 among 1,045 Bangladeshi older adults (≥60 years). Telephone interviews, using a semi-structured questionnaire, collected data on participants' characteristics and levels of frailty. The non-disabled frailty was measured using the 'Frail Non-Disabled (FiND)' questionnaire. A multinomial logistic regression model assessed the factors associated with frailty among the participants.

Results

Around a quarter of the participants (24.8%) were frail. The multinomial regression analysis showed that older participants aged ≥80 years (RRR= 3.23, 95% CI: 1.41-7.37) were more likely to be frail compared to participants aged 60-69 years.~~,~~ Likewise, the participants ~~those~~ living in a large family with ≥4 members (RRR= 1.39, 95% CI: 1.01-1.92) were more likely to be frail compared to those living in smaller families.~~,~~ Also, participants having memory or concentration problems (RRR= 1.56, 95% CI: 1.12-2.17) were more likely to be frail compared to those who were not suffering from these problems. Moreover, participants whose family members were non-responsive to their day-to-day ~~needs~~ assistance (RRR= 1.47, 95% CI: 1.06-2.03) were more likely to be frail compared to those whose family members were responsive. Furthermore, participants

who were~~and~~ feeling lonely (RRR= 1.45, 95% CI: 1.07-1.98) were more likely to be frail than their counterparts who were not feeling lonely.

Conclusions

The ~~present study's~~ findings of the present study suggest ~~providing long-term care~~developing tailored interventions to address the burden of frailty among the older populations in Bangladesh~~towards frail older adults~~. In particular, providing long-term care and health promotion activities can be of value in preventing frailty and reducing adverse health outcomes among this vulnerable population group. ~~Specifically, those relatively older, living in large families, suffering from cognitive problems or loneliness, and not properly cared for by their family members should be targeted for relevant health promotion interventions.~~

Keywords: non-disabled frailty; older adults; Bangladesh

Introduction

The world is rapidly transitioning into an ageing society. According to the World Health Organization (WHO), one in six individuals worldwide will be 60 years or older by 2030, and that proportion will quadruple by 2050 [1]. Low- and middle-income countries (LMICs) are projected to have two-thirds of their population aged 60 or older by 2050 [1]. In Bangladesh, reflecting this global demographic shift, the total number of older adults aged 60 years and above is expected to increase from 8 million to 44 million by 2050 [2]. This demographic change has resulted in an increased burden of conditions common in older age, including various non-communicable diseases (NCDs) and frailty [3]; the latter is now recognized as an emerging concern among older adults in South Asian countries, including Bangladesh [4].

~~Frailty is a condition characterized by low physical activity, cognitive performance, and nutritional status.~~According to Fried and colleagues [5], ~~provided the operational definition of frailty, which states that~~ frailty is a condition where three out of five phenotypic criteria of an individual are met: shrinkage (weight loss), exhaustion, weaknesses, low gait speed, and low physical activity. Frailty predicts many long-term health outcomes, disability and mortality [6] and interferes with one's ability to live independently [7]. Frailty is common in older age due to biological ~~senescence~~ consequences that result~~s~~ in the deterioration of physical, cognitive, and psychological functions [8]. This condition manifests as a state of increased vulnerability and decreased resilience, resulting in decreased physical activity, diminished cognitive performance, and compromised nutritional status [7].

There is evidence indicating a high burden of frailty in LMICs. A systematic review and meta-analysis ~~conducted with~~of 56 studies conducted in LMICs documented that the prevalence of frailty and prefrailty among ~~community-dwelling~~ older adults aged 60 years and above was 17.4% and 49.3%~~,~~ respectively ~~-~~[9]. The prevalence of frailty was ~~found to be higher~~high among older adults from South Asian countries, ranging between 25% and 65%. For instance, a Nepalese study reported that 65% of participants had frailty without disability [10]. In India, 26% of older adults were frail, while 63.6% were pre-frail, and only 10.4% were non-frail [11]. Studies conducted in Pakistan [12] and Sri Lanka [13] found that 55.4% and 15.2% of older adults were frail, with 44.6% and 48.5% being pre-frail, respectively. These studies ~~[10, 11, 13]~~ also identified several determinants of frailty among older adults, such as age, education, occupation, out-of-pocket health care cost, depression, and lack of physical activities and care from family members [10, 11, 13].

Limited evidence exists on the prevalence and determinants of frailty among older adults in Bangladesh. The only study on frailty among Bangladeshi older adults showed that 61.6% of the participants had moderate to severe levels of frailty [14]. However, this study was conducted in a small geographical area of the country and used the 30-indicator Frailty Index scale that does not measure non-disabled frailty. Considering the fact that there is high burden of NCDs and ~~coupled with~~ limited access to health~~care~~ ~~provision~~ service facilities in Bangladesh, we anticipate a high prevalence of frailty among older adults. Therefore, ~~further research is needed to investigate this topic in the Bangladeshi context. Accordingly,~~ the present study was undertaken ~~aims~~ to ~~quantify~~ estimate the ~~burden~~ prevalence of of non-disabled frailty and ~~identify~~ its determinants ~~associated factors~~ among Bangladeshi older adults.

Materials and methods

Study design and participants

This cross-sectional study was conducted ~~in~~ during September and October 2021. Previously, between 2016–2020, the ARCED (Aureolin Research, Consultancy, and Expertise Development) Foundation conducted ten different community-based studies in Bangladesh and established a registry using demographic information from these studies, which served as the sampling frame for the current study. Notably, this sampling frame included households from all eight administrative divisions of Bangladesh. Considering a 50% ~~unknown~~ prevalence with a 5% margin of error, 95% confidence level, and 90% power, a sample size of 1096 was calculated. However, only 1045 of 1096 approached participants responded to the study resulting in an overall response rate of approximately 95%. Based on the population distribution of older people by geography in Bangladesh, we adopted a probability proportionate to size (of the eight administrative divisions) approach to determine the size of older people in each division [15]. The inclusion criterion was the minimum age of 60 years of the participants. Exclusion criteria were suffering from any adverse mental conditions i.e., clinically diagnosed schizophrenia, bipolar mood disorder, dementia/cognitive impairment, and inability to communicate.

Measures

Outcome measure

The outcome of interest was non-disabled frailty, measured using the 'Frail Non-Disabled (FiND) questionnaire' [16]. The FiND questionnaire is designed to measure non-disabled frailty among the older population and was previously used among older adults in Nepal in a similar setting [6]. The FiND questionnaire contains five questions under two sections. The first two questions on

mobility ~~(i.e., items A and B)~~ belong to the disability section, and the latter three on weight loss, exhaustion, and physical activity ~~(i.e., items C, D and E)~~ belong to the frailty section. Each five items are coded as 0 and 1, indicating negative and affirmative responses, respectively. Participants are categorized into one of three categories, i.e., “disabled,” “frail,” and “robust.” If a participant responds affirmatively to at least one of the two disability items ~~(i.e., item A + B ≥ 1)~~, they are classified as disabled. Frailty is when a participant responds negatively to both disability questions but affirmatively to at least one frailty item ~~(i.e., A + B = 0 and C + D + E ≥ 1)~~. A participant is classified as “robust” when the sum of five items is zero, i.e., indicating the absence of both frailty or disability.” A Cronbach α of 0.75 in the current study indicated the scale is reliable among our study population.

Explanatory variables

A~~n~~ ~~extensive~~ literature review guided the selection of explanatory variables [10-13, 17]. Explanatory variables considered in this study were administrative divisions (Barishal, Chattogram, Dhaka, Mymensingh, Khulna, Rajshahi, Rangpur, Sylhet), age (categorized as 60-69, 70-79, and ≥80), ~~sex~~ gender (male/female), marital status (married and without a partner; latter included widowed, separated and never married), formal schooling (with and without formal schooling), family size (≤4 or >4), family monthly income in Bangladeshi Taka (BDT) (<5,000, 5,000-10,000, >10,000), residence (urban/rural), current occupation (employed, unemployed or retired), living arrangement (living alone or with family), walking distance to the nearest health center (<30 min/≥30 min), memory or concentration problems (no problem/low memory or concentration), suffering from at least one ~~non-communicable~~ chronic conditions (yes/no), a

perception that family members are non-responsive to their day-to-day needs assistance (yes/no), and feeling of loneliness (yes/no).

Self-reported information on the participant's ever diagnosis of nine non-communicable chronic conditions (i.e., arthritis, hypertension, heart diseases, stroke, hypercholesterolemia, diabetes, chronic respiratory diseases, chronic kidney disease, and cancer) was collected as dichotomized yes/no responses. To explore if participants had any memory or concentration problems, we asked the following question: 'Do you have any memory (remembering things properly) or concentration (focusing/concentrating properly while doing any action) problem?' Loneliness was measured using the 3-item UCLA Loneliness scale [18]. Each item on the scale is measured as a yes/no question. The three items included: how often do you feel: (i) lack companionship, (ii) left out, and (iii) isolated in the last two weeks? Each item in the scale is measured in terms of 3-item Likert responses: hardly ever (1 point), some of the time (2 points), and often (3 points). Participants were classified as lonely if they answered 'some of the time' or 'often' to any of the three items [18].

Data collection tools and techniques

Telephone interviews were conducted using a pre-tested semi-structured questionnaire, and data were collected electronically using SurveyCTO mobile app (https://www.surveycto.com/). Ten research assistants were recruited as surveyors based on their previous experiences administering health surveys on the electronic platform. The research assistants were trained extensively via Zoom meetings for three full days by the coauthors investigators (SKM, AMA, UNY) before beginning the data collection.

The English version of the questionnaire was first translated into Bengali and then back-translated to English by two researchers (SKM and AMA) to ensure the content's construct and face validity. The questionnaire was then piloted among a small sample (n=10) of older adults for cultural validation of contents and refinement of the questions. The piloted ~~response, excluded from data analyses,~~questionnaire did not ~~provide~~ receive any corrections/suggestions and thus was used as it is for the data collection. Each interview took around half an hour.

Statistical analyses

Descriptive analyses~~,~~ (i.e., frequency and percentage)~~,~~ were performed to explore the distribution of the sociodemographic characteristics of the respondents. To explore the factors independently associated with non-disabled frailty, multinomial logistic regression models were run, adjusting for important confounders. Covariates for adjustment were identified using the backward elimination method based on the Akaike information criterion (AIC). The initial model was run with all potential covariates listed in Table 1, and based on the AIC, ~~only~~ age, formal schooling, family size, residence, problems with memory or concentration, loneliness, and non-responsive family members were retained. The final model was executed including these variables~~/adjusted in the final model~~. The final model was also tested for sensitivity using the bootstrapping approach by resampling observations with 10,000 replications. The Relative Risk Ratio (RRR) and associated 95% confidence interval (95% CI) for the "disabled" and "frail" categories are provided with reference to the "robust" category in Table 2~~3~~. All analyses were performed using the statistical software package Stata (Version 14.0).

Ethics approval

The study ~~protocol~~ was approved by the Institutional Review Board of the Institute of Health Economics, the University of Dhaka, Bangladesh (Ref: IHE/2020/1037). Verbal informed consent was sought from the participants before administering the survey. Participation in the survey was voluntary and participants did not receive any compensation for their time.

Patient and public involvement

Patients and/or the public were not involved in developing research questions, designing and conducting the study, and disseminating the results.

Results

Characteristics of the participants

The sociodemographic characteristics of the participants are presented in Table 1. The majority of the participants were aged 60-69 years (75.6%), males (59.3%), and married (76.5%). More than half of the participants (51.7%) had no formal schooling, 66.8% belonged to a large family of more than four members, 82.6% lived in rural areas, and 61.1% were unemployed or retired during the survey period. Furthermore, 35.3% had problems with memory or concentration, 57.2% suffered from at least one non-communicable chronic condition, 29.4% had their family members non-responsive to their day-to-day ~~care~~assistance, and 45.7% felt lonely (Table 1).

Table 1: Characteristics of study participants (N=1045)

Characteristics	N	%
Administrative division		
Barishal	146	14.0

Chattogram	98	9.4
Dhaka	172	16.5
Mymensingh	69	6.6
Khulna	198	19.0
Rajshahi	145	13.9
Rangpur	161	15.4
Sylhet	56	5.4
Residence		
Urban	182	17.4
Rural	863	82.6
Age (in years)		
60 – 69	790	75.6
70-79	201	19.2
≥ 80	54	5.2
~~Sex~~Gender		
Male	620	59.3
Female	425	40.7
Marital status		
Married	799	76.5
[1]Without partner	246	23.5
Formal schooling		
No formal schooling	540	51.7
Formally schooled	505	48.3

Family size		
≤4	347	33.2
>4	698	66.8
[2]Family monthly income (BDT)		
<5000	121	11.6
5000-10000	469	44.9
>10000	455	43.5
Current occupation		
Employed	407	39.0
Unemployed/retired	638	61.1
Living arrangement		
Living with family	992	94.9
Living alone	53	5.1
Walking distance to the nearest health centre		
<30 minute	581	55.6
≥30 minutes	464	44.4
Problems with memory or concentration		
No problem	676	64.7
Low memory or concentration	369	35.3
Suffering from at least one ~~non-communicable~~ chronic condition		
No	447	42.8
Yes	598	57.2
Family members non-responsive to their day-to-day assistance		

No	738	70.6
Yes	307	29.4
Feeling of loneliness		
No	568	54.4
Yes	477	45.7

[1]Without partner group includes widowed, separated and never married. [2]One USD = 85.75 Bangladeshi Taka (BDT).

Prevalence of non-disabled frailty

Figure 1 shows the prevalence of non-disabled frailty, disability, and robustness by age and gender using the FiND questionnaire. We found that around one-fourth (24.8%) of the total participants (n=1045) had non-disabled frailty, while 19.7% ~~were categorized as disabled~~had a disability~~ and 55.5% as robust~~. No statistically significant differences were observed in the prevalence of frailty and disability among participants in terms of age and sex.

Factors associated with non-disabled frailty

Table 2 shows the factors associated with non-disabled frailty and disability (with reference to the robust category) in the adjusted multinomial logistic regression model. Although findings for both non-disabled frailty and disability are presented in Table 2, given our research question, we interpret here the findings relevant to non-disabled frailty only. In the adjusted model, compared to the youngest age group (60-69 years), the oldest group (≥80 years) had more than three times higher risk of frailty (RRR= 3.23, 95% CI: 1.41-7.37). Likewise, ~~those ~~participants living in ~~a~~ large~~r~~ families~~y~~ with four or more members (RRR= 1.39, 95% CI: 1.01-1.92) had 39% higher risk of being frail compared to those living in small families. Also, ~~and those ~~participants whose family

members were non-responsive to their day-to-day needs assistance (RRR= 1.47, 95% CI: 1.06-2.03) had 39% and 47% higher risk of being frail, respectively than those who had family members responsive to their day-to-day assistance. Participants with low memory and concentration problems had a 56% higher risk of being frail (RRR= 1.56, 95% CI: 1.12-2.17) than those without such problems. Moreover, participants reporting loneliness were significantly more likely to be frail than their counterparts (RRR= 1.45, 95% CI: 1.07-1.98). Sensitivity analysis suggested the results to be robust in quantifying these aboveconsistent associations (Supplementary Table 1).

Table 2: Factors associated with non-disabled frailty and disability among the participants (N=1045)

Characteristics	Disability			Frailty		
	RRR[1]	95% CI	*P*	RRR	95% CI	*P*
Age (years)						
60 – 69	*Ref*			*Ref*		
70-79	2.56	1.72-3.80	<0.001	1.08	0.72-1.62	0.718
≥ 80	10.00	4.64-21.56	<0.001	3.23	1.41-7.37	0.005
Formal schooling						
No formal schooling	*Ref*			*Ref*		
Having formal schooling	0.75	0.53-1.06	0.109	0.84	0.62-1.14	0.270
Family size						
≤4	*Ref*			*Ref*		
>4	1.25	0.87-1.80	0.235	1.39	1.01-1.92	0.047
Residence						
Urban	*Ref*			*Ref*		
Rural	0.61	0.39-0.96	0.032	0.69	0.46-1.01	0.059
Problems with memory or concentration						
No problem	*Ref*			*Ref*		
Low memory or concentration	2.73	1.91-3.91	<0.001	1.56	1.12-2.17	0.002
Feeling of loneliness						
No	*Ref*			*Ref*		

Yes	1.47	1.03-2.08	0.032	1.45	1.07-1.98	0.017
Family members non-responsive to their day-to-day ~~needs~~assistance						
No	*Ref*			*Ref*		
Yes	1.09	0.75-1.58	0.663	1.47	1.06-2.03	0.020

[1]RRR=Relative Risk Ratio; Note: The adjusted model contained all variables in Table 2.

Discussion

~~With the aim to examine the prevalence and determinants of non-disabled frailty among Bangladeshi older people~~The present study revealed that~~,~~ ~~the current study found a 24.8% prevalence of~~ 24.8% of the participants had non-disabled frailty and factors such as age, family size, memory or concentration problems, non-responsive family members and loneliness were associated with frailty. Our study ~~contributes~~ complement to the limited literature on frailty among South Asian older adults, specifically from Bangladesh.

Our reported 24.8% prevalence of frailty among older adults is similar to studies in India (26%) [11] and Italy (23.2%) [19]. Likewise, ~~A~~a systematic review of studies conducted in developed countries on frailty among community-dwelling older adults reported a pooled prevalence of 10.7% [20]. In contrast, studies from two other South Asian countries, Nepal and Pakistan, have reported a higher prevalence of frailty compared to that of our study [10, 12]. In a study from rural Nepal, 65% of participants were frail [10], while in the Pakistani study, 55.4% were frail [12]. The ~~discrepancies in~~prevalence of frailty ~~prevalence between the current study and the existing literature~~varies across studies, which may be attributable to several factors, including different

measurement tools, heterogeneity in participants' ages, people's living standard, and the burden of NCDs.

In our study, older people aged ≥ 80 were more likely to be frail than ~~their younger counterparts~~those aged 60-69 years, which aligns with existing literature [21, 22]. Several biological and physiological changes accompanying the ageing process and a high burden of NCDs among older adults explain their increased likelihood of frailty ~~among older people~~ [23]. Like many other countries of the world (e.g., India, China and Indonesia), Bangladesh is also facing an epidemiological transition, resulting in a higher burden of NCDs~~ and multimorbidity, particularly among the older population~~ [24], which might have contributed to a higher level of frailty among ~~the~~ its older ~~ages~~population. Additionally, frailty was found to be associated with cognitive impairment, such as having problems with memory and concentration, in ~~our~~ the present study and in some ~~previous~~ prior studies [21, 22], which may also add to the burden among older adults.

Consistent with the existing literature [25], we found that older adults who were living in ~~a~~ large famil~~y~~ies and reporting their family members as non-responsive to their day-to-day ~~care~~ assistance had a higher likelihood of frailty. Several contextual factors together explain the observed findings between family and frailty. ~~The s~~Social ~~welfare~~ safety net interventions such as insurance and pensions are largely unavailable ~~systems~~ for older people ~~are non-existential~~ in Bangladesh. Thus, at an older age, many Bangladeshi people financially, emotionally, and physically depend on their adult ~~children~~ family members and receive care from them. ~~The adult son, who is usually the sole provider for the family, has to meet the competing needs of all family members.~~ Evidence suggests that ~~In many~~ Bangladeshi large families -often suffer a lot in satisfying the daily needs of their

family members and older family members also receive suboptimal care ~~that struggle to arrange the bread and butter, the demand for competing needs from many family members may result in sub-optimal care for the older family members~~ [26]. Consequently, older adults may feel that the family members are non-responsive to their needs. The financial constraints could also be a barrier to good nutrition and access to health care for older parents [27]. All these issues can compromise older people's quality of life and make them vulnerable to frailty and other adverse health outcomes [28, 29].

Like other studies [18, 30], this study also found an association between frailty and loneliness. Evidence suggests that high levels of loneliness result in declined mobility and increased difficulties with daily living activities [30]. Lonely people are more likely to be inactive [18], which increases the risk of physical frailty [18, 30]. We acknowledge that in Bangladesh, where multigenerational households are common, living alone is not typical. Culturally, in Bangladesh, older people are attached to families, living with children and grandchildren [31]. Although living in a large multigenerational family offers social support, loneliness may be partly due to losing a spouse and friends, which is common in old age. Second, ~~out-~~migration is very high in Bangladesh [32]. Even in a multigenerational family setting, a void is left by migrant children. Evidence suggests that the left-behind parents suffer the emotional cost of migration [33]. For some, loneliness may also be created due to neglect from family members which was more evident in our study as about 30% reported their family members were not responsive to their needs. As discussed in the previous paragraph, the financial constraint and demand to meet competing needs of multiple family members may result in sub-optimal care for the older family members and thus a feeling of neglect [26]. Recently, change in family dynamics is also noted in Bangladesh where

nuclear family is being more popular and in some cases, older adults live with paid maids, separated from their children [31]. Older people's limited social support from their children/family members and being separated from their children increase the likelihood of depression, loneliness, and cognitive decline [34], all of which can contribute to developing frailty.

Implications for policy and practice

Due to the demographic transition resulting in a burgeoning number of older adults in Bangladesh [2], the prevalence of frailty is projected to increase. Health and social care services are meager for the older population in Bangladesh, and the existing family care is challenged. The Government of Bangladesh has introduced National Policy on Ageing (2007), the Parents Care Act (2013), and Old Age Allowance (OAA) program [35], but on a limited scale. There is a lack of a national policy or strategy focusing on old age health and social care issues in Bangladesh, which is critical to addressing frailty and disability. Due to the absence of healthcare insurance support and resulting high out-of-pocket costs, preventive services are underutilized [3]. Therefore, introducing health insurance could be crucial to access preventive services and ensure early diagnosis, treatment and delayed progression of frailty. Providing comprehensive supportive care, including multidisciplinary care services, individualized care, and long-term follow-up to address the holistic needs of older adults, would be particularly important to address frailty. Strengthening primary care services, including promoting physical activities and ensuring proper nutrition and health education, are also proven effective in managing frailty.

Strengths and limitations of the study

This study's generalizability is one of its strengths, as it collected data from all eight divisions of Bangladesh. Other notable methodological strengths include the large sample size, using a standard tool to measure outcomes, and data collection by trained and experienced surveyors. However, the cross-sectional design limits the ability to establish a causal association. Due to the paucity of literature on frailty in Bangladesh, the current study intended to quantify the burden and serve as a baseline for future studies. We recommend a longitudinal design in the future to better establish the predictors for frailty in this population. Although we used a validated scale for measuring frailty, it was not explicitly validated among Bangladeshi older adults. Thus, future studies also have an opportunity to validate the FiND questionnaire in Bangladesh. Given the self-reported nature of the survey, we also anticipate measurement bias in our study. Finally, our study is limited to quantitative analysis, which helped us to understand the potential association. However, to better understand the underlying reasons for these associations, specifically the observed relationships between family and frailty, we recommend a future qualitative study.

Conclusion

The present study ~~highlighted that around~~found one in four older adults had non-disabled frailty in Bangladesh. Specifically, those relatively older, living in large families, suffering from cognitive problems or loneliness, and not properly cared for by their family members were more vulnerable to frailty. ~~The findings of the study have significant implications for policy and practice.~~ The findings suggest the need for developing tailored interventions including early screening of frailty at primary health care services ~~targeting the at-risk groups~~ to address ~~the~~ emerging ~~high~~ burden of frailty. Moreover, ~~P~~providing long-term care and relevant health promotion services such as engagement in physical activities, providing nutritional support, and delivering older

people friendly services may effectively prevent frailty and reduce associated long-term adverse health outcomes.

Acknowledgements

We acknowledge the role of Sadia Sumaia Chowdhury, Programme Manager, ARCED Foundation and Md. Zahirul Islam, Project Associate, ARCED Foundation, for their support in data collection for the study.

Contributors

Sabuj Kanti Mistry: Conceptualization, Methodology, Investigation, Software, Data curation, Formal analysis, Supervision, Writing- Original draft preparation. **ARM Mehrab Ali**: Conceptualization, Methodology, Investigation, Supervision, Writing- Original draft preparation. **Uday Narayan Yadav**: Conceptualization, Methodology, Writing- Original draft preparation. **Saruna Ghimire**: Writing- Original draft preparation. **Afsana Anwar**: Writing- Original draft preparation. **Md. Nazmul Huda**: Writing- Original draft preparation. **Fouzia Khanam**: Writing- Original draft preparation. **Rashidul Alam Mahumud**: Writing- Original draft preparation. **Ateeb Ahmad Parray**: Writing- Original draft preparation. **Shovon Bhattacharjee**: Writing- Original draft preparation. **David Lim**: Writing- Reviewing and Editing. **Mark Fort Harris**: Writing- Reviewing and Editing.

Funding

This research did not receive any external funding.

Competing interest

The authors declare that they have no competing interest to disclose.

Availability of data and materials

The datasets generated and/or analyzed during the current study are available as supporting file to this manuscript (Supplementary file 2).

References

1. World Health Organization. Ageing and health 2022 [18/12/2022]. Available from: https://www.who.int/news-room/fact-sheets/detail/ageing-and-health.
2. Kabir R, Khan HT, Kabir M, Rahman MTJESJ. Population ageing in Bangladesh and its implication on health care. 2013;9(33):34-47.
3. Mahumud RA, Sultana M, Kundu S, Rahman MA, Mistry SK, Kamara JK, et al. The burden of chronic diseases and patients' preference for healthcare services among adult patients suffering from chronic diseases in Bangladesh. Health Expectations. 2022.
4. Matthews NR, Porter GJ, Varghese M, Sapkota N, Khan MM, Lukose A, et al. Health and socioeconomic resource provision for older people in South Asian countries: Bangladesh, India, Nepal, Pakistan and Sri Lanka. 2020.
5. Fried LP, Tangen CM, Walston J, Newman AB, Hirsch C, Gottdiener J, et al. Frailty in older adults: evidence for a phenotype. 2001;56(3):M146-M57.
6. Yadav UN, Tamang MK, Thapa TB, Hosseinzadeh H, Harris MF, Yadav KK. Prevalence and determinants of frailty in the absence of disability among older population: a cross sectional study from rural communities in Nepal. BMC geriatrics. 2019;19(1):1-7.
7. Rogers NT, Marshall A, Roberts CH, Demakakos P, Steptoe A, Scholes SJPo. Physical activity and trajectories of frailty among older adults: Evidence from the English Longitudinal Study of Ageing. 2017;12(2):e0170878.
8. Khezrian M, Myint PK, McNeil C, Murray ADJG. A review of frailty syndrome and its physical, cognitive and emotional domains in the elderly. 2017;2(4):36.
9. Siriwardhana DD, Hardoon S, Rait G, Weerasinghe MC, Walters KR. Prevalence of frailty and prefrailty among community-dwelling older adults in low-income and middle-income countries: a systematic review and meta-analysis. BMJ open. 2018;8(3):e018195.
10. Yadav UN, Tamang MK, Thapa TB, Hosseinzadeh H, Harris MF, Yadav KKJBg. Prevalence and determinants of frailty in the absence of disability among older population: a cross sectional study from rural communities in Nepal. 2019;19(1):1-7.
11. Kashikar Y, Nagarkar AJIJoG. Prevalence and Determinants of Frailty in Older Adults in India. 2016;30(3).
12. Ali AA, Haq N, Chang K, Naqi S, Rafique M, Ismail M, et al. Impact of obesity on frailty in older population of Karachi, Pakistan. Advances in Medical, Dental and Health Sciences. 2019;2(4).
13. Siriwardhana DD, Weerasinghe MC, Rait G, Falcaro M, Scholes S, Walters KR. Prevalence of frailty in rural community-dwelling older adults in Kegalle district of Sri Lanka: a population-based cross-sectional study. BMJ open. 2019;9(1):e026314.
14. Rahman MM, Hamiduzzaman M, Akter M, Farhana Z, Hossain MK, Hasan MN, et al. Frailty indexed classification of Bangladeshi older adults' physio-psychosocial health and associated risk factors-a cross-sectional survey study. BMC geriatrics. 2021;21(1):1-10.
15. BBS. Elderly Population in Bangladesh: Current features and future perspectives. Dhaka: Banglaedsh Bureau of Statistics, Ministry of Bangladesh, Government of Bangladesh, 2015.
16. Hughes ME, Waite LJ, Hawkley LC, Cacioppo JT. A short scale for measuring loneliness in large surveys: Results from two population-based studies. Research on aging. 2004;26(6):655-72.
17. Mistry SK, Ali AM, Rahman MA, Yadav UN, Gupta B, Rahman MA, et al. Changes in tobacco use patterns during COVID-19 and their correlates among older adults in Bangladesh. International Journal of Environmental Research and Public Health. 2021;18(4):1779.
18. Perissinotto CM, Cenzer IS, Covinsky KE. Loneliness in older persons: a predictor of functional decline and death. Archives of internal medicine. 2012;172(14):1078-84.

19. Battaggia A, Scalisi A, Novelletto BF, Fusello M, Michieli R, Cancian M. Prevalence of frailty in older people in Veneto (Italy). Journal of drug assessment. 2019;8(1):1-12.
20. Collard RM, Boter H, Schoevers RA, Oude Voshaar RC. Prevalence of frailty in community-dwelling older persons: a systematic review. Journal of the american geriatrics society. 2012;60(8):1487-92.
21. Curcio C-L, Henao G-M, Gomez F. Frailty among rural elderly adults. BMC geriatrics. 2014;14(1):1-9.
22. de Albuquerque Sousa ACP, Dias RC, Maciel ÁCC, Guerra RO. Frailty syndrome and associated factors in community-dwelling elderly in Northeast Brazil. Archives of gerontology and geriatrics. 2012;54(2):e95-e101.
23. Dziechciaz M, Filip R. Biological psychological and social determinants of old age: Bio-psycho-social aspects of human aging. Annals of Agricultural and Environmental Medicine. 2014;21(4).
24. Biswas T, Islam MS, Linton N, Rawal LB. Socio-economic inequality of chronic non-communicable diseases in Bangladesh. PloS one. 2016;11(11):e0167140.
25. Rahman MM, Hamiduzzaman M, Akter M, Farhana Z, Hossain MK, Hasan MN, et al. Frailty indexed classification of Bangladeshi older adults' physio-psychosocial health and associated risk factors- a cross-sectional survey study. BMC geriatrics. 2021;21(1):1-10.
26. Ali I, Azman A, Ahmed MK, Akhtar T, Deceukelier B. Assessment of the Needs of Rural Older People in Bangladesh. The International Journal of Community and Social Development. 2022;4(3):317-38.
27. Nortey ST, Aryeetey GC, Aikins M, Amendah D, Nonvignon J. Economic burden of family caregiving for elderly population in southern Ghana: the case of a peri-urban district. International journal for equity in health. 2017;16(1):1-9.
28. Kojima G, Liljas AEM, Iliffe S. Frailty syndrome: implications and challenges for health care policy. Risk management and healthcare policy. 2019:23-30.
29. Rosenberg T, Montgomery P, Hay V, Lattimer R. Using frailty and quality of life measures in clinical care of the elderly in Canada to predict death, nursing home transfer and hospitalisation-the frailty and ageing cohort study. BMJ open. 2019;9(11):e032712.
30. Perissinotto CM, Cenzer IS, Covinsky KEJAoim. Loneliness in older persons: a predictor of functional decline and death. 2012;172(14):1078-84.
31. Hossain MI, Akhtar T, Uddin MT. The elderly care services and their current situation in Bangladesh: An understanding from theoretical perspective. Journal of Medical Sciences. 2006;6(2):131-8.
32. Alam MZ, Mamun AA. Dynamics of internal migration in Bangladesh: Trends, patterns, determinants, and causes. Plos one. 2022;17(2):e0263878.
33. Kaur KJ, Sinha AK. Empty Nests: A Study on the Left-Behind Parents of Emigrated Children from Punjab (India). The Family Journal. 2023:10664807231157028.
34. Mehrabi F, Beland F. Frailty as a Moderator of the Relationship between Social Isolation and Health Outcomes in Community-Dwelling Older Adults. Int J Environ Res Public Health. 2021;18(4). Epub 2021/02/13. doi: 10.3390/ijerph18041675. PubMed PMID: 33572443; PubMed Central PMCID: PMCPMC7916171.
35. Begum A, Islam R. An Inclusive Approach to Care of the Elderly in Bangladesh. Dhaka: Bangladesh Institute of Development Studies, 2019.

Figure title

Figure 1: Prevalence of non-disabled frailty by age and gender (n=1045)

Supplementary materials

Supplementary File 1: Factors associated with non-disabled frailty and disability among the participants (N=1045)

Supplementary File 2: Dataset used for the analysis in the current study.

Response to Reviews – "The burden of non-disabled frailty and its associated factors among older adults in Bangladesh"

Dear Editor,

Thank you for allowing us to revise and resubmit the manuscript and for the constructive reviewers' comments. In the following table, we have included the reviewer's comments and outlined our responses to each comment. We have systematically incorporated the reviewer's comments and suggestions, which have improved the manuscript. We are confident that the revisions have improved the quality of our manuscript.

Kind regards,
Authors

Reviewer 1

Comment/Suggestion	Authors' Responses
**Abstract**	
Line 49. What needs?	The needs of the participants referred to their day-to-day assistance. We have mentioned this in the revised manuscript. The revised line reads as, "*Moreover, participants whose family members were non-responsive to their day-to-day assistance (RRR= 1.47, 95% CI: 1.06-2.03) were more likely to be frail compared to those whose family members were responsive.*". Please see page 3, lines 53-55.
Line 51. Who are the counterparts?	We have revised the whole Result part of the Abstract to better clarify this. Please see pages 3-4, lines 47-57.
Line 53. Previously it was mentioned that conclusion should be based on the results. However, it should not be similar to results. Your result and conclusion are almost similar. Please revise what type of tailored intervention you are talking here.	Thanks for your feedback. We have now revised the Conclusion part of the Abstract according to your suggestion. The revised lines read as, "*The findings of the present study suggest developing tailored interventions to address the burden of frailty among the older populations in Bangladesh. In particular, providing long-term care and health promotion activities*

Comment/Suggestion	Authors' Responses
	can be of value in preventing frailty and reducing adverse health outcomes among this vulnerable population group." Please see page 4, lines 60-66.
**Introduction**	
Line 71. Not clear. Need to revise the first 2 sentences.	Thanks for your suggestion. The first 2 sentences have been revised to clarify the meanings. The revised line read as, "*According to Fried and colleagues [6], frailty is a condition where three out of five phenotypic criteria of an individual are met: shrinkage (weight loss), exhaustion, weaknesses, low gait speed, and low physical activity.*". Please see page 5, lines 80-83.
Line 76. What is the biological senescence?	Thanks for noting this typo. We have corrected this in the revised manuscript. The revised line read as, "*Frailty is common in older age due to biological consequences that result in the deterioration of physical, cognitive, and psychological functions [8].*". Please see page 5, lines 85-86.
Line 81. Please revise the statement	This line has been revised. The revised line read as, "*A systematic review and meta-analysis of 56 studies conducted in LMICs documented that the prevalence of frailty and prefrailty among older adults was 17.4% and 49.3%, respectively [9].*". Please see page 6, lines 91-94.
Line 83. What is community-dwelling means?	The entire line has been revised to make it clear. The revised line read as, "*A systematic review and meta-analysis of 56 studies conducted in LMICs documented that the prevalence of frailty and prefrailty among older adults was 17.4% and 49.3%, respectively [9].*". Please see page 6, lines 91-94.
Line 84. Compared to whom?	This sentence is revised for clarification. The revised line read as,

Comment/Suggestion	Authors' Responses
	"*The prevalence of frailty was high among older adults from South Asian* countries, ranging between 25% and 65%.". Please see page 6, lines 94-95.
Line 97. Poor statement (English)	We have revised this sentence correcting the English expression. The revised line read as, "*Considering the fact that there is high burden of NCDs and limited access to health service facilities in Bangladesh, we anticipate a high prevalence of frailty among older adults.*". Please see page 6, lines 108-110.
Line 99. Which topic? Needs revision	This sentence has been revised for clarification. The revised line read as, "*Therefore, the present study was undertaken to estimate the prevalence of non-disabled frailty and its determinants among Bangladeshi older adults.*" Please see page 6, lines 110-113.
**Materials and methods**	
Line 106. What is the full form of ARCED?	The full form of ARCED, Aureolin Research, Consultancy, and Expertise Development has been added. Please see page 7, line 118.
Line 172. Not clear. Please revise.	The sentence has been revised for clarification. The revised sentence is now read as, "*The piloted questionnaire did not receive any corrections/suggestions and thus was used as it is for the data collection. Each interview took around half an hour.*". Please see page 10, lines 186-188.
Line 182. Why you did not include all potential covariates in the adjusted final model? Needs explanation.	The selection of covariates for the final regression model was undertaken using the backward elimination method based on the Akaike information criterion (AIC). We have now clarified these in revised sentences. The revised lines read as,

**Comment/Suggestion**	**Authors' Responses**
	"*Covariates for adjustment were identified using the backward elimination method based on the Akaike information criterion (AIC). The initial model was run with all potential covariates listed in Table 1, and based on the AIC, age, formal schooling, family size, residence, problems with memory or concentration, loneliness, and non-responsive family members were retained. The final model was executed including these variables.*". Please see page 10, lines 194-199.
Line 190. Ethical approval requires further revision to convey detail explanation.	We have added more information to this subsection. The new line read as, "*Participation in the survey was voluntary and participants did not receive any compensation for their time.*". Please see page 11, lines 208-209.
Line 191. Is this a protocol study?	This was not a protocol study. We have removed the term 'protocol' to clarify this. Please see page 11, line 206.
Line 215. You did not provide categorization of the disabled person.	We have now revised this for clarification. The revised line read as, "*We found that around one-fourth (24.8%) of the total participants (n=1045) had non-disabled frailty, while 19.7% had a disability.*". Please see page 14, lines 231-233.
Line 225. What is those whose? Poor English. Please revise.	The sentences has been revised to improve clarity. The revised lines read as, "*Likewise, participants living in large families with four or more members (RRR= 1.39, 95% CI: 1.01-1.92) had 39% higher risk of being frail compared to those living in small families. Also, participants whose family members were non-responsive to their day-to-day assistance (RRR= 1.47, 95% CI: 1.06-2.03) had 47%*

Comment/Suggestion	Authors' Responses
	higher risk of being frail than those who had family members responsive to their day-to-day needs.". Please see page 14-15, lines 242-247.
Line 227. Compared to whom?	We have revised relevant sentences to clarify this. The revised lines read as, "*Likewise, participants living in large families with four or more members (RRR= 1.39, 95% CI: 1.01-1.92) had 39% higher risk of being frail compared to those living in small families. Also, participants whose family members were non-responsive to their day-to-day assistance (RRR= 1.47, 95% CI: 1.06-2.03) had 47% higher risk of being frail than those who had family members responsive to their day-to-day needs.*". Please see page 14-15, lines 242-247.
**Discussion**	
Line 239. Check grammatical errors. Please revise.	We revised the sentences for clarification. The revised sentences read as, "*The present study revealed that, the current study found a 24.8% prevalence of 24.8% of the participants had non-disabled frailty and factors such as age, family size, memory or concentration problems, non-responsive family members and loneliness were associated with frailty.*". Please see page 17, lines 258-262.
Line 261. What is "in our previous studies" mean?	This sentence has been revised for clarification. The revised line reads as, "*Additionally, frailty was found to be associated with cognitive impairment, such as having problems with memory and concentration, in the present study and in some prior studies [22, 23], which may also add to the burden among older adults.*". Please see page 18, lines 283-285.

Comment/Suggestion	Authors' Responses
Line 267. Please revise to convey proper message.	The sentence has been revised to convey the message properly. The revised line read as, "*Social safety net interventions such as insurance and pensions are largely unavailable for older people in Bangladesh*." Please see page 18, lines 290-291.
Line 270. Is this from your result or from literature? If literature, where is the citation?	We removed this line in the revised manuscript. Please see page 18, lines 293-294.
Line 270. Poor standard. Please revise.	This sentence has been revised for clarification. Please see page 18-19, lines 293-298.
Line 271. Not clear.	The sentence relevant to this has been revised. The revised line read as, "Evidence suggests that Bangladeshi large families often suffer a lot in satisfying the daily needs of their family members and older family members also receive suboptimal care". Please see pages 18-19, lines 293-298.
Line 285. What is out-migration?	The sentence has been corrected. The revised line reads as, "*Second, migration is very high in Bangladesh [33]*.". Please see page 19, lines 311-312.
Line 320. Baseline for what?	The sentence has been revised accordingly. The revised line read as, "*Due to the paucity of literature on frailty in Bangladesh, the current study intended to quantify the burden and serve as a baseline for future studies*.". Please see page 21, lines 344-346.
