# Supplementary material for: The burden of non-disabled frailty and its associated factors among older adults in Bangladesh
Source: PLoS One. 2023 Nov 28;18(11):e0294889. doi: 10.1371/journal.pone.0294889 (PMC10684086; doi:10.1371/journal.pone.0294889)
Supplement: Attachment — Submitted filename: Review response_R1.docx [file pone.0294889.s003.docx]

Response to Reviews – "The burden of non-disabled frailty and its associated factors among older adults in Bangladesh"

Thank you for giving us the opportunity to revise and resubmit the manuscript and for the constructive reviewers' comments. In the following table, we have included the reviewers' comments and outlined our responses to each comment. We have systematically incorporated the reviewers' comments and suggestions, which have enhanced the manuscript. We are confident that the revisions have improved the quality of our manuscript.

**Editor comments**

| **Comment/Suggestion** | **Authors’ Responses** |
| --- | --- |
| 1. Please ensure that your manuscript meets PLOS ONE's style requirements, including those for file naming. The PLOS ONE style templates can be found at  <https://journals.plos.org/plosone/s/file?id=wjVg/PLOSOne_formatting_sample_main_body.pdf> and  <https://journals.plos.org/plosone/s/file?id=ba62/PLOSOne_formatting_sample_title_authors_affiliations.pdf>. | We confirm that our manuscript meets PLOS ONE’s style requirements. |
| 2. In your Data Availability statement, you have not specified where the minimal data set underlying the results described in your manuscript can be found. PLOS defines a study's minimal data set as the underlying data used to reach the conclusions drawn in the manuscript and any additional data required to replicate the reported study findings in their entirety. All PLOS journals require that the minimal data set be made fully available. For more information about our data policy, please see <http://journals.plos.org/plosone/s/data-availability>.  Upon re-submitting your revised manuscript, please upload your study’s minimal underlying data set as either Supporting Information files or to a stable, public repository and include the relevant URLs, DOIs, or accession numbers within your revised cover letter. For a list of acceptable repositories, please see <http://journals.plos.org/plosone/s/data-availability#loc-recommended-repositories>. Any potentially identifying patient information must be fully anonymized.  Important: If there are ethical or legal restrictions to sharing your data publicly, please explain these restrictions in detail. Please see our guidelines for more information on what we consider unacceptable restrictions to publicly sharing data: <http://journals.plos.org/plosone/s/data-availability#loc-unacceptable-data-access-restrictions>. Note that it is not acceptable for the authors to be the sole named individuals responsible for ensuring data access.  We will update your Data Availability statement to reflect the information you provide in your cover letter. | The dataset used for the analysis of the current study is uploaded as a ‘Supporting Information file.’ Please refer to Supplementary file 2. |
| 3. We note that you have indicated that data from this study are available upon request. PLOS only allows data to be available upon request if there are legal or ethical restrictions on sharing data publicly. For more information on unacceptable data access restrictions, please see <http://journals.plos.org/plosone/s/data-availability#loc-unacceptable-data-access-restrictions>.  In your revised cover letter, please address the following prompts:  a) If there are ethical or legal restrictions on sharing a de-identified data set, please explain them in detail (e.g., data contain potentially sensitive information, data are owned by a third-party organization, etc.) and who has imposed them (e.g., an ethics committee). Please also provide contact information for a data access committee, ethics committee, or other institutional body to which data requests may be sent.  b) If there are no restrictions, please upload the minimal anonymized data set necessary to replicate your study findings as either Supporting Information files or to a stable, public repository and provide us with the relevant URLs, DOIs, or accession numbers. For a list of acceptable repositories, please see <http://journals.plos.org/plosone/s/data-availability#loc-recommended-repositories>.  We will update your Data Availability statement on your behalf to reflect the information you provide. | The dataset used for the analysis of the current study will be uploaded as a ‘Supporting Information file.’ Please refer to Supplementary file 2. |
| 4. Please review your reference list to ensure that it is complete and correct. If you have cited papers that have been retracted, please include the rationale for doing so in the manuscript text, or remove these references and replace them with relevant current references. Any changes to the reference list should be mentioned in the rebuttal letter that accompanies your revised manuscript. If you need to cite a retracted article, indicate the article’s retracted status in the References list and also include a citation and full reference for the retraction notice. | We checked all references for correctness and completeness and edited them when necessary. |

**Reviewer 1**

| **Comment/Suggestion** | **Authors’ Responses** |
| --- | --- |
| The authors estimated both non-disabled frailty and disability using the FiND questionnaire but I am not clear on the reasons for the inclusion of the questions about disability or the inclusion in the results of disability and robustness. | Thank you for your queries. The FiND questionnaire determines the prevalence of frailty, disability and robustness. Thus excluding a few components, i.e., disability, is not recommended. While this research aimed to determine the prevalence and factors associated with frailty only, we could not exclude disability as it is a part of the original scale. However, we only focused on interpreting the factors associated with frailty in the regression analysis, which has been clarified on page 17, lines 309-311 in the Result section and reads as below:  *“Although findings for both non-disabled frailty and disability are presented in Table 2, given our research question, we interpret here the findings relevant to non-disabled frailty only.”* |
| The authors give some prominence to the male/female difference in non-disabled frailty yet the difference is small and does not reach statistical significance. I would recommend not giving this finding such prominence as it would be difficult to recommend policy based on this. | Thanks for noting this. We have now removed the narratives relative to the male/female difference in non-disabled frailty from the text. Please see page 3 lines 49-50 and page 17 lines 301-303. |
| Minor issues: Conclusions in the abstract; should read "present" rather than "preset". | Thank you for catching the typo. We corrected it. Please see page 4 line 61. |
| Figure 1; "Disable" should read "Disabled".  Figure 1, the Y=axis needs label (presumably %?) | Once again, thank you for catching the typo. We corrected the typo and labeled the Y-axis. |

**Reviewer 2**

| **Comment/Suggestion** | **Authors’ Responses** |
| --- | --- |
| This research on non-disabled frailty among older adults is very important for health and well-being of elderly populations and for that I think this is an important study. I have a few comments/questions that I think should be addressed properly. | Thank you for recognizing the importance of our study. We also appreciate your valuable comments and have addressed them in the manuscript. Please see our response below to each comment. |
| **Abstract** |  |
| 1. Results: Revision of the results section required for further clarity. Please check for grammatical issues also. | Thank you for your comment. We revised the results section for further clarity, and the entire manuscript has been proofread for grammar issues and typos. Please refer to page 3-4, lines 48-57 for our revision to the Result section of the abstract. |
| 2. Conclusion: Your conclusion should be based on the findings of the study (Please revise). What do you mean by “comprehensive support care package”? Typographical errors (tense, spelling, etc). | Thank you for your comment. We revised the conclusion section of the Abstract, which now reads as below on page 4, lines 61-67:  *‘The present study’s findings suggest providing long-term care tailored towards frail older adults. Specifically, those relatively older, living in large families, suffering from cognitive problems or loneliness, and not properly cared for by their family members should be targeted for relevant health promotion interventions.’* |
| **Introduction** |  |
| 3. Frailty suddenly appeared in the last sentence of the first paragraph and the link between first and second paragraph not properly done. Revision required. | Thank you for your comment. We have developed a case on frailty in paragraph 1 and established a smoother transition from paragraph 1 to paragraph 2. The first and second paragraphs of Introduction on page 5-6, lines 72-112 have been revised for clarity. |
| 4. Last sentence of the second paragraph was hard to grasp on what you were trying to convey. | Thank you for the comment. We substantially revised the second paragraph for clarity. We also noted a few unnecessary sentences in the paragraphs, which have been removed. Please see our revision in the second paragraph of Introduction on page 5-6, lines 91-112. |
| 5. Last paragraph requires attention and significant revision. Sentences beginning with “Thus” appeared in almost everywhere. | Thank you for the comment. The vocabulary and framing of sentences have been improved in the last paragraph. Please see our revision in the last paragraph of Introduction on page 7-8, lines 133-147. |
| 6. There are typographical errors (tense, spelling, grammar) that could be fixed to improve the readability of the manuscript (Introduction, Methodology, Results and Discussion). The introduction part requires significant rearrangement. Rationale should be done properly based on the objective. | Thank you for the comment. The entire manuscript has been proofread for typographical errors, and substantial revisions have been made. We have made corrections wherever applicable. The introduction section has been revamped with appropriate juxtapositions at appropriate positions (See page 5-8, lines 72-147). |
| 7. Referencing should be done properly. | Thank you for the comment. The Introduction section is now fully referenced wherever applicable. |
| **Methods and materials** |  |
| 8. Sampling framework hard to make any senses. Please revise | Thank you for your suggestion. We have revised the sampling frame, which now reads as below under the “Study design and participants” sub-section of the method. Which is now read as bellow on page 8, lines 152-159:  *“Previously, between 2016–2020, the ARCED Foundation conducted ten different community-based studies and established a registry using demographic information from these studies, which served as the sampling frame for the current study. Notably, this sampling frame included households from all eight administrative divisions of Bangladesh.”* |
| 9. Sample size based on the “response rate” and overall “response rate “creating confusion. Please revise accordingly. | Thank you for your suggestion. We have revised the sample size statement, which now reads as below under the “Study design and participants” sub-section of the Method on page 8-9, lines 159-163:  *“Considering a 50% unknown prevalence with a 5% margin of error, 95% confidence level, and 90% power, a sample size of 1096 was calculated. However, only 1045 of 1096 approached participants responded to the study resulting in an overall response rate of approximately 95%.”* |
| 10. “The inclusion criterion was the minimum age of 60 years” of whom? Please check the whole manuscript for sentence making so that conveying messages make senses. | Thank you for your suggestion. We have revised the sentence to indicate the minimum age of participants (See page 9, line 166).  We also proofread the entire manuscript for grammar and sentence structure. |
| 11. First sentence of the outcome measure is hard to grasp. Please revise. | Thank you for your suggestion. We have revised the sentence which reads as below on page 9, lines 172-173:  *‘The outcome of interest was non-disabled frailty, measured using the ‘Frail Non-Disabled (FiND) questionnaire’ [18]’.* |
| 12. Multiple grammatical errors in this section. | Thank you for bringing that to our attention. We proofread the entire manuscript for grammar and sentence structure. |
| 13. What is “robust”? | ‘Robust’ is a category identified through the FiND questionnaire, which represents the absence of ‘frailty’ or ‘disability.’ We revisited the outcome variable definition to indicate the meaning of robust explicitly. Please refer to our revision on page 9-10, lines 172-189. |
| 14. Explanatory variables- what do you mean by “without partner” in the “marital status (married/without a partner)” variable? | Thank you for bringing that to our attention. We revised the statement to indicate that the latter category, i.e., without a partner included widowed, separated and never married. Please see our revision on page 10, lines 195-196 in the revised manuscript. |
| 15. How many research assistants you had recruited for the study? Did you provide any training before the actual data collection? If yes, how long? | Thank you for your suggestion. We have revised the manuscript to indicate the number and training of research assistants. Our revision includes the following sentences under the sub-heading “Data collection tools and techniques” of Methods on page 11, lines 224-228.  *“Ten research assistants were recruited as surveyors based on their previous experiences administering health surveys on the electronic platform. The research assistants were trained extensively via Zoom meetings for three full days by the coauthors (SKM, AMA, UNY) before beginning the data collection.”* |
| 16. Data collection tools and techniques require significant revision. | Thank you for your suggestion. We revised the  ‘Data collection tools and techniques’ section for clarity. Please see our changes on page 11-12, lines 222-238. |
| 17. Statistical analyses lack proper guidance what you have done in the paper. | Thank you for your suggestion. We revised the  ‘Statistical analyses’ section for clarity. Please see our changes on page 12-13, lines 241-258. |
| 18. Ethics approval: “All procedures performed in studies….” Did not understand? Second sentence failed to convey messages properly. Please revise. | Thank you for your suggestion. We revised the  ‘Ethics approval’ section for clarity. Please see our changes on page 13, lines 261-266. |
| **Results** |  |
| 19. Characteristics of the participants- Please revise with most important information that aligns with your objectives. | Thank you for your suggestion. We revised the  ‘Characteristics of the participants’ section for clarity. Please see our changes on page 13-14, lines 274-291. |
| 20. 19.7% were considered disabled (more than 200 people out of 1045!). This is huge number. Can you please share what types of disability you found in this study? | The prevalence of disability was measured using the FiND questionnaire, which defines disability as an affirmative answer to at least one of the following questions. Thus, disability is defined in terms of mobility of the participants. For your convenience link to the questionnaire is also provided below.  A. Have you any difficulties at walking 400 meters?  B. Have you any difficulties at climbing up a flight of stairs?  <https://frailty.net/wp-content/uploads/FiND_Questionnaire_English.pdf> |
| 21. “perceiving family members to be non-responsive to their needs (RRR= 1.47, 95% CI: 1.06-2.03)….” what do you mean by “perceiving family members”? | Thank you for your suggestion. The sentence was revised and now reads as below on page 17-18, lines 316-317.  ‘………………..and those whose family members were non-responsive to their needs (RRR= 1.47, 95% CI: 1.06-2.03) had……………..’ |
| 22. What is “concentration”? Provide the definitions of “low memory” and “concentration”. | Thank you for your suggestion. We added a statement defining this variable in the methods section under “Explanatory variables” on page 11, lines 210-212. |
| 23. Please revise this statement- “participants who reported a feeling of loneliness had a significantly higher risk of frailty compared to those who did not feel so”. | Thank you for your suggestion. The sentence was revised and now reads as below on page 18, lines 320-322.  *‘Moreover, participants reporting loneliness were significantly more likely to be frail than their counterparts (RRR= 1.45, 95% CI: 1.07-1.98).’* |
| **Discussion** |  |
| 24. Did not understand the first paragraph of the discussion section. Please revise to convey the messages properly. | Thank you for your suggestion. We revised the first paragraph of the ‘Discussion’ section for clarity. This now reads as below on page 20, lines 332-341:  *‘With the aim to examine the prevalence and determinants of non-disabled frailty among Bangladeshi older people, the current study found a 24.8% prevalence of non-disabled frailty and factors such as age, family size, memory or concentration problems, non-responsive family members and loneliness associated with frailty. Our study contributes to the limited literature on frailty among South Asian older adults, specifically from Bangladesh.*’ |
| 25. “The discrepancies in frailty prevalence between the study and the literature may be attributable to methodological factors such as…” and after that you provided examples of determinants not the methodological factors. This paragraph requires significant revision. | Thank you for bringing that to our attention. The sentence was revised and now reads as below on page 21, lines 348-354.  *‘The discrepancies in frailty prevalence between the current study and the existing literature may be attributable to several factors, including different measurement tools, heterogeneity in participants’ ages, people’s living standard, and the burden of NCDs.’* |
| 26. “In our study, the odds of frailty were higher in the higher age group ….” In the results, you mentioned about RRR. Here you are talking about odds? Why, revise accordingly. | Thank you for pointing that out. We have revised the line, which now reads as below on page 21, lines 356-358:  *“In our study, older people aged ≥ 80 were more likely to be frail than their younger counterparts, which aligns with existing literature [22, 23].”* |
| 27. “Like many other countries of the world, Bangladesh is also facing an epidemiological transition” What are the countries you are talking about? Rich, poor, developing, developed? | Thank you for pointing that out. We have specified the countries and added corresponding citations. The revised sentence on page 21, lines 362-365 now reads as below:  *“Like many other countries of the world (e.g., India, China and Indonesia), Bangladesh is also facing an epidemiological transition, resulting in a higher burden of NCDs and multimorbidity, particularly among the older population [25], which might have contributed to a higher level of frailty among the older ages.”* |
| 28. “We found that older adults living in a large family and reported having their family members non-responsive to their care had a higher chance of frailty. These two things are interlinked….” How they are interlinked? Did you analyze that? Did you mention that in the results? | Thank you for pointing that out. We restructured the discussion to establish the interlink between the two variables. Please refer to the fourth discussion paragraph, page 22-23, lines 371-391, for our revision. |
| 29. Explanation of the fourth paragraph of the discussion section is not comprehensive and logical enough. Please provide scientific explanations with relevant references. | Thank you for pointing that out. We restructured the fourth paragraph with evidence from the literature. Please refer to the fourth discussion paragraph, page 22-23, lines 371-391, for our revision. |
| 30. “After a certain period of life, depending financially, emotionally, and physically on children and receiving care from there is culturally expected and appropriate.” Don’t understand what you are trying to convey here? | Thank you for pointing that out. We have revised the paragraph substantially. Please refer to page 23-24, lines 393-422 for our revision. |
| 31. Need more elaborations on limitations of the study. | We have added statements to the existing limitations. Please refer to page 25-26, lines 454-470 for our revision. |
| **Conclusion** |  |
| 32. “Overall, the findings of the study suggest analyzing and understanding frailty and its associated components is an essential first step toward that preventive goal” What are the associated components you are trying to imply here? | Thank you for your suggestion. We revised the conclusion section for clarity. Please refer to page 26-27, lines 473-489 for our revision. |
| 33. Please revise the conclusion section for better understanding. | Thank you for your suggestion. We revised the conclusion section for clarity. Please refer to to page 26-27, lines 473-489 for our revision. |
