# Supplementary material for: The burden of non-disabled frailty and its associated factors among older adults in Bangladesh
Source: PLoS One. 2023 Nov 28;18(11):e0294889. doi: 10.1371/journal.pone.0294889 (PMC10684086; doi:10.1371/journal.pone.0294889)
Supplement: Attachment — Submitted filename: Review response_R2.docx [file pone.0294889.s005.docx]

Response to Reviews – "The burden of non-disabled frailty and its associated factors among older adults in Bangladesh"

Dear Editor,

Thank you for allowing us to revise and resubmit the manuscript and for the constructive reviewers' comments. In the following table, we have included the reviewer’s comments and outlined our responses to each comment. We have systematically incorporated the reviewer's comments and suggestions, which have improved the manuscript. We are confident that the revisions have improved the quality of our manuscript.

Kind regards,

Authors

**Reviewer 1**

| **Comment/Suggestion** | **Authors’ Responses** |
| --- | --- |
| **Abstract** |  |
| Line 49. What needs? | The needs of the participants referred to their day-to-day assistance. We have mentioned this in the revised manuscript. The revised line reads as, “*Moreover, participants whose family members were non-responsive to their day-to-day assistance (RRR= 1.47, 95% CI: 1.06-2.03) were more likely to be frail compared to those whose family members were responsive.*”. Please see page 3, lines 53-55. |
| Line 51. Who are the counterparts? | We have revised the whole Result part of the Abstract to better clarify this. Please see pages 3-4, lines 47-57. |
| Line 53. Previously it was mentioned that conclusion should be based on the results. However, it should not be similar to results. Your result and conclusion are almost similar. Please revise what type of tailored intervention you are talking here. | Thanks for your feedback. We have now revised the Conclusion part of the Abstract according to your suggestion. The revised lines read as, “*The findings of the present study suggest developing tailored interventions to address the burden of frailty among the older populations in Bangladesh. In particular, providing long-term care and health promotion activities can be of value in preventing frailty and reducing adverse health outcomes among this vulnerable population group.*” Please see page 4, lines 60-66. |
| **Introduction** |  |
| Line 71. Not clear. Need to revise the first 2 sentences. | Thanks for your suggestion. The first 2 sentences have been revised to clarify the meanings. The revised line read as, “*According to Fried and colleagues [6], frailty is a condition where three out of five phenotypic criteria of an individual are met: shrinkage (weight loss), exhaustion, weaknesses, low gait speed, and low physical activity.*”. Please see page 5, lines 80-83. |
| Line 76. What is the biological senescence? | Thanks for noting this typo. We have corrected this in the revised manuscript. The revised line read as, “*Frailty is common in older age due to biological consequences that result in the deterioration of physical, cognitive, and psychological functions [8].*”. Please see page 5, lines 85-86. |
| Line 81. Please revise the statement | This line has been revised. The revised line read as, “*A systematic review and meta-analysis of 56 studies conducted in LMICs documented that the prevalence of frailty and prefrailty among older adults was 17.4% and 49.3%, respectively [9].*”. Please see page 6, lines 91-94. |
| Line 83. What is community-dwelling means? | The entire line has been revised to make it clear. The revised line read as, “*A systematic review and meta-analysis of 56 studies conducted in LMICs documented that the prevalence of frailty and prefrailty among older adults was 17.4% and 49.3%, respectively [9].*”. Please see page 6, lines 91-94. |
| Line 84. Compared to whom? | This sentence is revised for clarification. The revised line read as, “*The prevalence of frailty was high among older adults from South Asian* countries, ranging between 25% and 65%*.*”. Please see page 6, lines 94-95. |
| Line 97. Poor statement (English) | We have revised this sentence correcting the English expression. The revised line read as, “*Considering the fact that there is high burden of NCDs and limited access to health service facilities in Bangladesh, we anticipate a high prevalence of frailty among older adults.*”. Please see page 6, lines 108-110. |
| Line 99. Which topic? Needs revision | This sentence has been revised for clarification. The revised line read as, “*Therefore, the present study was undertaken to estimate the prevalence of non-disabled frailty and its determinants among Bangladeshi older adults.*” Please see page 6, lines 110-113. |
| **Materials and methods** |  |
| Line 106. What is the full form of ARCED? | The full form of ARCED, Aureolin Research, Consultancy, and Expertise Development has been added. Please see page 7, line 118. |
| Line 172. Not clear. Please revise. | The sentence has been revised for clarification. The revised sentence is now read as, “*The piloted questionnaire did not receive any corrections/suggestions and thus was used as it is for the data collection. Each interview took around half an hour.*”. Please see page 10, lines 186-188. |
| Line 182. Why you did not include all potential covariates in the adjusted final model? Needs explanation. | The selection of covariates for the final regression model was undertaken using the backward elimination method based on the Akaike information criterion (AIC). We have now clarified these in revised sentences. The revised lines read as, “*Covariates for adjustment were identified using the backward elimination method based on the Akaike information criterion (AIC). The initial model was run with all potential covariates listed in Table 1, and based on the AIC, age, formal schooling, family size, residence, problems with memory or concentration, loneliness, and non-responsive family members were retained. The final model was executed including these variables.*”. Please see page 10, lines 194-199. |
| Line 190. Ethical approval requires further revision to convey detail explanation. | We have added more information to this subsection. The new line read as, “*Participation in the survey was voluntary and participants did not receive any compensation for their time.*”. Please see page 11, lines 208-209. |
| Line 191. Is this a protocol study? | This was not a protocol study. We have removed the term ‘protocol’ to clarify this. Please see page 11, line 206. |
| Line 215. You did not provide categorization of the disabled person. | We have now revised this for clarification. The revised line read as, “*We found that around one-fourth (24.8%) of the total participants (n=1045) had non-disabled frailty, while 19.7% had a disability.*”. Please see page 14, lines 231-233. |
| Line 225. What is those whose? Poor English. Please revise. | The sentences has been revised to improve clarity. The revised lines read as, “*Likewise, participants living in large families with four or more members (RRR= 1.39, 95% CI: 1.01-1.92) had 39% higher risk of being frail compared to those living in small families. Also, participants whose family members were non-responsive to their day-to-day assistance (RRR= 1.47, 95% CI: 1.06-2.03) had 47% higher risk of being frail than those who had family members responsive to their day-to-day needs.*”. Please see page 14-15, lines 242-247. |
| Line 227. Compared to whom? | We have revised relevant sentences to clarify this. The revised lines read as, “*Likewise, participants living in large families with four or more members (RRR= 1.39, 95% CI: 1.01-1.92) had 39% higher risk of being frail compared to those living in small families. Also, participants whose family members were non-responsive to their day-to-day assistance (RRR= 1.47, 95% CI: 1.06-2.03) had 47% higher risk of being frail than those who had family members responsive to their day-to-day needs.*”. Please see page 14-15, lines 242-247. |
| **Discussion** |  |
| Line 239. Check grammatical errors. Please revise. | We revised the sentences for clarification. The revised sentences read as, “*The present study revealed that, the current study found a 24.8% prevalence of 24.8% of the participants had non-disabled frailty and factors such as age, family size, memory or concentration problems, non-responsive family members and loneliness were associated with frailty.*”. Please see page 17, lines 258-262. |
| Line 261. What is “in our previous studies” mean? | This sentence has been revised for clarification. The revised line reads as, “*Additionally, frailty was found to be associated with cognitive impairment, such as having problems with memory and concentration, in the present study and in some prior studies [22, 23], which may also add to the burden among older adults.*”. Please see page 18, lines 283-285. |
| Line 267. Please revise to convey proper message. | The sentence has been revised to convey the message properly. The revised line read as, “*Social safety net interventions such as insurance and pensions are largely unavailable for older people in Bangladesh*.” Please see page 18, lines 290-291. |
| Line 270. Is this from your result or from literature? If literature, where is the citation? | We removed this line in the revised manuscript. Please see page 18, lines 293-294. |
| Line 270. Poor standard. Please revise. | This sentence has been revised for clarification. Please see page 18-19, lines 293-298. |
| Line 271. Not clear. | The sentence relevant to this has been revised. The revised line read as, “Evidence suggests that Bangladeshi large families often suffer a lot in satisfying the daily needs of their family members and older family members also receive suboptimal care”. Please see pages 18-19, lines 293-298. |
| Line 285. What is out-migration? | The sentence has been corrected. The revised line reads as, “*Second, migration is very high in Bangladesh [33]*.”. Please see page 19, lines 311-312. |
| Line 320. Baseline for what? | The sentence has been revised accordingly. The revised line read as, “*Due to the paucity of literature on frailty in Bangladesh, the current study intended to quantify the burden and serve as a baseline for future studies*.”. Please see page 21, lines 344-346. |
